# Navigating Uncertainties in RT-qPCR and Infectivity Assessment of Norovirus

**DOI:** 10.1007/s12560-024-09632-0

**Published:** 2025-03-08

**Authors:** Razieh Sadat Mirmahdi, Samantha L. Dicker, Nuradeen Garba Yusuf, Naim Montazeri

**Affiliations:** 1 Food Science and Human Nutrition Department, University of Florida, 572 Newell Drive, Gainesville, FL 32611 USA; 2 Global Food Systems Institute, University of Florida, Gainesville, FL USA

**Keywords:** Food safety, Infectivity assessment, Norovirus, Public health, Surrogate, Tulane virus

## Abstract

Human norovirus (HuNoV) is the primary cause of gastroenteritis globally. Due to the lack of a reliable cultivation system, RT-qPCR is a gold standard technique for the detection and quantification of HuNoV. However, the inability of PCR to differentiate between infectious from non-infectious particles remains a significant limitation. This study aims to address this limitation by exploring the relationship between culture-based (plaque assay and TCID_50_) and non-culture-based (RT-qPCR) methods for HuNoV quantification, using Tulane virus as a cultivable surrogate. The ultracentrifuge-purified Tulane virus at 6.7 log_10_ PFU/ml or 5.8 log_10_ TCID_50_/ml in Tris–EDTA buffer (pH 7.2), was serially diluted and subjected to RNA extraction, with or without RNase pretreatment, followed by quantification with RT-qPCR. Further physical characterization of the virus stock was performed with dynamic light scattering and transmission electron microscopy. A strong correlation (Pearson’s Correlation Coefficient of 0.99) was observed between log_10_ genome copies (GC) and log_10_ plaque forming units (PFU) per PCR reaction for both RNase-pretreated and unpretreated samples. Beta distributions indicated a similar median GC:PFU ratio of ca. 3.7 log_10_ for both RNase-pretreated and unpretreated samples. The high GC:PFU ratio may indicate the sensitive nature of RT-qPCR or the presence of intact, non-infectious virus particles. The outcomes of this study will contribute to the more accurate estimation of infectious norovirus particles in food and environmental matrices.

**Graphical Abstract:**

**Figure:**
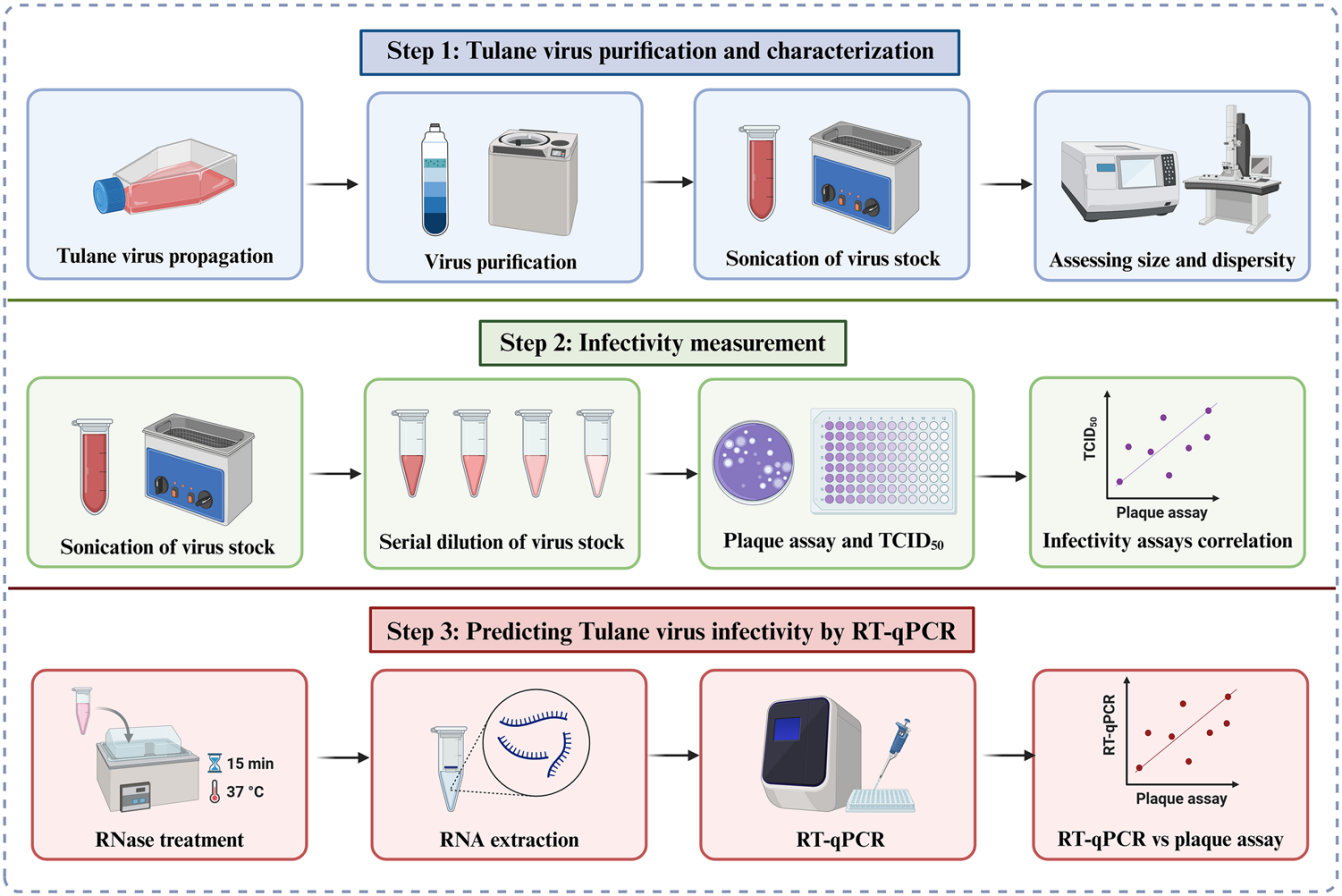
Created in BioRender. Mirmahdi, R. (2024) https://BioRender.com/l49a583

**Supplementary Information:**

The online version contains supplementary material available at 10.1007/s12560-024-09632-0.

## Introduction

Human norovirus (HuNoV) is the leading cause of viral foodborne illnesses worldwide (FAO/WHO, 2023). As an enteric pathogen, HuNoV is mainly shed in the feces of infected individuals. Consequently, fecally contaminated foods pose the highest risks of foodborne transmission to humans (Carlson et al., 2024; Teunis et al., 2015). The food commodities most commonly associated with HuNoV infections are ready-to-eat products, frozen berries, and shellfish (FAO/WHO, 2023; US CDC, 2024). In the absence of an accessible culture-based technique for HuNoV detection, reverse-transcription polymerase chain reaction (RT-PCR) remains the global gold standard for clinical diagnostics and regulatory operational procedures for monitoring food safety (ISO, 2017; US FDA, 2024; Vinjé, 2015). While the prevalence of HuNoV in high-risk foods is generally low, the highly infectious nature of HuNoV has led to recalls and market withdrawals when HuNoV genetic material is detected (Pouillot et al., 2022; Steele et al., 2022). Surveillance studies of HuNoV in berries have often reported RT-qPCR signals near or below the limit of detection with cycle thresholds (Ct) as high as 42 (Gummalla et al., 2024; Steele et al., 2022; US FDA, 2020). The Ct value in RT-qPCR is inversely correlated with the concentration of target RNA in the samples. As thermal cycles progress, the efficacy of amplification usually decreases at Ct values higher than 40, diminishing assay sensitivity (Bustin et al., 2009; Trang et al., 2015). Given that the concentration of HuNoV in food is commonly low, this can lead to significant uncertainty in the collected data. Furthermore, a fundamental limitation of RT-qPCR for HuNoV detection is its inability to distinguish infectious from non-infectious virus particles or freely available viral RNA in the sample, which may lead to false-positive signals (Knight et al., 2013). On the other hand, despite recent advancements in HuNoV culture systems and the use of stem cell-derived human enteroids, zebrafish larvae, and B cells for the virus propagation, several limitations persist, including challenges in passaging HuNoV in any culture system, its dependence on stool samples, limited replication, and the complexity of the process (Hayashi et al., 2024; Van Dycke et al., 2019). The continued reliance on RT-qPCR for monitoring HuNoV in high-risk foods introduces significant uncertainties when estimating viral doses for quantitative microbial risk assessments or decision-making regarding the suitability of food products for consumption (Cook et al., 2016; Lopman et al., 2016; Teunis et al., 2008).

Culturable surrogate viruses, such as Tulane virus and murine norovirus, have been widely used as surrogates for HuNoV to assess virus infectivity in persistence and inactivation studies (Anderson-Coughlin et al., 2023; Cromeans et al., 2014). Two commonly used methods for estimating infectious virus particles in vitro are the plaque assay and 50% tissue culture infective dose (TCID_50_) assay, both relying on cytopathic effects (CPE) (Shan et al., 2016). Plaque assay provides an absolute quantification by counting plaque forming units (PFU), whereas the TCID_50_ assay is an endpoint dilution technique that estimates the concentration of infectious virus particles required to infect 50% of the host cells (Lei et al., 2021; Smither et al., 2013). Although infectivity assays provide a greater understanding of infectious virus particles than RT-qPCR alone, neither method can accurately estimate the total number of infectious viral particles. In some cases, more than one virion may be required to infect a single host cell, leading to varying particle-to-PFU ratios (Sanjuán, 2018). This inefficiency in viral infectivity is influenced by virus type, environmental conditions, and the forms in which the virus is present, as dispersed particles, aggregate, those bound to particles, or embedded inside vesicles (Gerba & Betancourt, 2017; Sanjuán, 2018; Stein et al., 1970; Zhang et al., 2021). As a result, these factors introduce uncertainty in the quantification of infectious virus particles.

While RT-qPCR is unable to distinguish between infectious and non-infectious virus particles (Oristo et al., 2018), it offers higher sensitivity in virus detection compared to infectivity assays. Inactivation studies showing a greater reduction in virus titer in infectivity assays compared to RT-qPCR suggests that RT-qPCR may overestimate the number of residual virus particles and, consequently, the risk to public health (Nuanualsuwan & Cliver, 2002; Shan et al., 2016). However, this assumption does not account for the situations where virus preparation has a high particle-to-PFU ratio. Alternative solutions include the pretreatment of virus extract with compounds such as RNase or propidium monoazide (PMA), before RNA extraction enhances the quantification of viral RNA from intact, presumptively infectious viral particles (Nuanualsuwan & Cliver, 2002; Oristo et al., 2018). Determining the association of RT-qPCR signals with the concentration of infectious virus particles is crucial for developing more robust risk assessment models in studies involving the detection of HuNoV using RT-qPCR.

To address some of these uncertainties, the primary goal of this study was to examine the relationship between virus infectivity assay and RT-qPCR on serially diluted virus stocks. Tulane virus was used as a surrogate for HuNoV, representing a closely related cultivable virus surrogate to extrapolate PCR data to infectivity assays. This research investigates the efficacy of RNase pretreatment prior to RT-qPCR in detecting intact, presumptively infectious virus particles by inhibiting the amplification of exposed RNA. Virus concentrations were adjusted based on a 25-µl RT-qPCR reaction volume to allow a direct correlation between the PCR signal and the number of infectious particles. The ratios of TCID_50_ and genome copy (GC) to PFU provide a basis for predicting virus infectivity when absolute quantification through culture-based methods is unavailable. Recognizing the inherent variability of these relationships, simulated modeling was employed to gain additional insights into the uncertainties surrounding these predictions. Collectively, the findings offer perspectives on interpreting conventional techniques for the detection and quantification of HuNoV in food and environmental matrices, ultimately contributing to improved food safety and public health outcomes.

## Materials and Methods

### Virus Propagation and Purification

Tulane virus was provided by L.A. Jaykus (North Carolina State University, Raleigh) and was propagated by infecting a 90% confluent monolayer of the rhesus monkey kidney cell line (LLC-MK2, ATCC CCL-7™) at a multiplicity of infection of 0.1 (Farkas et al., 2008). The cells were incubated with low-serum improved minimal essential medium (Gibco Opti-MEM™, Thermo Fischer Scientific, Grand Island, NY) supplemented with 2% *v/v* fetal bovine serum (Gibco FBS, Thermo Fischer Scientific) at 37 °C in a 5% CO₂ atmosphere. Once the CPEs were observed after three days of incubation, viruses were recovered through three freeze–thaw cycles, followed by removing cell particles through centrifugation at 12,000 × g for 20 min at 18 °C, then passing through a 0.2-µm filter. Virus lysate was concentrated using an Amicon**®** Ultra-15 filter (50 kDa nominal molecular weight limit (NMWL), Millipore, Carrigtwohill, Ireland) and subjected to density gradients ultracentrifugation for further purification using iodixanol (OptiPrep™, Cosmo Bio USA, Carlsbad, CA) at 15–60% *v/v* layers. Ultracentrifugation was performed with Sorvall WX80 Plus (Thermo Scientific™, Santa Clara, CA) with a TH-641 swinging bucket rotor (Thermo Scientific™) and under the conditions explained previously (Barnes et al., 2021). The 40% and 60% interface and the 60% iodixanol layer were recovered and buffer-exchanged with 10 mM Tris-1 mM EDTA (pH 7.5) using an Amicon**®** Ultra-4 (30 kDa NMWL, Millipore). The virus concentrate was stored at −80 °C until needed.

Plaque assay was performed on MK2-LLC monolayer cells seeded in 60 mm cell culture dishes (Thermo Fisher Scientific) following the previously reported procedure with minor modifications (Barnes et al., 2021). Briefly, serially diluted samples were added to the cellular monolayers. After a 1-h incubation at 37 °C and 5% CO₂, the plates were overlaid with a mixture of 1.5% *w/v* SeaPlaque® agarose (Lonza, Basel, Switzerland) and complete Opti-MEM™ medium containing 1.6% *v/v* FBS. The infected cells were incubated for 72 h at 37 °C and 5% CO_2_ atmosphere. Then, the cells were fixed with 3.7% *v/v* formaldehyde (Thermo Fisher Scientific), stained with 0.1% *v/v* crystal violet (Sigma Aldrich, St. Louis, MO), and the plaque forming units (PFU) were counted. For each experiment, the working virus stock (6.7 log_10_ PFU per ml), corresponding to 4.8 log_10_ PFU per RT-qPCR reaction, was prepared in Tris–EDTA buffer, then subjected to sonication in an ultrasonic bath at 150W (40 kHz) for 4 min (Branson®, Model CPX1800H, Mexico), before being subjected to testing.

### Virus Characterization

Transmission electron microscopy (TEM) and dynamic light scattering (DLS) were used to assess the integrity of the purified virus stocks. The TEM imaging was conducted using FEI Tecnai™ G2 Spirit Twin (FEI Corp., Hillsboro, OR) to visualize virus particles. Briefly, glow-discharged, 400 mesh carbon-coated copper grids (CF-400-Cu, Electron Microscopy Sciences, Hatfield, PA) were floated on 10 µl of suspension for 5 min. Excess solution was blotted from the grid using filter paper. The sample was then washed by touching the grid to drops of ultrapure water three times, followed by floating it on a drop of 1% aqueous uranyl acetate (Electron Microscopy Sciences, Hatfield, PA) for 30 s. After blotting dry, digital images were captured using a Gatan UltraScan 2 k × 2 k camera and Digital Micrograph software (Gatan Inc., Pleasanton, CA). All preparations were done at room temperature, and observations were made at the appropriate magnification. The size distribution of virus particles was measured using Dynamic Light Scattering (DLS) with a Zetasizer Ultra (Malvern Panalytical, Westborough, MA) and a Capillary Cell ZSU1002 at 25 °C in triplicate. Data were obtained using the ZS Explorer software (version 3.31).

### Virus Infectivity Assessment

Quantitation of infectious virus particles was performed using plaque assay and TCID_50_ techniques. The steps for the plaque assay were described earlier. For the TCID_50_ assay, 96-well Nunc™ MicroWell™ Microplates (Thermo Fisher Scientific) were seeded with 2 × 10^4^ MK2 cells per well. Once the cells reached 90% confluency, the cell growth medium was replaced with 50 µl of serially diluted virus in base medium, with five wells used per dilution. After incubation for 1 h at 37 °C in a 5% CO₂ atmosphere, 150 µl of complete Opti-MEM™ medium was added to each well, and the plates were incubated until CPEs were observed. The cells were then fixed with 3.7% *v/v* formaldehyde and then stained with 0.1% *w/v* crystal violet. Wells with distinct CPE were considered positive for infection. The TCID_50_ per ml was calculated based on the number of positive and negative wells for each dilution using the Reed and Muench method (Lei et al., 2021).

### Quantitative Real-Time RT-qPCR Assay

The working stock of Tulane virus (6.7 log_10_ PFU per ml) was subjected to RT-qPCR with and without pretreatment with RNase ONE™ Ribonuclease (Promega, Madison, WI), following the method previously described (Barnes et al., 2021; Marti et al., 2017). Viral RNA was extracted using the RNeasy® Mini kit (Qiagen, Hilden, Germany), following the manufacturer’s protocol with a minor modification by adding β-mercaptoethanol (ACROS Organics, Germany) to the lysis buffer at a final concentration of 2.8% *v/v* to enhance inactivation of RNase. The extracted RNA was then subjected to RT-qPCR in triplicate using Luna® Universal Probe One-Step RT-qPCR kit (New England Biolabs, Ipswich, MA) using a CFX96 Touch™ Real-time PCR detection system (Bio-Rad, Hercules, CA). Included in the 25-µl of the master mix were 3-µl of template RNA, 25 Units of the murine RNase inhibitor (New England Biolabs), and 0.25 µM of primers and probes (Table 1). The RT-qPCR conditions were as follows: reverse transcription for 10 min at 55 °C, *Taq* polymerase activation for 1 min at 95 °C, followed by 45 cycles of 15 s at 95 °C, 30 s at 54 °C, and 30 s at 72 °C (Barnes et al., 2021). Genomic copies of the Tulane virus were quantified using calibration curves established through RT-qPCR assays of serially diluted RNA transcripts generated with a T7 transcription kit (MEGAshortscript™ T7 transcription kit, Invitrogen™, Austin, TX), as described previously (Escudero et al., 2012). The RNA transcripts were suspended in the Ambion™ RNA Storage Solution (Invitrogen™, Carlsbad, CA, United States) and stored at −80 °C until needed. The concentration of the transcripts was measured using a Qubit™ RNA High Sensitivity (HS) Assay Kit (Invitrogen™, Carlsbad, CA, United States) with a Qubit™ 4 Fluorometer (Invitrogen™, Singapore). Genome copy numbers were then calculated using the NEBiocalculator® converter tool (nebiocalculator.neb.com, New England Biolabs).

**Table 1:** RT-qPCR primers and probes and their sequences

Primer & Probes	Sequence (5′ to 3′)	Polarity	Reference
Primer TVF	GAG ATT GGT GTC AAA ACA CTC TTT G	Positive	(Sestak et al., 2012)
Primer TVR	ATC CAG TGG CAC ACA CAA TTT	Negative	
Probe TVP	FAM-AGT TGA TTG ACC TGC TGT GTC A-BHQ1	Positive	

### Statistical Analyses

All experiments were conducted with three biological and two technical replicates, and the collected data are reported as mean ± standard error or with mean with 95% confidence or prediction intervals [lower, upper], whenever applicable. Statistical analyses and visualizations were conducted using RStudio version 4.4.2 (R Core Team, 2024), leveraging a suite of available packages in the depository. Initial data entry and processing were handled with the *base* packages (R Core Team, 2024) and additional packages, including *readxl* (Wickham & Bryan, 2023), *dplyr* (Wickham et al., 2023), *tidyr* (Wickham et al., 2024), *writexl* (Ooms, 2021).

The relationships between measurement techniques were analyzed using regression and correlation analyses, with ratios between the measurement techniques reported in both log_10_ and anti-log_10_ forms. Linear and second-order polynomial regression models were fitted to the data to establish relationships between variables, followed by an assessment of residuals for normality and homoscedasticity. The best-fitting models were selected based on diagnostic plots, Root Mean Square Error (RMSE), and the Corrected Akaike Information Criterion (AICc) using the *AICcmodavg* package (Mazerolle, 2023) according to the criteria outlined by Burnham et al. (2011). Models with lower RMSE and AICc, which penalize complexity based on the number of variables, were considered the most plausible fits. To quantify uncertainty in the statistical analyses, 95% confidence and prediction intervals were calculated for the predicted values. Bland–Altman analysis, performed using the *blandr* package (Datta, 2017), was used to compare the performance between the two measurement methods. This analysis quantified the bias (mean differences) and limits of agreement (LOA) between the techniques, providing further insights into potential systematic differences in their predictive capabilities (Datta, 2017).

To establish an association between log_10_ GC and log_10_ PFU per PCR reaction, Pearson’s product-moment correlation coefficients, along with significant tests, were calculated separately for samples with and without RNase pretreatments using the *cocor* package (Diedenhofen & Musch, 2015). The predictive powers of TCID_50_ and GC (with and without RNase pretreatments) for estimating PFU in samples were assessed by calculating the TCID_50_:PFU and GC:PFU ratios. To assess the predictive analytic model, the dataset was randomly partitioned into training (70%) and testing (30%) sets, and this process was repeated across five partitions with 500 iterations to account for variability in data. For each iteration, the trained model was validated on the testing set. The performance of each model was evaluated by calculating the RMSE and Coefficient of Determination (R^2^).

The log_10_ transformed TCID_50_:PFU and GC:PFU were scaled between 0 and 1 using min–max normalization and fit to Beta distributions using *fitdistrplus* package (Delignette-Muller & Dutang, 2015). A bootstrap simulation with 1,000 iterations was then performed to estimate the uncertainty of the distribution parameters. This approach provided a probabilistic framework to calculate the confidence intervals (uncertainty), median values, and other estimates that are critical for evaluating prediction accuracy and understanding the potential data variability in ratio-based measurements. To enhance interpretability, the x-axis of the plots was rescaled to reflect the original log_10_ GC:PFU ratios. The Beta distributions for with and without RNase pretreatments were compared using an asymptotic two-sample Kolmogorov–Smirnov test to assess differences in their cumulative distribution functions and a Wilcoxon rank sum test (Mann–Whitney U test) to compare their central tendencies.

Where applicable, the statistical significance was assessed using two-sided t-tests with a significance level of *⍺* = 0.05. Data normality was evaluated prior to the analysis using the Shapiro–Wilk test. For pairwise comparisons between groups, *p*-values were adjusted using the Bonferroni correction to account for the increased likelihood of Type I errors resulting from multiple comparisons. All data visualizations were generated using the *ggplot2* package (Wickham, 2016) and the *ggpubr* package (Kassambara, 2023). The associated data files and R codes are provided in the supplementary materials for reproducibility.

## Results

### Virus Stock Characterization

**Fig. 1:**
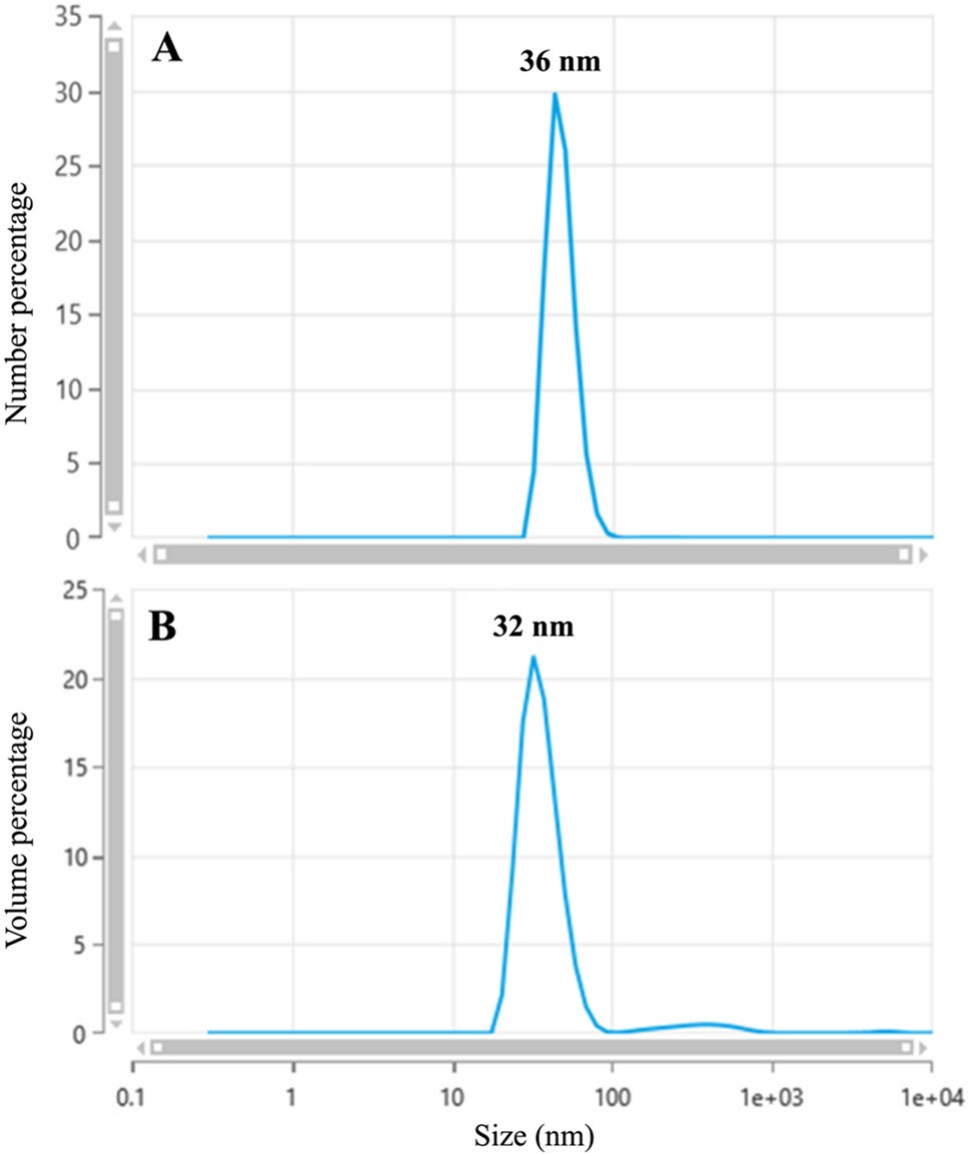
Size distribution of Tulane virus particles at 9.0 log_10_ PFU per ml with Dynamic Light Scattering (DLS) based on number (**A**) and volume (**B**) distributions of the particles

**Fig. 2:**
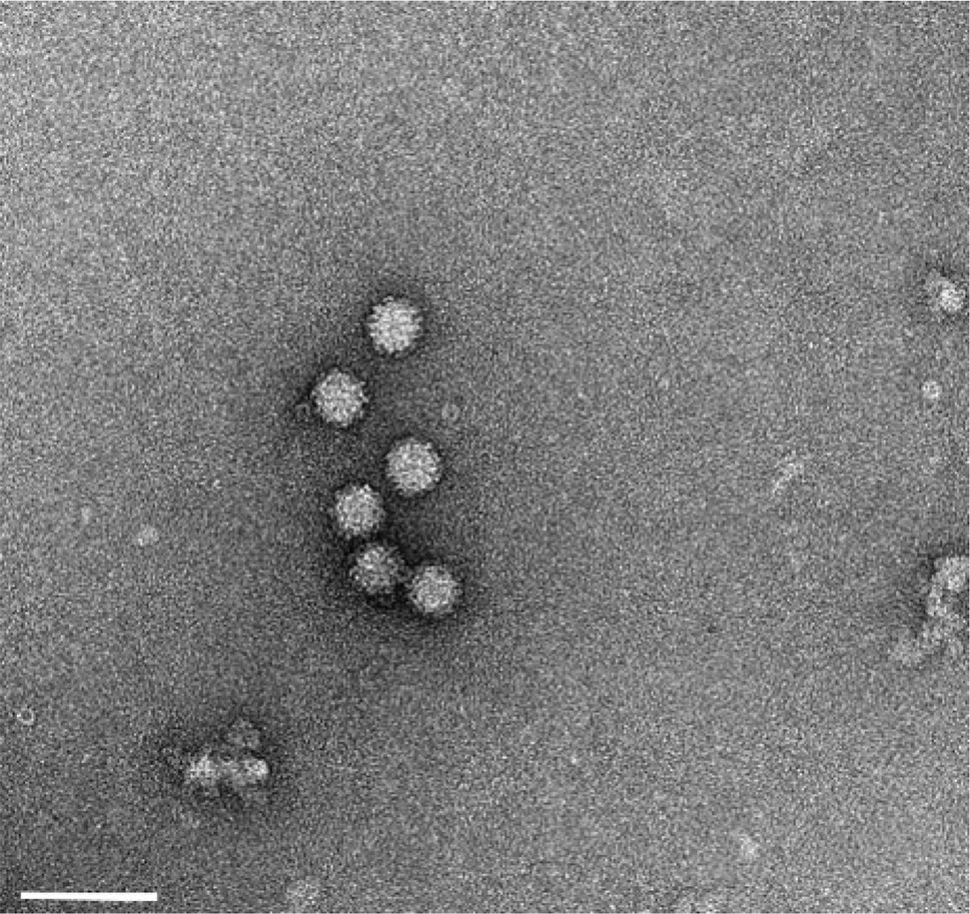
Transmission electron microscopy (TEM) imaging of Tulane virus in Tris–EDTA suspension (pH 7.5). Scale bar 100 nm

Tulane virus from the cell lysate was concentrated and purified using iodixanol gradient ultracentrifugation, resulting in a final concentration of 9.5 log_10_ PFU per ml. Initial characterization of the virus stock involved assessing its integrity through dispersity assessment and size distribution using DLS and TEM. Size distribution using DLS on a 1:10 dilution of the purified virus (approximate final concentration of 8.5 log_10_ PFU per ml) showed that the purified virus particles were primarily monodispersed, with diameters of 36 ± 6 nm, having minimal presence of larger or smaller particles and a polydispersity index of 0.32 (Fig. 1A, B). Although the presence of larger particles was noted, they did not constitute a significant portion of the suspension in terms of volume and quantity. As demonstrated in the representative TEM image (Fig. 2), the virus suspension was composed of predominantly monodispersed particles, confirming its purity.

### Infectivity Assays

**Fig. 3:**
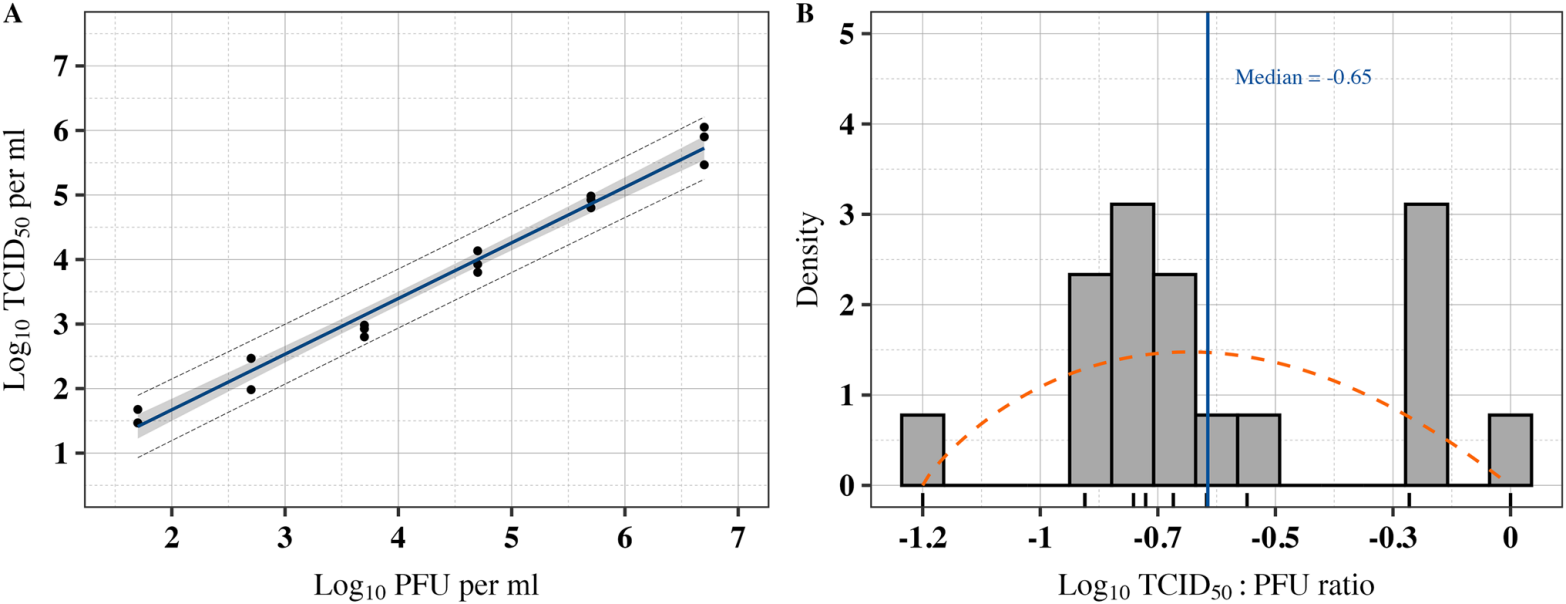
The association between TCID_50_ and plaque assay and predicted log_10_ TCID_50_:PFU ratio. **A** The linear relationship between log_10_ TCID_50_ and plaque assay for quantifying virus particles. The solid line shows the best fit, the gray shaded area represents the 95% confidence interval, and the long dash lines represent the 95% prediction intervals. At 0.7 log_10_ PFU/ml, all the replicates for the TCID_50_ assay fell below the detection limit of 1.47 log_10_ and thus were excluded from data analysis. **B** Comparison of empirical and fitted Beta distributions of log_10_ TCID_50_:PFU ratio. The histogram represents the empirical distribution of the ratio data (*n* = 18). The density plot of simulated data is depicted with the dashed line, calculated based on maximum goodness-of-fit estimation on scaled data. The solid vertical line marks the −0.65 bootstrapped median of the fitted distribution for the log_10_ TCID_50_:PFU ratio. The plot highlights the close alignment between the empirical data and the fitted Beta distribution, highlighting the central tendency and spread of the data.

The quantification of Tulane virus in the working stock using the TCID_50_ technique yielded a mean value of 5.81 ± 0.18 log_10_ TCID_50_/ml, compared with the 6.72 ± 0.10 log_10_ PFU/ml from the plaque assay. All virus suspensions at 0.7 log_10_ PFU/ml did not result in any CPE and were excluded from data analyses (limit of detection: 1.47 log_10_ TCID_50_/ml). The estimation of serially diluted virus stock using TCID_50_, relative to the plaque assay, was used to establish the best model fit. The prediction accuracy of the models, evaluated with RMSE, showed similar values for both the linear (0.198) and quadratic polynomial (0.175) model fits. However, the AICc, which penalizes models with additional parameters, indicated that the polynomial model had a lower AICc value by 1.09 units. Although the polynomial regression offered a marginally better fit, the linear regression model was selected for its parsimony (Fig. 3 and Table 2).

**Table 2:** Summary of regression model estimates for quantification of Tulane virus with RT-qPCR and infectivity assays (plaque assay and TCID_50_)

No	Sample	Linear model variables^a^ (predictor ~ response)	Coefficients [95% CI: lower, upper]	R^2^
1)	TCID_50_:PFU	log_10_ TCID_50_ ~ log_10_ PFU per ml	*β*_0_ = −0.05 [−0.33, 0.22] *β*_1_ = 0.86 [−0.80, 0.92]	0.981
2)	RT-qPCR calibration curve	Ct ~ log_10_ GC per reaction	*β*_0_ = 48.36 [47.90, 48.82] *β*_1_ = −3.81 [−3.89, −3.74]	0.995
3)	GC:PFU (with RNase)	log_10_ GC ~ log_10_ PFU per reaction	*β*_0_ = 3.84 [3.73, 3.95] *β*_1_ = 0.93 [0.89, 0.97]	0.991
4)	GC:PFU (without RNase)	log_10_ GC ~ log_10_ PFU per reaction	*β*_0_ = 4.07 [3.93, 4.20] *β*_1_ = 0.93 [0.88, 0.98]	0.987

^a^The coefficients represent the linear line formula, described as *y* = *β*_0_ + *β*_1_x

The average log_10_ TCID_50_:PFU was −0.63 ± 0.07 (or non-log_10_ transformed of 0.30 ± 0.06). The ANOVA revealed significant differences in log_10_ TCID_50_:PFU ratios across the dilutions (*p* = 0.001), suggesting that the dilution levels had significant effects on the ratios. Further post hoc analysis using Tukey’s HSD test showed that the highest dilution (1.7 log_10_ PFU/ml) yielded a significantly higher ratio, compared to the lower dilutions (4.7, 5.7, 3.7, and 6.7 log_10_ PFU/ml), with a mean ratio of 0.71 log_10_ TCID_50_/ml. However, no significant differences were found among the lower dilutions. To further assess the agreement between the predicted PFU values from the log_10_ TCID_50_:PFU ratio over the calculated log_10_ PFU/ml, the Bland–Altman analysis indicated a near-zero bias of 1 × 10^–15^ [95% CI: −0.58, 0.58] (*p* = 1). The limits of agreement (LOA) were between −2.3 and 2.3. Overall, the analysis suggests good agreement between the predicted and observed values, with no significant bias, though the wide limits of agreement indicate some variability in individual predictions. The Beta distribution parameters using the bootstrapped normalized log_10_ TCID_50_:PFU data resulted in ⍺ of 1.80 [95%CI: 0.97, 4.47], β of 2.02 [95%CI: 1.04, 5.31]. The distribution resulted in a median log_10_ TCID_50_:PFU of −0.65 [95%CI: −1.15, −0.16]. Figure 3 demonstrates the relationship between TCID_50_ across serial dilutions of Tulane virus and Beta distribution of log_10_ TCID_50_:PFU ratio.

The validation of the predictive power of the model was performed by a 70–30 split of data, representing train and test data, with five partitions. The RMSE values ranged from 0.25 to 1.60, with a median of 0.89, indicating that the model performed with varying accuracy across different partitions. The relatively high RMSE in some partitions, despite high R^2^ values of 1.0, could be due to the small data size used for model validation that warrants further investigation when applying the model to new or unseen data.

### Genome Copies Estimation

**Fig. 4:**
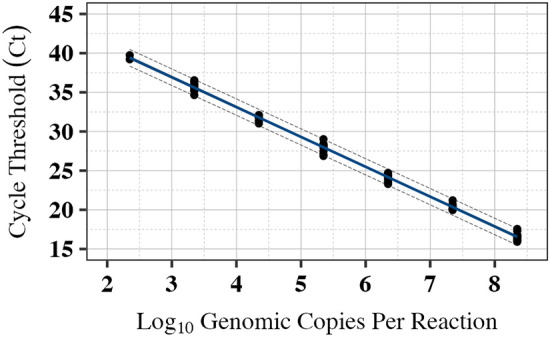
Tulane virus RT-qPCR calibration curve established by testing tenfold serial dilutions of in vitro RNA transcripts. The regression line was established by plotting the Ct values against the log_10_ genome copies of the transcripts per PCR reaction (after removing one reaction with Ct > 40 for the dilution with 2.35 log_10_ GC/PCR reaction. The solid line indicates the best fit, the gray shaded areas represent the 95% confidence intervals, and the long dash lines represent the 95% prediction intervals. The regression line estimates are provided in Table 2

A calibration curve generated by RT-qPCR testing of serially diluted Tulane virus transcripts was established to estimate GCs in samples (Fig. 4, Table 2). Although RT-qPCR was run for up to 45 cycles, data with Ct values greater than 40 were excluded from the analyses (which was only observed in one sample at 2.4 log_10_ GC/reaction). The removal of data with low RT-qPCR signal was based on the commonly observed phenomena in RT-qPCR on reduced amplification efficiency and assay sensitivity at Ct values higher than 40 (Trang et al., 2015). All dilutions showed positive PCR signals except for the highest dilution (2.4 log_10_ GC/PCR reaction), which resulted in 1/3, 1/3, and 0/3 positivity in each replicate. No amplification was observed for templates at 1.32 or lower log_10_ GC/PCR reaction, even after 45 cycles. Removal of the high Ct value sample increased model performance by 15.2 AICc value. To assess GC predictions for Ct of 16–40 using calibration curves without and with Ct > 40, the Bland–Altman analysis showed that, on average, the inclusion of high Ct values in the calibration curve may lead to 0.013 [95% CI: 0.007, 0.018] overestimation of log_10_ GC (*p* < 0.001). Most of the differences between the two predictions fell within the limits of agreement (LoA) of −0.026 to 0.051, indicating that both approaches produced relatively small variations with marginal impact on virus quantification. Assuming consistent amplification efficiency in RT-qPCR, using the same approach for predicting hypothetical Ct 40–50 overestimated virus for 0.060 [95% CI: 0.057, 0.062] log_10_ GC (*p* < 0.001) with LoA of between 0.043 and 0.075, indicating a marginal magnitude of virus prediction. In this study, selecting the model with a better fit (i.e., after removing Ct > 40) was employed to enhance the precision of the measurements. The estimated model slope of the calibration curve (Table 2) resulted in an amplification efficiency of 83.01% [95% CI: 80.75%, 85.09%] for the RT-qPCR reaction.

### RT-qPCR Detection of Tulane Virus with and without RNase Pretreatment

**Fig. 5:**
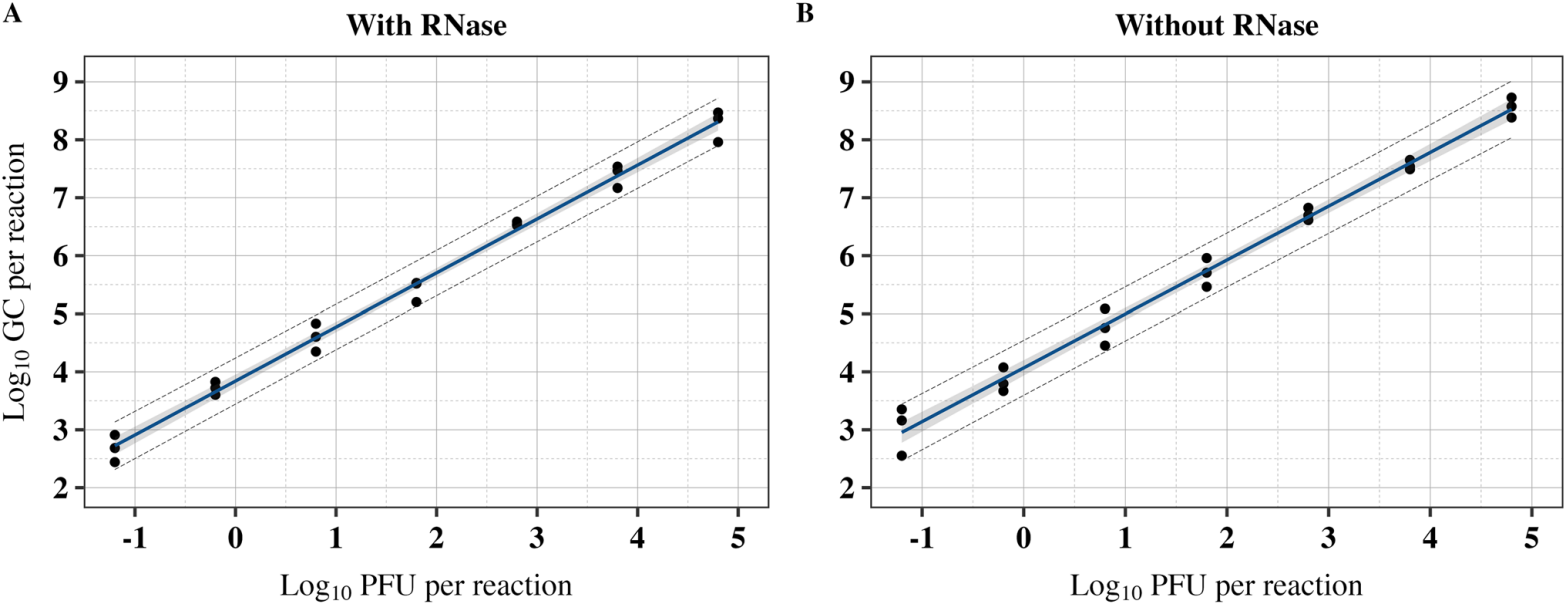
Linear relationship between log_10_ GC and log_10_ PFU per PCR reaction for Tulane virus with and without RNase pretreatment. The solid lines indicate the best fit, the gray shaded areas represent the 95% confidence intervals, and the long dash lines represent the 95% prediction intervals. The regression line estimates are provided in Table 2

Using the established calibration curve, serially diluted virus stock from 4.8 to −2.2 log_10_ PFU/reaction was subjected to RT-qPCR with and without RNase pretreatments. Using the calibration curve, the log_10_ GC/reactions were predicted (Fig. 5, Table 3). At dilutions equivalent to −2.2 log_10_ PFU/reaction, the signals showed high variability or no amplification for samples with and without RNase pretreatment. Consequently, these data points were excluded from data analyses. The associations between RT-qPCR and plaque assay were assessed using the regression models and Pearson’s product-moment correlation coefficients.

**Table 3:** Data summary of the plaque assay and RT-qPCR testing of serially diluted Tulane virus stock

Pretreatment	Log_10_ PFU per reaction^a^	Average Ct	Log_10_ GC per reaction	Log_10_ GC:PFU ratio^b^
With RNase	4.8	16.50 ± 0.53	8.34 ± 0.14	3.47 ± 0.16
	3.8	20.16 ± 0.75	7.38 ± 0.20	3.59 ± 0.11
	2.8	23.34 ± 0.12	6.53 ± 0.03	3.75 ± 0.02
	1.8	27.73 ± 0.72	5.39 ± 0.19	3.61 ± 0.11
	0.8	30.87 ± 0.92	4.57 ± 0.24	3.80 ± 0.14
	−0.2	34.23 ± 0.43	3.68 ± 0.11	3.92 ± 0.06
	−1.2	36.55 ± 0.90	2.64 ± 0.24	3.88 ± 0.14
Without RNase	4.8	15.67 ± 0.66	8.55 ± 0.17	3.76 ± 0.10
	3.8	19.51 ± 0.32	7.55 ± 0.08	3.76 ± 0.05
	2.8	22.77 ± 0.41	6.69 ± 0.11	3.91 ± 0.14
	1.8	26.60 ± 0.95	5.69 ± 0.25	3.91 ± 0.14
	0.8	30.22 ± 1.21	4.74 ± 0.32	3.96 ± 0.18
	−0.2	33.74 ± 0.80	3.81 ± 0.21	4.05 ± 0.12
	−1.2	36.55 ± 1.03	3.08 ± 0.27	4.22 ± 0.24

^a^PFU: Plaque forming units. The results represent the mean ± standard error of data obtained from three technical and two biological replicates. Genomic copies (GC) were measured by the calibration curve of the Tulane virus

^b^Pairwise t-test with Bonferroni adjustment did not indicate any significant difference between log_10_ GC:PFU ratios for with or without RNase pretreated samples (*p* > 0.05)

The RNase-pretreated and non-RNase-pretreated samples demonstrated strong Pearson’s product-moment correlation coefficient, with respective adjusted R^2^ of 0.996% and 0.994%, with no significant difference between the correlation coefficients (Fisher’s z = 0.529, *p* = 0.597). The linear regression models showed a strong fit, as presented in Table 2. Bland–Altman analyses of predicted log_10_ PFU/reaction from log_10_ GC/reaction showed that the predicted values in samples without RNase pretreatment were 0.223 log_10_ GC/reaction [95% CI: 0.222, 0.225] higher than samples with RNase pretreatment (*p* < 0.001). The low and narrow spread of LoA (ranging between 0.215 and 0.232) indicates a close similarity between virus detection with and without RNase pretreatments with a small deviation in the predictions.

**Fig. 6:**
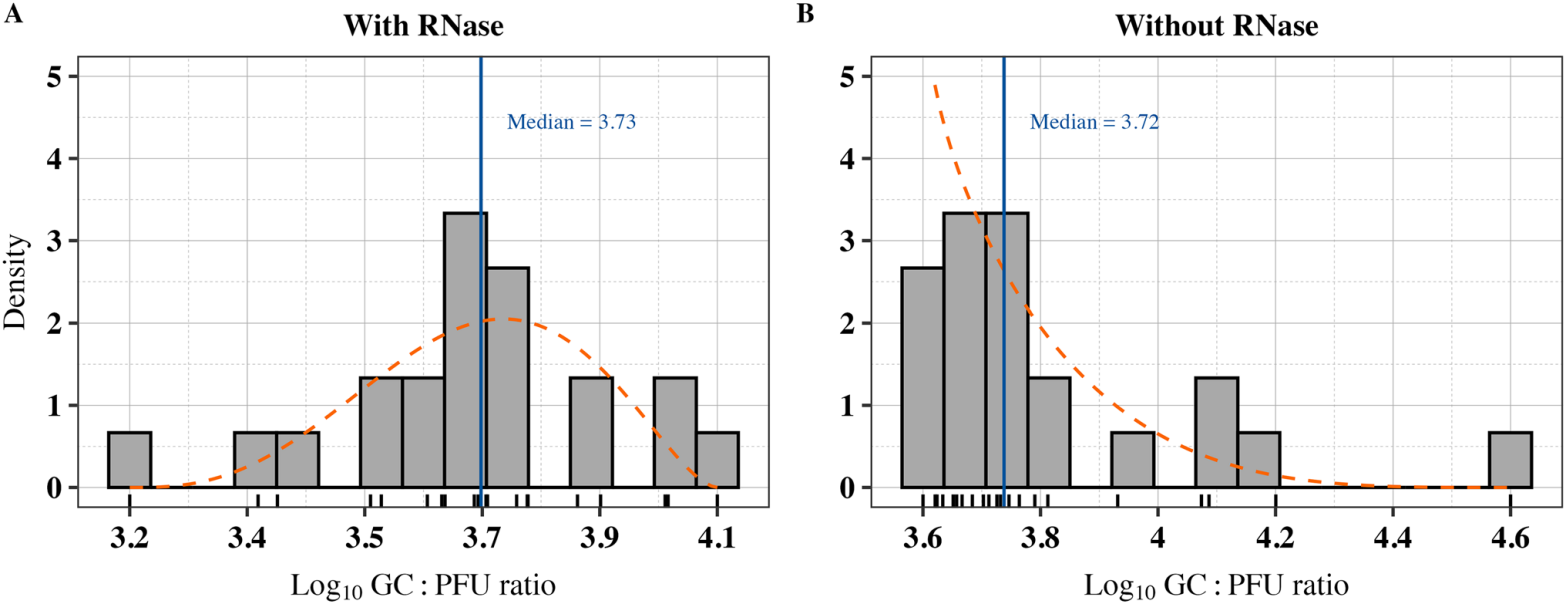
Comparison of empirical and fitted Beta distributions of log_10_ GC:PFU ratios. The histogram represents the empirical distribution of the ratio data (*n* = 21). The density plots are depicted with dashed lines, calculated based on maximum goodness-of-fit estimation on scaled data. The solid vertical lines mark the bootstrapped medians of the fitted distributions shown as log_10_ GC:PFU ratios. The plots highlight the close alignment between the empirical data and the fitted Beta distributions, highlighting the central tendency and spread of the data

The ratio between log_10_ GC to log_10_ PFU/reaction was calculated for each virus dilution (Table 3). The resulting ratios in logarithmic and non-logarithmic forms were 3.72 ± 0.07 and 5,864 ± 832 for those with RNase and 3.94 ± 0.07 and 10,525 ± 2,259 for those without RNase pretreated samples (*p* = 0.003). The two-way ANOVA revealed that while virus concentration is significantly impactful on the log_10_ GC:PFU ratios (*p* = 0.022), the impact of RNase pretreatment on the log_10_ GC:PFU ratios does not depend on the specific concentration of the virus. Further pairwise comparisons using Bonferroni adjustment show no significant pairwise differences in log_10_ GC:PFU across the concentrations of virus (*p* > 0.05).

Using the linear regression fits for predicting log_10_ PFU/reaction from log_10_ GC:PFU, the Bland–Altman analysis resulted in 1.17 [95% CI: 1.04, 1.29] unit higher predictions for samples without RNase pretreatment with LoA between 2.05 and 0.28. The prediction validation through the train and test set was performed separately with and without RNase pretreatments. As shown in Table 4, differences were observed between the models with and without RNase pretreatments. Overall, the model for samples with RNase pretreatment provided a better accuracy (lower RMSE), but the model without RNase pretreatment tends to explain more variance (higher R^2^), particularly in the higher quartile.

**Table 4:** Model validation summary for samples with and without RNase pretreatments

	1st percentile	Median	Mean	75th percentile
With RNase				
RMSE	0.54	1.69	1.51	1.90
R^2^	0.07	0.13	0.32	0.45
Without RNase				
RMSE	1.68	1.75	1.75	1.92
R^2^	0.09	0.24	0.38	0.91

To better capture the uncertainty in the relationship between RT-qPCR and plaque assay data, the normalized log_10_ GC ratios, with and without RNase pretreatments, were fitted to Beta distributions via the maximum goodness-of-fit method (Fig. 6). Bootstrap simulation to estimate uncertainty in distribution parameters resulted in median values of *α* = 3.97 [95% CI: 2.04, 9.45] and *β* = 2.70 [1.43, 6.49] for samples with RNase pretreatment data and *α* = 0.92 [95% CI: 0.54, 2.01] and *β* = 4.62 [2.25, 11.88] for samples without RNase pretreatment. Despite significant differences between the density plots as determined by the Kolmogorov–Smirnov test (*D* = 0.8, *p* < 0.001) and the Wilcoxon rank sum test (*W* = 10^6^, *p* < 0.001), the median simulated log_10_ GC ratios for RNase pretreated data and unpretreated data showed similar values of 3.73 log_10_ [95% CI: 3.39, 4.02] and 3.72 log_10_ [95% CI: 3.59, 4.10], respectively.

## Discussion

Human norovirus is the primary cause of acute gastrointestinal disease worldwide and is often associated with the consumption of contaminated ready-to-eat food products (FAO/WHO, 2023; US CDC, 2024). Despite advancements in HuNoV detection, the lack of a reliable cell culture-based technique and the reliance on RT-qPCR for monitoring high-risk foods continue to pose significant challenges (ISO, 2017; US FDA, 2024; Vinjé, 2015). Given the low prevalence and concentration of HuNoV in high-risk foods, it is crucial to assess the accuracy and uncertainty of nucleic acid-based HuNoV detection techniques to enhance microbial risk assessment (Pouillot et al., 2022; Steele et al., 2022). This is particularly important when extrapolating the RT-qPCR results to assess infectivity. Existing methods to quantify infectious virus particles (including the plaque assay and TCID_50_) in cultivable HuNoV surrogates require further investigation of their association with RT-qPCR.

To address this knowledge gap, we used the Tulane virus as a reliable surrogate for HuNoV in inactivation studies (Cromeans et al., 2014). Plaque assay and TCID_50_ were used to measure infectivity, and their relationship was assessed to predict Tulane virus titer. Additionally, virus samples were tested with RT-qPCR before and after pretreatment with RNase to evaluate the efficacy of RNase on targeting intact (presumptively infectious) virus particles. The virus stock was purified with a gradient ultracentrifugation technique, which proved to be effective for purifying and concentrating virus particles. Iodixanol, used for creating gradient layers for ultracentrifuge, is less viscous than cesium chloride (CsCl) and sucrose, and its isosmotic properties help preserve the integrity and functionality of virus particles (Segura et al., 2011). Initial characterization of the virus stock with DLS and TEM showed that the majority of suspended viruses consisted of dispersed particles with a mean diameter of 36 nm, which is in line with previous studies (Farkas et al., 2008; Yu et al., 2013). Sonication was also perfumed on virus suspensions to reduce aggregates without impacting viral infectivity (Barnes et al., 2021; Jones et al., 2009; Pinto et al., 2010).

The limits of detection for the plaque assay and TCID_50_ were 0.69 log_10_ PFU/ml and 1.47 log_10_ TCID_50_/ml, respectively. The values of TCID_50_ and plaque assay are not easily comparable because the two methods assess different aspects of viral infectivity. The plaque assay quantifies virus particles, while TCID_50_ estimates the number of viruses needed to infect 50% of the host cells. Therefore, the outcome of these assays for virus quantification vary (Lei et al., 2021; Smither et al., 2013). The established relationship between plaque assay and TCID_50_ in our study was consistent with the findings of Shan et al. (2016) that reported a positive linear relationship between these two assays, with an R-value of 0.85 from multiple regression. They also found the TCID_50_ method to be more sensitive than the plaque assay for measuring Tulane virus infectivity based on their ratio, where, on average, the TCID_50_ assay produced viral counts approximately 6.69 times higher than those from the plaque assay (Shan et al., 2016). On the other hand, our results showed that the mean ratio of the log_10_ TCID_50_:PFU was −0.63 ± 0.07 (or non-log_10_ transformed of 0.30 ± 0.60), with a median of −0.67 log_10_ (or non-log_10_ transformed of 0.21). This difference in ratios can be explained by the fact that TCID_50_ and plaque assay for assessment of virus infectivity can be affected by the host, MOI, serum concentration, and other experimental conditions (Arifin et al., 2011; Hotter et al., 2024; Rimmelzwaan et al., 1998).

For establishing the RT-qPCR calibration curve, we excluded one reaction with a Ct value above 40 to ensure accuracy in the subsequent GC:PFU ratio measurements. However, this point could be reintroduced to account for greater uncertainty in measurement. Variability in measurements at low GC concentrations (usually when the Ct values are higher than 40) has been shown through the observation that confidence intervals (measurement of uncertainty), are significantly wider when amplification starts from a small number of template copies compared to when it begins with a larger initial copy number (Bustin et al., 2009; Trang et al., 2015). To address the limitation of RT-qPCR in distinguishing infectious from non-infectious virus particles, we evaluated the predictability of RT-qPCR with and without pretreatments with RNase. In our study, no significant difference was observed in the log_10_ GC between samples with and without RNase pretreatments, which may be due to the separation of free and degraded RNA by ultracentrifugation. It has been reported that RNase pretreatment of murine norovirus can reduce RT-qPCR signal by up to around 3 log_10_ PCR units (Ronnqvist et al., 2014). Despite RNase pretreatment, there were differences between RT-qPCR results and infectivity assays, particularly at low doses of UV light. Low doses can cause chemical dimerization and cross-linking of the viral genomic material. While these viruses were not infectious, amplification of their genomes still occurred, highlighting a limitation of RNase pretreatment in distinguishing infectious viral particles (Ronnqvist et al., 2014). Additionally, heating murine norovirus and HuNoV (70 °C and 85 °C for 2 min) resulted in no significant difference in samples with and without RNase pretreatments. A possible explanation is that the RNA inside the damaged capsids may have been degraded by the innate RNase present in the tissue culture lysate, and the addition of RNase One did not lead to any notable changes (Li et al., 2012). Exposure of HuNoV to neutral electrolyzed water (containing 50 to 200 ppm free available chlorine for 1 min) revealed no significant differences between RNase-pretreated and unpretreated samples (Moorman et al., 2017). Although these pretreatments rendered the viruses non-infectious (through capsid denaturation), the viral genomes remained amplifiable, suggesting the inability of RNase to identify infectious viral particles in such scenarios (Li et al., 2012). These findings underscore that the use of RNase pretreatment should be based on the specific goals of virus testing and the anticipated effects on viral integrity, including the genome, capsid, or both. Further investigation is needed to evaluate the variable nature of the GC:PFU ratio and to determine potential shifts in the correlation between infectivity assays and RNase RT-qPCR results following virus exposure to different treatments, including heat, UV radiation, and chemical agents.

For predicting virus infectivity based on PCR results, log_10_ GC and log_10_ PFU/reaction on Tulane virus showed a strong positive correlation for samples with and without RNase pretreatments. This outcome is contrary to previous report, which did not find a strong correlation between GC and PFUs in Tulane virus (Shan et al., 2016). A possible explanation for this discrepancy is that they used different stocks at varying concentrations to account for variability across different virus passages, whereas we employed serially diluted virus suspensions to account for testing of virus detection at different titers. Similar to our findings, regression analysis showed a high association between RT-qPCR and the plaque assay on the influenza A virus (adjusted R^2^ > 0.90) and that the infectious virus particles could be predicted without direct infectivity assays (Nakaya et al., 2020). As a limitation, the plaque assay was unable to detect infectivity at low concentrations beyond 1.7 log_10_ PFU/ml. Given the highly infectious nature of HuNoV, it is crucial to detect the virus even in low concentrations (Teunis et al., 2008). Blind passage has been recognized as an alternative qualitative method for enhancing the diagnostic yield of viruses; however, the outcomes may not be quantitative (Kumar et al., 2016; Weinberg et al., 1996).

Compared to the plaque assay, RT-qPCR quantified Tulane virus particles an average of 5,864 times higher with RNase and 10,525 times higher without RNase pretreatment. Simulation of these observational data through Beta distributions resulted in a closely similar median GC:PFU ratio of ~ 5,250 for both RNase-pretreated and unpretreated samples. The relatively high GC:PFU ratio highlights a common disparity, with PCR values being considerably higher than those obtained from the plaque assay. In another study, murine norovirus of vacuum-packaged raw pork chops has shown a GC:PFU ratio of 0.21 log_10_ (or non-log_10_ transformed of 1.621) which is notably lower than our results (Brandsma et al., 2012). The observed high GC:PFU ratio, even after RNase pretreatment, may be due to the intact viruses being present in the sample without the chance of causing infection primarily due to the defective genome or capsid (Veugen et al., 2024). RT-qPCR continues to detect signals from intact particles that may not be infectious.

The presence of inhibitors in the virus-associated matrix may also affect the observed GC:PFU by underestimating the GC numbers in samples due to the reduced amplification efficacy of RT-qPCR (Knight et al., 2013). In a study using droplet digital RT-qPCR (ddRT-PCR) and plaque assay to quantify murine norovirus in oyster digestive tissue homogenates, researchers observed a GC:PFU ratio of 28 ± 1.0, which is lower than what we found for Tulane virus (Plante et al., 2021). Although digital RT-qPCR is generally less sensitive to inhibitors than RT-qPCR, amplification of target genes can still be affected, potentially impacting the accuracy of ddRT-PCR and, consequently, the calculated GC:PFU ratio (Dingle et al., 2013; Hall Sedlak & Jerome, 2014). The type of host cells and passage number are found to impact the observed GC:PFU ratios. For example, these conditions in murine norovirus led to GC:PFU ratios ranging from approximately 1:190 to 1:19,000. Viruses passaged six times in HeLa cells exhibited enhanced replication compared to the parental virus. However, in BV-2 cells, viruses showed decreased replication. Additionally, viruses with increased passage numbers had significantly higher RNA:PFU ratios than initial passages, indicating a reduced replication efficiency (Budicini et al., 2024). Some studies have noted that discrepancies in GC:PFU ratios could be attributed to virus aggregation, where virus particles clump together, potentially forming a single plaque in the infectivity assay (Nakaya et al., 2020). In our study, ultracentrifugation was able to purify the virus stock and yield monodispersed virus particles, as shown by DLS and TEM data.

In conclusion, this study established a foundation for assessing the comparative predictive powers of nucleic acid-based and culture-based techniques for norovirus quantification. Due to the unavailability of a culture-based method for absolute quantification of HuNoV, the use of the Tulane virus as one of the most suitable surrogates served as a close approximation for evaluating the relationships across various detection techniques. Under our experimental conditions, the developed linear regressions effectively described these relationships with a high level of robustness. The application of RNase validated previous research by providing an approach to reduce RT-qPCR signals from damaged virus particles or exposed viral RNA, making RT-qPCR results more comparable to infectivity assay. RNase pretreatment has limitations in distinguishing between infectious and non-infectious in intact virus particles. More research is needed to assess its comparative applicability with alternative pretreatment methods, such as PMA and porcine gastric mucin, which have shown promising potential and merit further investigation in this context. Given that outcomes of these techniques can be influenced by various intrinsic and extrinsic factors, further research is needed to address the uncertainties and variabilities in viral infectivity assessments to improve the accuracy of microbial risk assessment frameworks and advance our strategies for the surveillance, prevention, and mitigation of HuNoV in high-risk foods.

## Supplementary Information

Below is the link to the electronic supplementary material.

Supplementary file1 (ZIP 817 KB)

## Data Availability

The associated data files and R codes are provided in the supplementary materials for reproducibility.
