# Supplementary material for: Navigating Uncertainties in RT-qPCR and Infectivity Assessment of Norovirus
Source: Food Environ Virol. 2025 Mar 8;17(1):22. doi: 10.1007/s12560-024-09632-0 (PMC11890344; doi:10.1007/s12560-024-09632-0)
Supplement: Supplementary file 1 — Supplementary file1 (ZIP 817 KB) [file 12560_2024_9632_MOESM1_ESM.zip › Data and R codes R1/GC2Inf article codes_NM122524.pdf]

R codes for GC2Inf article

N. Montazeri and R. Mirmahdi

2024-12-25

Table of contents

1. Global settings 2
2. TCID50 vs. PFU 4
2.1 Data entry and initial cleanup 4
2.2 Finding the best regression model fit 4
2.3 Plotting 7
2.3.1 Predictions from data seq for plotting 7
2.3.2 Plot 8
3. TCID50:PFU ratio 10
3.1 Predicting TCID50:PFU over PFU (obs) 14
3.2 Agreement analyses and visualizations 15
3.3 Beta distribution 17
3.4 Model validation through train data (using original data) 20
4. RT-qPCR calibration curve 23
4.1 Data entry and initial cleanup 23
4.2 Regression and outlier assessment 24
4.2.1 Comparing model fits (w/ or w/o Ct>40) 24
4.3 Ct 16-40 predictions of GC w/ and w/o removing Ct>40 27
4.3.1 Reverse models for predictions 27
4.3.2 Prediction calculations 28
4.3.3 Models agreement analyses and visualizations 29
4.4 Ct 40-50 predictions of GC w/ and w/o removing Ct>40 31
4.4.1 Prediction calculations 31
4.4.2 Models agreement analyses and visualizations 32
4.5 Plot of RT-qPCR calibration curve 34
4.5.1 Generate predictions and intervals 34
4.5.2 Plot 35
5. GC over PFU 37

5.1 Data entry and initial cleanup 37
5.2 Calculate GC and averaging tech reps 38
5.2.1 GC over PFU Regression W/ and W/O RNase 39
5.3 Correlation b/w GC and PFU 43
5.4 GC over PFU Predictions 44
5.4.1 Models agreement analyses and visualizations 45
5.5 Plot of GC over PFU 48
6. GC:PFU ratio 51
6.1 GC:PFU for w/ vs. w/o RNase 51
6.2 Beta distributions 54
6.3 PFU over GC:PFU ratio predictive power 61
6.3.1 PFU ~ GC:PFU 61
6.3.2 PFU ~ log GC:PFU predictions 63
6.3.3 Models agreement analyses and visualizations 64
6.3.4 Model validation through train data (using original data) 66

1. Global settings

```
# Packages
require(readxl)
require(writexl)
require(tidyr)
require(dplyr)
require(AICcmodavg)
require(agricolae)
require(caret)
require(blandr)
require(splines)
require(ggplot2)
require(ggpubr)
require(extrafont)
require(cocor)
require(fitdistrplus)

# update.packages(ask = FALSE)
# font_import(prompt = FALSE) # import system fonts if needed.

# Set seed
set.seed(7910)
```

```
# Limit the number of significant digits to 3
options(digits=3)

## Turn in environments/packages/versions/etc ####
sessioninfo::session_info (pkgs = c("attached"))
```

```
─ Session info ───────────────────────────────────────────────
 setting  value
 version  R version 4.4.2 (2024-10-31)
 os       macOS Sequoia 15.1.1
 system   x86_64, darwin20
 ui       X11
 language (EN)
 collate  en_US.UTF-8
 ctype    en_US.UTF-8
 tz       America/New_York
 date     2024-12-25
 pandoc   3.2 @
/Applications/RStudio.app/Contents/Resources/app/quarto/bin/tools/x86_64/
(via rmarkdown)

─ Packages ───────────────────────────────────────────────────
 package      * version date (UTC) lib source
 agricolae    * 1.3-7   2023-10-22 [1] CRAN (R 4.4.0)
 AICcmodavg   * 2.3-3   2023-11-16 [1] CRAN (R 4.4.0)
 blandr       * 0.6.0   2024-06-09 [1] CRAN (R 4.4.0)
 caret        * 7.0-1   2024-12-10 [1] CRAN (R 4.4.1)
 cocor        * 1.1-4   2022-06-28 [1] CRAN (R 4.4.0)
 dplyr        * 1.1.4   2023-11-17 [1] CRAN (R 4.4.0)
 extrafont    * 0.19    2023-01-18 [1] CRAN (R 4.4.0)
 fitdistrplus * 1.2-1   2024-07-12 [1] CRAN (R 4.4.0)
 ggplot2      * 3.5.1   2024-04-23 [1] CRAN (R 4.4.0)
 ggpubr       * 0.6.0   2023-02-10 [1] CRAN (R 4.4.0)
 lattice      * 0.22-6  2024-03-20 [1] CRAN (R 4.4.2)
 MASS         * 7.3-61  2024-06-13 [1] CRAN (R 4.4.2)
 readxl       * 1.4.3   2023-07-06 [1] CRAN (R 4.4.0)
 survival     * 3.8-3   2024-12-17 [1] CRAN (R 4.4.1)
 tidyr        * 1.3.1   2024-01-24 [1] CRAN (R 4.4.0)
 writexl      * 1.5.1   2024-10-04 [1] CRAN (R 4.4.1)

 [1] /Library/Frameworks/R.framework/Versions/4.4-x86_64/Resources/library

──────────────────────────────────────────────────────────────
─
```

2. TCID50 vs. PFU

2.1 Data entry and initial cleanup

```
## TCID50: read original data ####
dat1 <- as.data.frame(read_excel("TCID50_plaque assay.xlsx"))
dat1$logTCID50ml <- as.numeric(dat1$logTCID50ml)
dat1 <- dat1[dat1$dil!= -6,] # At -5, the TCID50 already reached the LOD of
1.468 for 2 out of 3 replicates and no CPE was obsevred for -6 dilution.

# TCID50:PFU ratio
dat1$logtcid2pfu <- dat1$logTCID50ml-dat1$logpfuml
dat1$tcid2pfu <- (10^dat1$logTCID50ml)/(10^dat1$logpfuml)

# mean and std error of stock
##TCID50
mean(dat1$logTCID50ml[dat1$dil == 0], na.rm = TRUE)
```

```
[1] 5.81
```

```
sd(dat1$logTCID50ml[dat1$dil == 0], na.rm = TRUE)/sqrt(3)
```

```
[1] 0.175
```

```
str(dat1)
```

```
'data.frame':   18 obs. of  6 variables:
 $ dil        : num  0 0 0 -1 -1 -1 -2 -2 -2 -3 ...
 $ rep        : num  1 2 3 1 2 3 1 2 3 1 ...
 $ logpfuml   : num  6.7 6.7 6.7 5.7 5.7 5.7 4.7 4.7 4.7 3.7 ...
 $ logTCID50ml: num  5.47 5.9 6.05 4.98 4.8 ...
 $ logtcid2pfu: num  -1.232 -0.799 -0.649 -0.717 -0.899 ...
 $ tcid2pfu   : num  0.0586 0.1589 0.2244 0.1918 0.1262 ...
```

```
saveRDS(dat1, "TCID50_plaque assay.RDS")
```

2.2 Finding the best regression model fit

The data were processed to exclude the last dilution (-5 dilution) due to the fact that, at this point, the TCID50 values had already reached the limit of detection (LOD) of 1.468 in two out of three replicates.

```
# Linear model
dat1 <- readRDS("TCID50_plaque assay.RDS")

model_l <- lm(logTCID50ml ~ logpfuml, data = dat1)
summary(model_l)
```

```

Call:
lm(formula = logTCID50ml ~ logpfuml, data = dat1)
```

```
Residuals:
    Min      1Q  Median      3Q     Max 
-0.3361 -0.1876  0.0556  0.1659  0.3263 

Coefficients:
            Estimate Std. Error t value Pr(>|t|)    
(Intercept)  -0.0543     0.1315   -0.41     0.69    
logpfuml      0.8625     0.0290   29.75    2e-15 ***
---
Signif. codes:  0 '***' 0.001 '**' 0.01 '*' 0.05 '.' 0.1 ' ' 1

Residual standard error: 0.21 on 16 degrees of freedom
Multiple R-squared:  0.982, Adjusted R-squared:  0.981 
F-statistic:  885 on 1 and 16 DF,  p-value: 1.96e-15
```

```
# # Model diagnosis
opar <- par(mfrow = c(2,2), oma = c(0, 0, 1.1, 0))
plot(model_l, las = 1); par(opar)
```

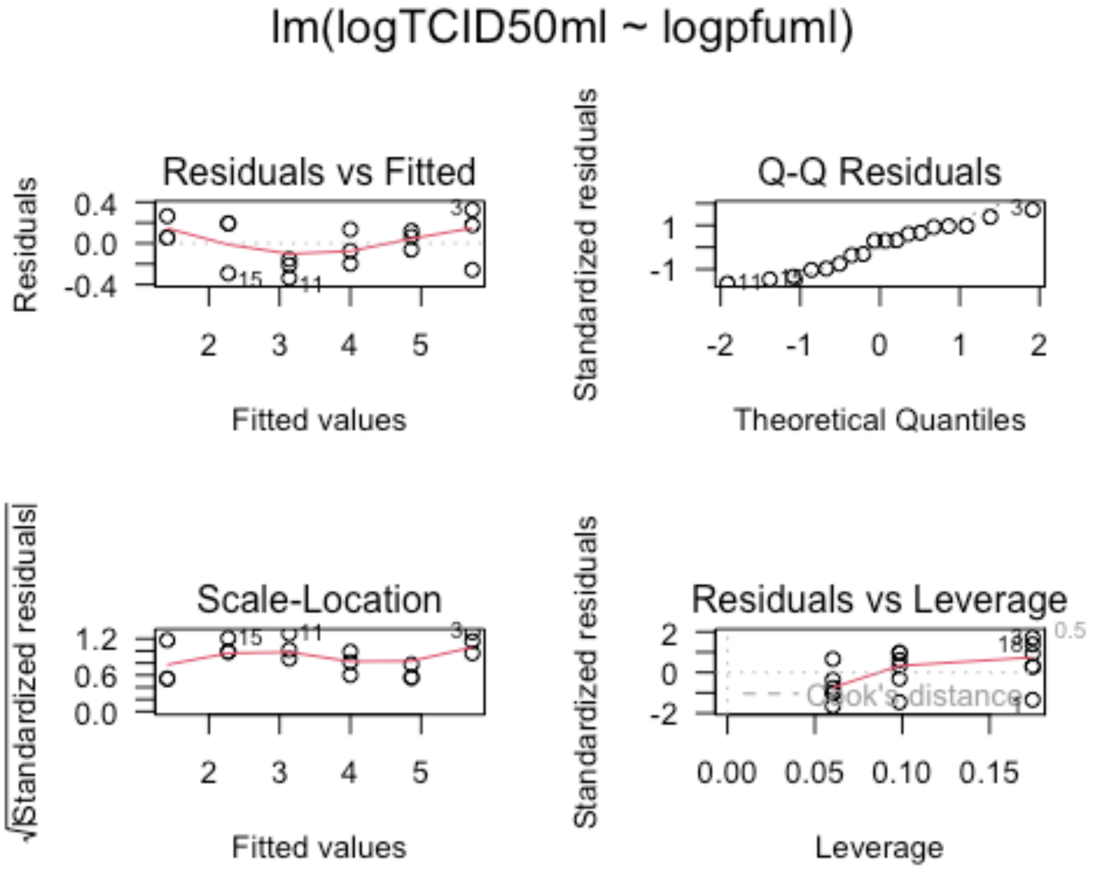


```
# Polynomial model
model_p <- lm(logTCID50ml ~ logpfuml + I(logpfuml^2), dat1)
summary(model_p)
```

```
Call:
lm(formula = logTCID50ml ~ logpfuml + I(logpfuml^2), data = dat1)

Residuals:
    Min      1Q  Median      3Q     Max 
-0.3809 -0.0917 -0.0055  0.1441  0.2338 

Coefficients:
              Estimate Std. Error t value Pr(>|t|)   
(Intercept)     0.4928     0.2925    1.68   0.1127   
logpfuml        0.5504     0.1545    3.56   0.0028 **
I(logpfuml^2)   0.0372     0.0181    2.05   0.0582 . 
---
Signif. codes:  0 '***' 0.001 '**' 0.01 '*' 0.05 '.' 0.1 ' ' 1

Residual standard error: 0.192 on 15 degrees of freedom
Multiple R-squared:  0.986, Adjusted R-squared:  0.984 
F-statistic:  533 on 2 and 15 DF,  p-value: 1.16e-14
```

```r
# # Model diagnosis
opar <- par(mfrow = c(2,2), oma = c(0, 0, 1.1, 0))
plot(model_p, las = 1); par(opar)
```

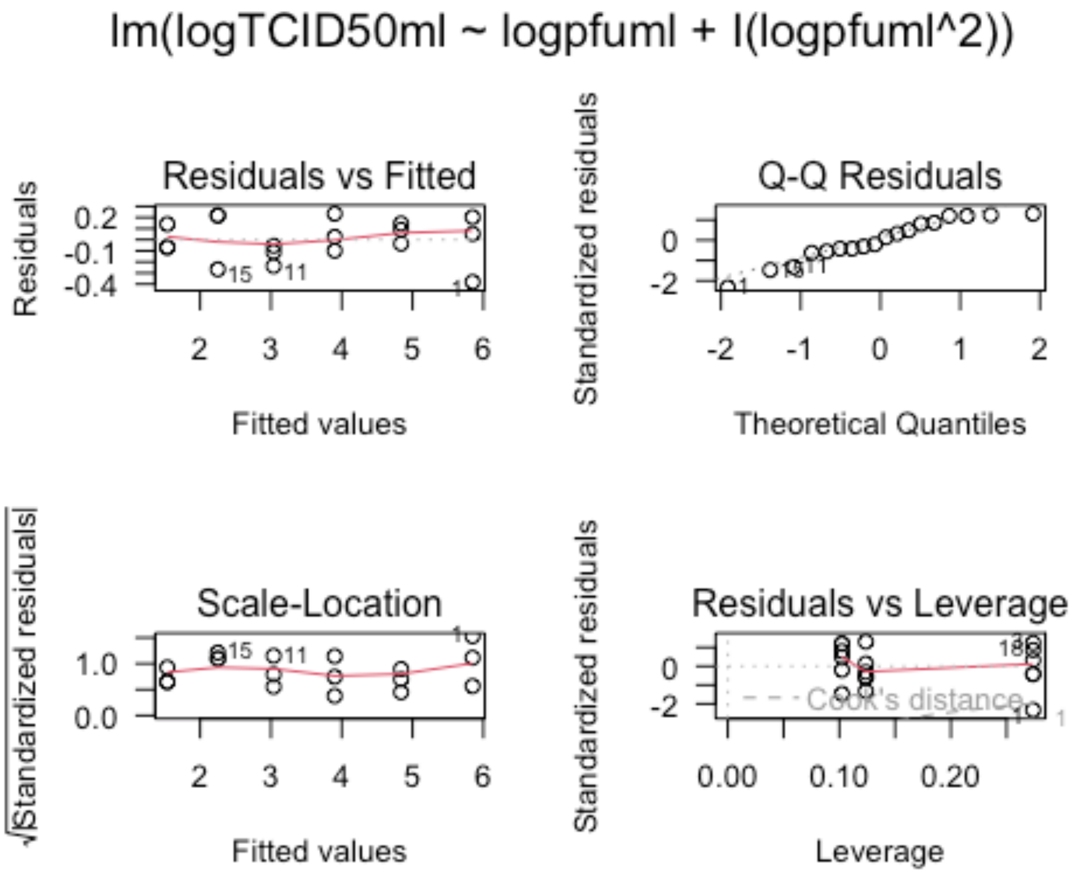

```
## Model Comparisons
## RMSE
sqrt(mean(model_l$residuals^2))
```

```
[1] 0.198
```

```
sqrt(mean(model_p$residuals^2))
```

```
[1] 0.175
```

```
## AICc comparison
AICcmodavg::aictab (list (model_l=model_l, model_p=model_p),
                    second.ord = TRUE)
```

```
Model selection based on AICc:

        K  AICc Delta_AICc AICcWt Cum.Wt   LL
model_p 4 -0.58       0.00   0.63   0.63 5.83
model_l 3  0.51       1.09   0.37   1.00 3.60
```

```
# RMASE and model estimates of the best model fit
preferred_fit = model_l
sqrt(mean(preferred_fit$residuals^2))
```

```
[1] 0.198
```

```
coef(preferred_fit)
```

```
(Intercept)    logpfuml
    -0.0543      0.8625
```

```
confint(preferred_fit)
```

```
             2.5 % 97.5 %
(Intercept) -0.333  0.224
logpfuml     0.801  0.924
```

```
rm(model_l, model_p, preferred_fit)
```

2.3 Plotting

2.3.1 Predictions from data seq for plotting

```
# read data and model fits
dat1 <- readRDS("TCID50_plaque assay.RDS")

data_seq <- data.frame(logpfuml=seq(min(dat1$logpfuml), max(dat1$logpfuml),
length.out = 50))

# selected model
model_l <- lm(logTCID50ml ~ logpfuml, data = dat1)
summary(model_l)
```

```
Call:
lm(formula = logTCID50ml ~ logpfuml, data = dat1)

Residuals:
    Min      1Q  Median      3Q     Max
-0.3361 -0.1876  0.0556  0.1659  0.3263

Coefficients:
            Estimate Std. Error t value Pr(>|t|)
(Intercept)  -0.0543     0.1315   -0.41     0.69
logpfuml      0.8625     0.0290   29.75    2e-15 ***
---
Signif. codes:  0 '***' 0.001 '**' 0.01 '*' 0.05 '.' 0.1 ' ' 1

Residual standard error: 0.21 on 16 degrees of freedom
Multiple R-squared:  0.982, Adjusted R-squared:  0.981
F-statistic:  885 on 1 and 16 DF,  p-value: 1.96e-15
```

```
# Predictions
## conditional means
ci_fit1 <- predict(model_l,
                   newdata = data_seq,
                   interval = "confidence", level = 0.95) |>
 as.data.frame() |>
 rename(ci_lwr = lwr,
        ci_upr = upr,
        ci_fit = fit)

## Predicted values
pred_fit1 <- predict(model_l,
                     newdata = data_seq,
                     interval = "prediction", level = 0.95) |>
 as.data.frame() |>
 rename(pred_lwr = lwr,
        pred_upr = upr,
        pred_fit = fit)

## Consolidate Predicted values and conditional means
new_data <- cbind(logpfuml=data_seq$logpfuml, pred_fit1, ci_fit1)

saveRDS(new_data, "pred_tcid50_from_pfu_data_seq.RDS")

rm(data_seq, model_l, dat1, pred_fit1,ci_fit1, new_data)
```

2.3.2 Plot

```
#| eval: true
#| echo: true
#| output: true
```

```
dat1 <- readRDS("TCID50_plaque assay.RDS")
dat2 <- readRDS("pred_tcid50_from_pfu_data_seq.RDS")

p1 <- ggplot () +
 geom_point(data=dat1,
            aes(x=logpfuml, y=logTCID50ml),
            color = "black", shape = 20, size=1.2) +
 stat_smooth (data=dat1,
              aes(x=logpfuml, y=logTCID50ml),
              color = c("#00529b"), linewidth=0.5, method="lm", se = FALSE,
              formula = y~x) +
 geom_ribbon(data = dat2,
             aes(x= logpfuml, ymin = ci_lwr, ymax = ci_upr),
             alpha = 0.2, fill = "gray10") +
  geom_line(data = dat2,
            aes(x = logpfuml, y = pred_lwr),
            linetype = "longdash", color = "gray15", linewidth=0.1) +
  geom_line(data = dat2,
            aes(x = logpfuml, y = pred_upr),
            linetype = "longdash", color = "gray15", linewidth=0.1) +
  scale_x_continuous (breaks = c (2, 3, 4, 5, 6, 7),
                      limits = c(1.5,7)) +
 scale_y_continuous (breaks = c (1,2,3,4,5,6,7,8),
                      limits = c(0.5, 7.5)) +
 labs (x = bquote (Log[10]~PFU~per~ml), size = 3,
       y= bquote (Log[10]~TCID[50]~per~ml), size = 3) +
 theme_bw (base_family="") +
 theme(
  text = element_text(family = "Times"),
  legend.position = "bottom",
  legend.direction="horizontal",
  legend.text=element_text(size = 6, face='bold'),
  legend.title=element_text (size = 6,face='bold'),
  plot.title = element_text(color="black", size=10, face = "bold", hjust =
0.5),
  axis.text.x=element_text(size = 10, color="black", face="bold", hjust =
0.5, vjust = 0.5),
  axis.text.y=element_text (size = 10, color="black", face="bold"),
  axis.title.x = element_text (size = 10, vjust = -1),
  axis.title.y = element_text (size = 10, vjust = 2),
  panel.grid.major = element_line (color="darkgray", linewidth=0.1,
linetype="solid"),
  panel.grid.minor = element_line (color="gray", linewidth=0.1,
linetype="dashed"),
  panel.background = element_rect (fill =  "transparent"),
  legend.background = element_rect (fill = "transparent", color =
"transparent"),
  plot.background = element_rect (fill = "transparent", color =
"transparent")) +
```

```r
 guides(color = "none")

# this plot will be combined with the TCID50:PFU Beta distribution plot
```

3. TCID50:PFU ratio

```r
# reading data
dat1 <- readRDS("TCID50_plaque assay.RDS")

# Average and std error
mean(dat1$logtcid2pfu)

[1] -0.632

sd(dat1$logtcid2pfu)/sqrt(length(dat1$logtcid2pfu))

[1] 0.0745

mean(dat1$tcid2pfu)

[1] 0.303

sd(dat1$tcid2pfu)/sqrt(length(dat1$tcid2pfu))

[1] 0.0574

# Whether the log tcid50:pfu is different across dilutions
dat1$logpfuml <- as.factor(dat1$logpfuml)
aov1 <- aov(tcid2pfu ~ logpfuml, data = dat1);summary(aov1)

            Df Sum Sq Mean Sq F value Pr(>F)
logpfuml     5  0.787  0.1574    8.59 0.0012 **
Residuals   12  0.220  0.0183
---
Signif. codes:  0 '***' 0.001 '**' 0.01 '*' 0.05 '.' 0.1 ' ' 1

# Pairwise comparison by separating w/ and w/o rnase. pfu_rxn has to be a
factor
pairwise.t.test(dat1$tcid2pfu, dat1$logpfuml,
                p.adjust.method = "bonf",
                alternative = c("two.sided"))


    Pairwise comparisons using t tests with pooled SD

data:  dat1$tcid2pfu and dat1$logpfuml

    1.7   2.7   3.7   4.7   5.7
2.7 0.633 -     -     -     -
3.7 0.005 0.320 -     -     -
4.7 0.008 0.498 1.000 -     -
```

```
5.7 0.005 0.320 1.000 1.000 -
6.7 0.004 0.249 1.000 1.000 1.000

P value adjustment method: bonferroni
```

```
tukey.test <- agricolae::HSD.test(
  aov1, "logpfuml", group=TRUE, alpha = 0.05); tukey.test
```

```
$statistics
  MSerror Df  Mean   CV   MSD
   0.0183 12 0.303 44.6 0.371

$parameters
   test   name.t ntr StudentizedRange alpha
  Tukey logpfuml   6             4.75  0.05

$means
    tcid2pfu    std r     se    Min   Max   Q25   Q50   Q75
1.7    0.706 0.2082 3 0.0782 0.5857 0.946 0.586 0.586 0.766
2.7    0.455 0.2276 3 0.0782 0.1918 0.586 0.389 0.586 0.586
3.7    0.162 0.0332 3 0.0782 0.1262 0.192 0.147 0.168 0.180
4.7    0.189 0.0750 3 0.0782 0.1262 0.272 0.147 0.168 0.220
5.7    0.162 0.0332 3 0.0782 0.1262 0.192 0.147 0.168 0.180
6.7    0.147 0.0835 3 0.0782 0.0586 0.224 0.109 0.159 0.192

$comparison
NULL

$groups
    tcid2pfu groups
1.7    0.706      a
2.7    0.455     ab
4.7    0.189      b
5.7    0.162      b
3.7    0.162      b
6.7    0.147      b

attr(,"class")
[1] "group"
```

```
# Boxplot of log tcid2pfu:PFU
p <- ggplot(dat1, aes(x = factor(logpfuml), y = tcid2pfu)) +
 geom_boxplot(fill = "grey80", color = "black") +
 labs(x = "log10 PFU per Reaction",
      y = "logarithmic TCID50:PFU ratio") +
 theme_bw()
p
```

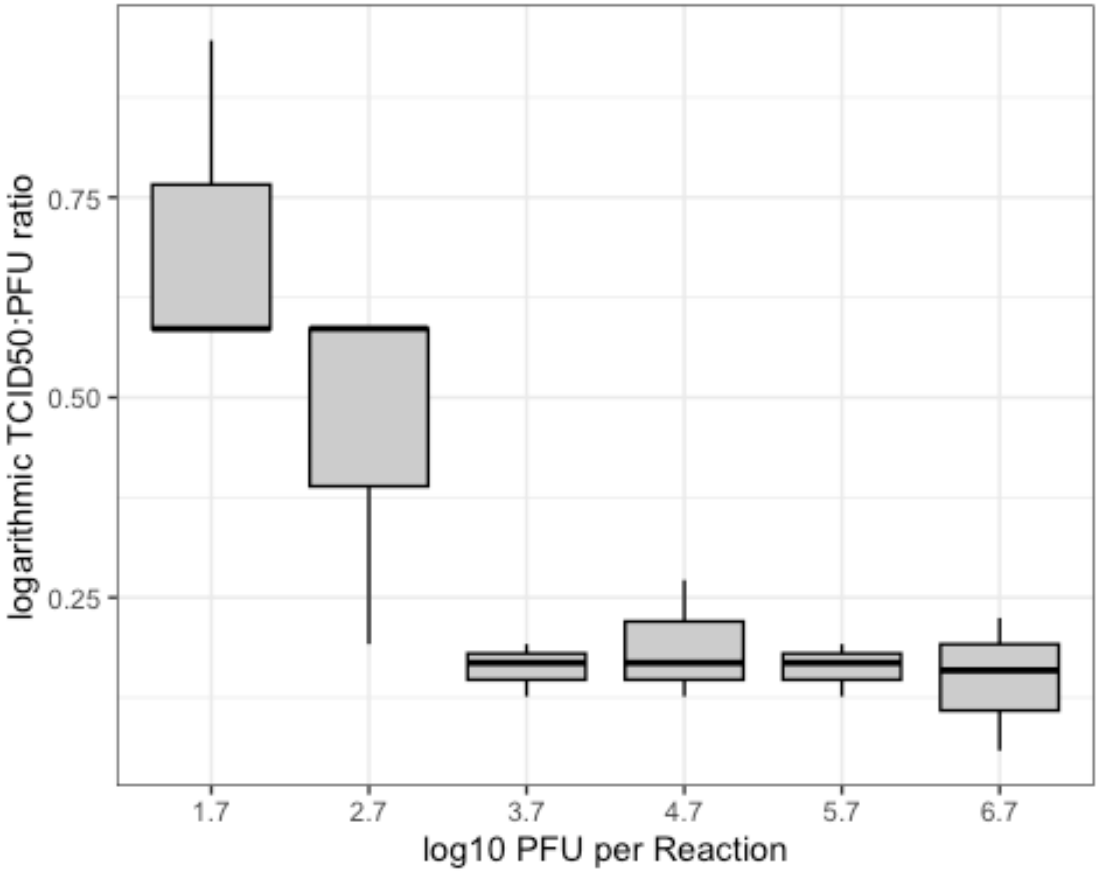


```
# Removing two more low concentrations
dat1 <- dat1[!dat1$dil %in% c(-5,-4), ]

# Average and std error
mean(dat1$logtcid2pfu)
```

```
[1] -0.808
```

```
sd(dat1$logtcid2pfu)/sqrt(length(dat1$logtcid2pfu))
```

```
[1] 0.0484
```

```
mean(dat1$tcid2pfu)
```

```
[1] 0.165
```

```
sd(dat1$tcid2pfu)/sqrt(length(dat1$tcid2pfu))
```

```
[1] 0.0156
```

```
# Whether the log tcid50:pfu is different across dilutions
dat1$logpfuml <- as.factor(dat1$logpfuml)
aov1 <- aov(tcid2pfu ~ logpfuml, data = dat1);summary(aov1)
```

```
             Df  Sum Sq Mean Sq F value Pr(>F)
logpfuml      3 0.00269  0.0009    0.24   0.86
Residuals     8 0.02961  0.0037
```

```
# Pairwise comparison by separating w/ and w/o rnase. pfu_rxn has to be a
factor
pairwise.t.test(dat1$tcid2pfu, dat1$logpfuml,
                p.adjust.method = "bonf",
                alternative = c("two.sided"))
```

```

    Pairwise comparisons using t tests with pooled SD

data:  dat1$tcid2pfu and dat1$logpfuml

    3.7 4.7 5.7
4.7 1   -   -
5.7 1   1   -
6.7 1   1   1

P value adjustment method: bonferroni
```

```
tukey.test <- agricolae::HSD.test(aov1, "logpfuml", group=T, alpha = 0.05);
tukey.test
```

```
$statistics
  MSerror Df  Mean   CV   MSD
   0.0037  8 0.165 36.9 0.159

$parameters
   test   name.t ntr StudentizedRange alpha
  Tukey logpfuml   4             4.53  0.05

$means
    tcid2pfu    std r     se    Min   Max   Q25   Q50   Q75
3.7    0.162 0.0332 3 0.0351 0.1262 0.192 0.147 0.168 0.180
4.7    0.189 0.0750 3 0.0351 0.1262 0.272 0.147 0.168 0.220
5.7    0.162 0.0332 3 0.0351 0.1262 0.192 0.147 0.168 0.180
6.7    0.147 0.0835 3 0.0351 0.0586 0.224 0.109 0.159 0.192

$comparison
NULL

$groups
    tcid2pfu groups
4.7    0.189      a
5.7    0.162      a
3.7    0.162      a
6.7    0.147      a
```

```
attr(,"class")
[1] "group"
```

3.1 Predicting TCID50:PFU over PFU (obs)

Predict log PFU/ml using TCID50:PFU so that we can compare predictions with observed log PFU/ml.

```
# Read data
dat1 <- readRDS("TCID50_plaque assay.RDS")

# Fitting the global model
fit1 <- lm(logpfuml ~ tcid2pfu, data = dat1); summary(fit1)


Call:
lm(formula = logpfuml ~ tcid2pfu, data = dat1)

Residuals:
    Min      1Q  Median      3Q     Max
-2.1062 -0.9678  0.0342  0.8619  2.0708

Coefficients:
            Estimate Std. Error t value Pr(>|t|)
(Intercept)    5.847      0.458   12.77  8.3e-10 ***
tcid2pfu      -5.428      1.190   -4.56  0.00032 ***
---
Signif. codes:  0 '***' 0.001 '**' 0.01 '*' 0.05 '.' 0.1 ' ' 1

Residual standard error: 1.19 on 16 degrees of freedom
Multiple R-squared:  0.565, Adjusted R-squared:  0.538
F-statistic: 20.8 on 1 and 16 DF,  p-value: 0.000321

sqrt(mean(fit1$residuals^2))

[1] 1.13

coef(fit1)

(Intercept)    tcid2pfu
       5.85       -5.43

confint(fit1)

            2.5 % 97.5 %
(Intercept)  4.88   6.82
tcid2pfu    -7.95  -2.91

# Predictions
## Predicted values
pred_fit1 <- predict(fit1,
```

```
                      newdata = dat1,
                      interval = "prediction", level = 0.95) |>
  as.data.frame() |>
  rename(pred_lwr = lwr,
         pred_upr = upr,
         pred_fit = fit)

## conditional means
ci_fit1 <- predict(fit1,
                    newdata = dat1,
                    interval = "confidence", level = 0.95) |>
  as.data.frame() |>
  rename(ci_lwr = lwr,
         ci_upr = upr,
         ci_fit = fit)

## Consolidate Predicted values and conditional means
merged_data <- cbind(data.frame(logpfuml=dat1$logpfuml), pred_fit1, ci_fit1)
merged_data$dif_PFU_Pred_Obs <- merged_data$pred_fit-merged_data$logpfuml

saveRDS(merged_data, "pred_pfu_from_tcid2pfu.RDS")

rm(dat1, pred_fit1,ci_fit1, merged_data)
```

3.2 Agreement analyses and visualizations

To assess the use of TCID50:PFU for predicting log PFU/ml and compare with observed log PFU/ml.

```
dat1 <- readRDS("pred_pfu_from_tcid2pfu.RDS")

# Bland-Altman analysis (Tukey mean-difference)
blandr.statistics (dat1$pred_fit, dat1$logpfuml, sig.level=0.95)
```

```
Bland-Altman Statistics
=======================
t = 5e-15, df = 17, p-value = 1
alternative hypothesis: true bias is not equal to 0

=======================
Number of comparisons:  18
Maximum value for average measures:  6.11
Minimum value for average measures:  1.21
Maximum value for difference in measures:  2.11
Minimum value for difference in measures:  -2.07

Bias:  1.42e-15
Standard deviation of bias:  1.16

Standard error of bias:  0.273
```

```
Standard error for limits of agreement:  0.476

Bias:  1.42e-15
Bias- upper 95% CI:  0.576
Bias- lower 95% CI:  -0.576

Upper limit of agreement:  2.27
Upper LOA- upper 95% CI:  3.27
Upper LOA- lower 95% CI:  1.27

Lower limit of agreement:  -2.27
Lower LOA- upper 95% CI:  -1.27
Lower LOA- lower 95% CI:  -3.27

=======================
Derived measures:
Mean of differences/means:  1.57
Point estimate of bias as proportion of lowest average:  1.18e-13
Point estimate of bias as proportion of highest average 2.32e-14
Spread of data between lower and upper LoAs:  4.54
Bias as proportion of LoA spread:  3.12e-14

=======================
Bias:
 1.42e-15  ( -0.576  to  0.576 )
ULoA:
 2.27  ( 1.27  to  3.27 )
LLoA:
 -2.27  ( -3.27  to  -1.27 )

# Difference between W/O over W/ RNase treatment
mean(dat1$dif_PFU_Pred_Obs)

[1] 1.42e-15

sd(dat1$dif_PFU_Pred_Obs)/sqrt(length(dat1))

[1] 0.41

mean(10^dat1$dif_PFU_Pred_Obs)

[1] 11.8

sd(10^dat1$dif_PFU_Pred_Obs)/sqrt(length(dat1))

[1] 10.6

## check the assumption of normality for the differences
shapiro.test(dat1$dif_PFU_Pred_Obs)
```

```
    Shapiro-Wilk normality test

data:  dat1$dif_PFU_Pred_Obs
W = 1, p-value = 0.9
```

```
## t-test
t.test(
 dat1$pred_fit, dat1$logpfuml,
 alternative = c("two.sided"),
 paired = TRUE)
```

```
    Paired t-test

data:  dat1$pred_fit and dat1$logpfuml
t = 5e-15, df = 17, p-value = 1
alternative hypothesis: true mean difference is not equal to 0
95 percent confidence interval:
 -0.576  0.576
sample estimates:
mean difference
       1.42e-15
```

3.3 Beta distribution

```
iters=1000
## reading data
dat1 <- readRDS("TCID50_plaque assay.RDS")

# scaled value (min-max normalization)
min_logtcid2pfu <- min(dat1$logtcid2pfu)
max_logtcid2pfu <- max(dat1$logtcid2pfu)

dat1$logtcid2pfu_s <- (dat1$logtcid2pfu - min_logtcid2pfu) / (max_logtcid2pfu
- min_logtcid2pfu)

dat1$logtcid2pfu_s[dat1$logtcid2pfu_s == 0] <- 0.00001
dat1$logtcid2pfu_s[dat1$logtcid2pfu_s == 1] <- 0.99999

# Beta dist fit: W/ RNase
fit_beta <- fitdist(dat1$logtcid2pfu_s, "beta",
                    start = list(shape1 = 2, shape2 = 2),
                    method = 'mge'); summary (fit_beta)
```

```
Warning in fitdist(dat1$logtcid2pfu_s, "beta", start = list(shape1 = 2, :
maximum GOF estimation has a default 'gof' argument set to 'CvM'
```

```
Fitting of the distribution ' beta ' by maximum goodness-of-fit
Parameters :
       estimate
```

```
shape1     1.70
shape2     1.88
Loglikelihood:  -12.5   AIC:  29.1   BIC:  30.9

# Bootstrap simulation of uncertainty
f_beta_boot <- bootdist(fit_beta, bootmethod="param", niter = iters)
summary(f_beta_boot)

Parametric bootstrap medians and 95% percentile CI
       Median  2.5% 97.5%
shape1   1.85 0.974  4.47
shape2   2.02 1.045  5.31

# parameters of Beta distribution (scaled)

shape1 <- quantile(f_beta_boot$estim[, 1], probs = c(0.5))
shape2 <- quantile(f_beta_boot$estim[, 2], probs = c(0.5))

rbeta.est.boot <- rbeta (iters, shape1, shape2)

# Plotting density distributions
data_seq <- seq(0, 1, length = 100)
dbeta2 <- dbeta (data_seq, shape1, shape2)

# Create a data frame for the beta distribution to overlay
dat1_beta <- data.frame(x = data_seq, density = dbeta2)

scale_back <- function(x) {
  x * (max_logtcid2pfu - min_logtcid2pfu) + min_logtcid2pfu
}

x_ticks_scaled <- seq(0, 1, length.out = 6)
x_ticks_actual <- scale_back(x_ticks_scaled)

# Summaries
beta_sum <- quantile(rbeta.est.boot, prob=c(0.025, 0.50, 0.975))
(beta_sum_actual <- scale_back (beta_sum))

  2.5%    50%  97.5%
-1.147 -0.646 -0.159

est_0.5 <- beta_sum[2]
est_0.5_actual <- beta_sum_actual[2]

p2 <- ggplot(data=dat1, aes(x = logtcid2pfu_s)) +
 geom_histogram(aes(y = after_stat(density)), bins = 15,
               fill = "darkgray", color = "black") +
 geom_line(data=dat1_beta,
           aes(x = x, y = density),
           color = "#fb6502", linetype = "dashed", linewidth = 0.5) +
```

```
 geom_rug(aes(x = logtcid2pfu_s), sides = "b", color = "black") +
 geom_vline(xintercept = est_0.5,
             color = "#00529b", linetype = "solid", linewidth = 0.5) +
 annotate("text", x = est_0.5,
          y = Inf, label = paste("Median =", round(est_0.5_actual, 2)),
          vjust = 5, hjust = -0.2,
          color = "#00529b", size = 2.5,
          family = "times") +
 scale_x_continuous(breaks = x_ticks_scaled,
                    labels = round(x_ticks_actual, 1)) +
 scale_y_continuous (breaks = c (0, 1, 2, 3, 4, 5),
                    limits = c(0, 5)) +
 labs(
  x = bquote (Log[10]~TCID[50]:PFU~ratio),
  y = "Density") +
 theme_bw (base_family="") +
 theme(
  text = element_text(family = "Times"),
  legend.position = "inside",
  legend.position.inside = c(0.3, 0.9),
  legend.direction="horizontal",
  legend.text=element_text(size = 6, face='bold'),
  legend.title=element_text (size = 6,face='bold'),
  plot.title = element_text(color="black", size=10, face = "bold", hjust =
0.5),
  axis.text.x=element_text(size = 10, color="black", face="bold", hjust =
0.5, vjust = 0.5),
  axis.text.y=element_text (size = 10, color="black", face="bold"),
  axis.title.x = element_text (size = 10, vjust = -1),
  axis.title.y = element_text (size = 10, vjust = 2),
  panel.grid.major = element_line (color="darkgray", linewidth=0.1,
linetype="solid"),
  panel.grid.minor = element_line (color="gray", linewidth=0.1,
linetype="dashed"),
  panel.background = element_rect (fill =  "transparent"),
  legend.background = element_rect (fill = "transparent", color =
"transparent"),
  plot.background = element_rect (fill = "transparent", color =
"transparent")) +
 guides(color = "none")

combined_plot <- ggpubr::ggarrange(p1, #from TCID50 over PFU
                                   p2, ncol = 2,
                                   labels = c("A", "B"),
                                   font.label = list(size=9, face="bold",
family="times"
                                   ))
combined_plot
```

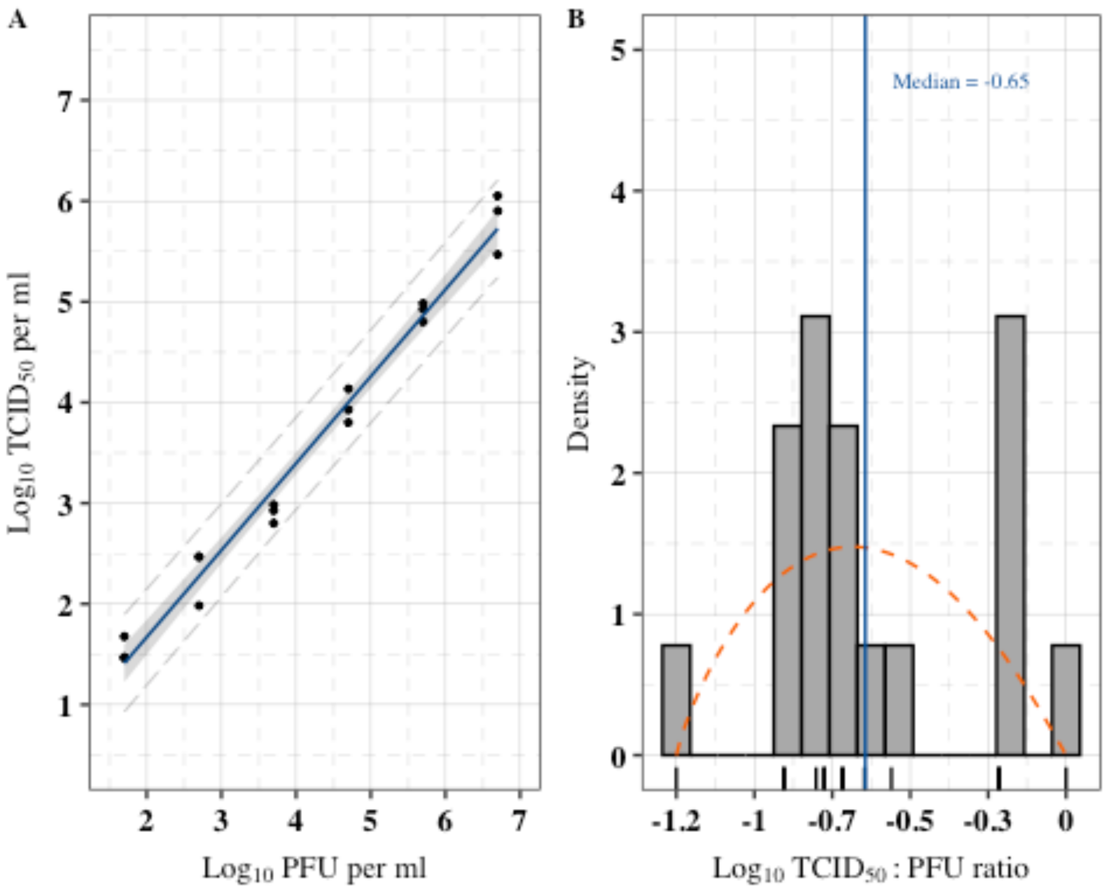


```
ggsave(plot=combined_plot, file="Fig3_TuV_inf_and_beta_dist.tiff", width=18,
height=7, units = c("cm"), dpi=600)

rm(dat1, min_logtcid2pfu, max_logtcid2pfu, iters, fit_beta, shape1, shape2,
f_beta_boot, dbeta2, data_seq, dat1_beta, rbeta.est.boot, est_0.5,
est_0.5_actual, x_ticks_actual, x_ticks_scaled, scale_back, p1, p2)
```

3.4 Model validation through train data (using original data)

```
# Read data
dat1 <- readRDS("TCID50_plaque assay.RDS")

# Initialize a list to store the RMSE results
rmse_results <- list()
r2_results <- list()
train_data_list <- list()
test_data_list <- list()

index <- createDataPartition(dat1$logpfuml,
                             p = 0.70, times = 5, list = TRUE)

# Loop over each partition and check for data size
for (i in 1:length(index)) {
  train_data <- dat1[index[[i]], ]
```

```r
  test_data <- dat1[-index[[i]], ]

  # Check if both train and test sets have more than one row
  if (nrow(train_data) > 1 & nrow(test_data) > 1) {
    # Train the model on train_data
    model <- train(logpfuml ~ tcid2pfu,
                   data = train_data,
                   method = "lm",
                   trControl = trainControl(method = "boot", number = 500))

    # Predict on test data
    pred <- predict(model, newdata = test_data)

    # Calculate RMSE
    rmse <- caret::RMSE(pred = pred, obs = test_data$logpfuml)
    r2 <- caret::R2(pred = pred, obs = test_data$logpfuml)

    # Store the result
    rmse_results[[i]] <- rmse
    r2_results[[i]] <- r2
    train_data_list[[i]] <- train_data
    test_data_list[[i]] <- test_data

  } else {
    # If either train or test set is too small, skip this iteration
    cat("Skipping iteration", i, "due to small data size\n")
  }
}
```

```
Warning in nominalTrainWorkflow(x = x, y = y, wts = weights, info =
trainInfo,
: There were missing values in resampled performance measures.
Warning in nominalTrainWorkflow(x = x, y = y, wts = weights, info =
trainInfo,
: There were missing values in resampled performance measures.
Warning in nominalTrainWorkflow(x = x, y = y, wts = weights, info =
trainInfo,
: There were missing values in resampled performance measures.
Warning in nominalTrainWorkflow(x = x, y = y, wts = weights, info =
trainInfo,
: There were missing values in resampled performance measures.
Warning in nominalTrainWorkflow(x = x, y = y, wts = weights, info =
trainInfo,
: There were missing values in resampled performance measures.
```

```r
# Check if any valid RMSE results were obtained
rmse_results; summary(unlist(rmse_results))
```

```
[[1]]
[1] 0.889
```

```
[[2]]
[1] 0.252

[[3]]
[1] 1.43

[[4]]
[1] 0.872

[[5]]
[1] 1.6

   Min. 1st Qu.  Median    Mean 3rd Qu.    Max. 
  0.252   0.872   0.889   1.007   1.429   1.595 
```

```
r2_results; summary(unlist(r2_results))
```

```
[[1]]
[1] 1

[[2]]
[1] 1

[[3]]
[1] 1

[[4]]
[1] 1

[[5]]
[1] 1

   Min. 1st Qu.  Median    Mean 3rd Qu.    Max. 
      1       1       1       1       1       1 
```

```
train_data_list[1]
```

```
[[1]]
   dil rep logpfuml logTCID50ml logtcid2pfu tcid2pfu
1    0   1      6.7        5.47      -1.232   0.0586
2    0   2      6.7        5.90      -0.799   0.1589
3    0   3      6.7        6.05      -0.649   0.2244
4   -1   1      5.7        4.98      -0.717   0.1918
6   -1   3      5.7        4.93      -0.774   0.1683
7   -2   1      4.7        3.80      -0.899   0.1262
8   -2   2      4.7        4.13      -0.566   0.2719
9   -2   3      4.7        3.93      -0.774   0.1683
10  -3   1      3.7        2.93      -0.774   0.1683
11  -3   2      3.7        2.80      -0.899   0.1262
```

```
12  -3   3      3.7        2.98      -0.717   0.1918
13  -4   1      2.7        2.47      -0.232   0.5861
14  -4   2      2.7        2.47      -0.232   0.5861
15  -4   3      2.7        1.98      -0.717   0.1918
17  -5   2      1.7        1.47      -0.232   0.5857
18  -5   3      1.7        1.68      -0.024   0.9463
```

```
test_data_list

[[1]]
   dil rep logpfuml logTCID50ml logtcid2pfu tcid2pfu
5   -1   2      5.7        4.80      -0.899    0.126
16  -5   1      1.7        1.47      -0.232    0.586

[[2]]
   dil rep logpfuml logTCID50ml logtcid2pfu tcid2pfu
8   -2   2      4.7        4.13      -0.566    0.272
13  -4   1      2.7        2.47      -0.232    0.586

[[3]]
   dil rep logpfuml logTCID50ml logtcid2pfu tcid2pfu
7   -2   1      4.7        3.80      -0.899    0.126
18  -5   3      1.7        1.68      -0.024    0.946

[[4]]
   dil rep logpfuml logTCID50ml logtcid2pfu tcid2pfu
7   -2   1      4.7        3.80      -0.899    0.126
16  -5   1      1.7        1.47      -0.232    0.586

[[5]]
   dil rep logpfuml logTCID50ml logtcid2pfu tcid2pfu
8   -2   2      4.7        4.13      -0.566    0.272
15  -4   3      2.7        1.98      -0.717    0.192
```

```
rm(dat1, r2_results, rmse_results, test_data, train_data)
```

4. RT-qPCR calibration curve

4.1 Data entry and initial cleanup

```
dat1 <- as.data.frame(read_excel("tv_std_curve.xlsx"))

# Remove rows with any NA values
dat1 <- dat1[complete.cases(dat1), ]
dat1$ct <- as.numeric(dat1$ct)
dat1 <- dat1[, c("ct", "log_gc_rxn")]

saveRDS (dat1, "tv_std_curve.RDS")
```

```
rm(dat1)
```

4.2 Regression and outlier assessment

4.2.1 Comparing model fits (w/ or w/o Ct>40)

```
dat1 <- readRDS ("tv_std_curve.RDS")
dat1 <- dat1[complete.cases(dat1), ]

# With Ct higher than 40 (row 59)
# Fit the linear model
fit_w40 <- lm(ct ~ log_gc_rxn,
           data = dat1, na.action=na.omit)
summary(fit_w40)


Call:
lm(formula = ct ~ log_gc_rxn, data = dat1, na.action = na.omit)

Residuals:
    Min      1Q  Median      3Q     Max
-1.1569 -0.3694 -0.0106  0.2567  1.9609

Coefficients:
            Estimate Std. Error t value Pr(>|t|)
(Intercept)  48.6046     0.2474   196.5   <2e-16 ***
log_gc_rxn   -3.8479     0.0415   -92.6   <2e-16 ***
---
Signif. codes:  0 '***' 0.001 '**' 0.01 '*' 0.05 '.' 0.1 ' ' 1

Residual standard error: 0.576 on 55 degrees of freedom
Multiple R-squared:  0.994, Adjusted R-squared:  0.994
F-statistic: 8.58e+03 on 1 and 55 DF,  p-value: <2e-16

# Model diagnosis
opar <- par(mfrow = c(2,2), oma = c(0, 0, 1.1, 0))
plot(fit_w40, las = 1); par(opar)
```

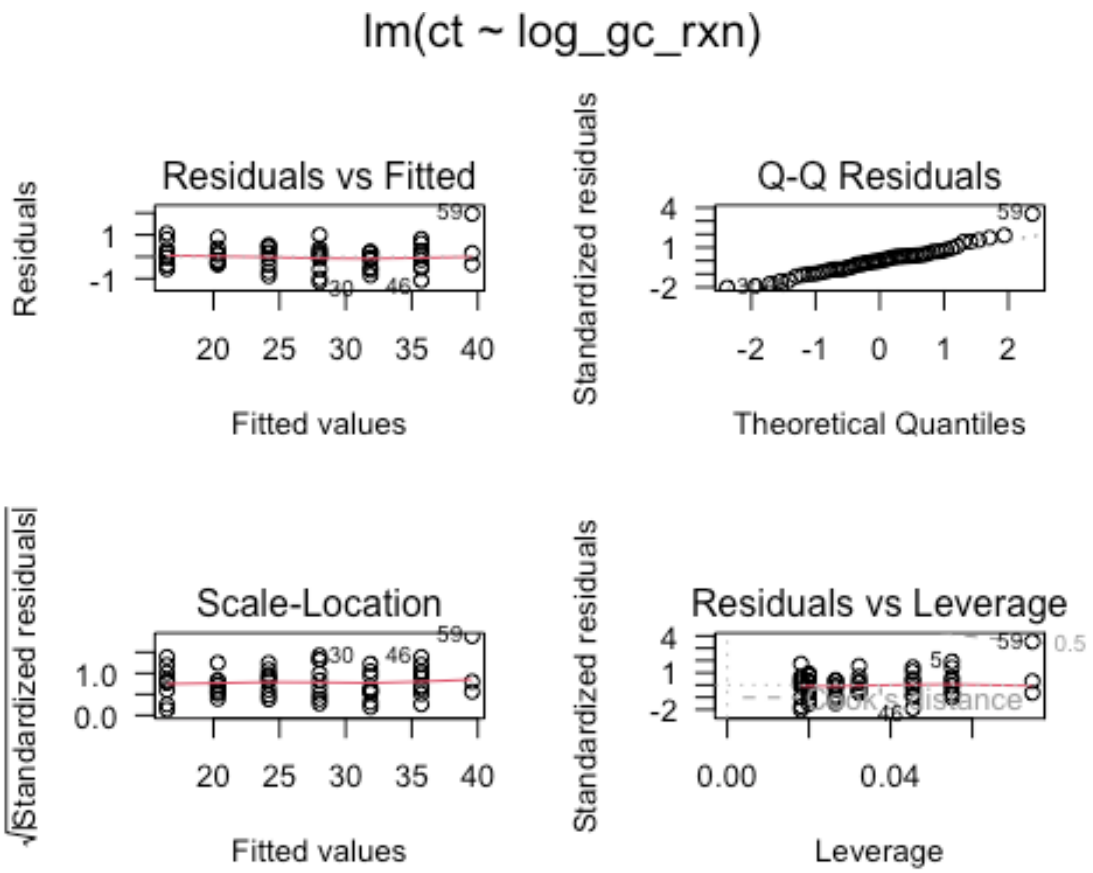


```
# Remove Ct higher than 40 (row 59)
dat2 <- readRDS ("tv_std_curve.RDS")
dat2$ct[dat2$ct>40] <- NA
dat2 <- dat2[complete.cases(dat2), ]

# Model without outlier
fit_wo40 <- lm (ct ~ log_gc_rxn,
            data=dat2, na.action=na.omit)
summary(fit_wo40)


Call:
lm(formula = ct ~ log_gc_rxn, data = dat2, na.action = na.omit)

Residuals:
    Min      1Q  Median      3Q     Max
-1.1082 -0.3672  0.0466  0.3218  1.0392

Coefficients:
            Estimate Std. Error t value Pr(>|t|)
(Intercept)   48.361      0.228     212   <2e-16 ***
log_gc_rxn    -3.811      0.038    -100   <2e-16 ***
---
```

```
Signif. codes:  0 '***' 0.001 '**' 0.01 '*' 0.05 '.' 0.1 ' ' 1

Residual standard error: 0.511 on 54 degrees of freedom
Multiple R-squared:  0.995, Adjusted R-squared:  0.995
F-statistic: 1.01e+04 on 1 and 54 DF,  p-value: <2e-16
```

```
# Model diagnosis of second model
opar <- par(mfrow = c(2,2), oma = c(0, 0, 1.1, 0))
plot(fit_wo40, las = 1); par(opar)
```

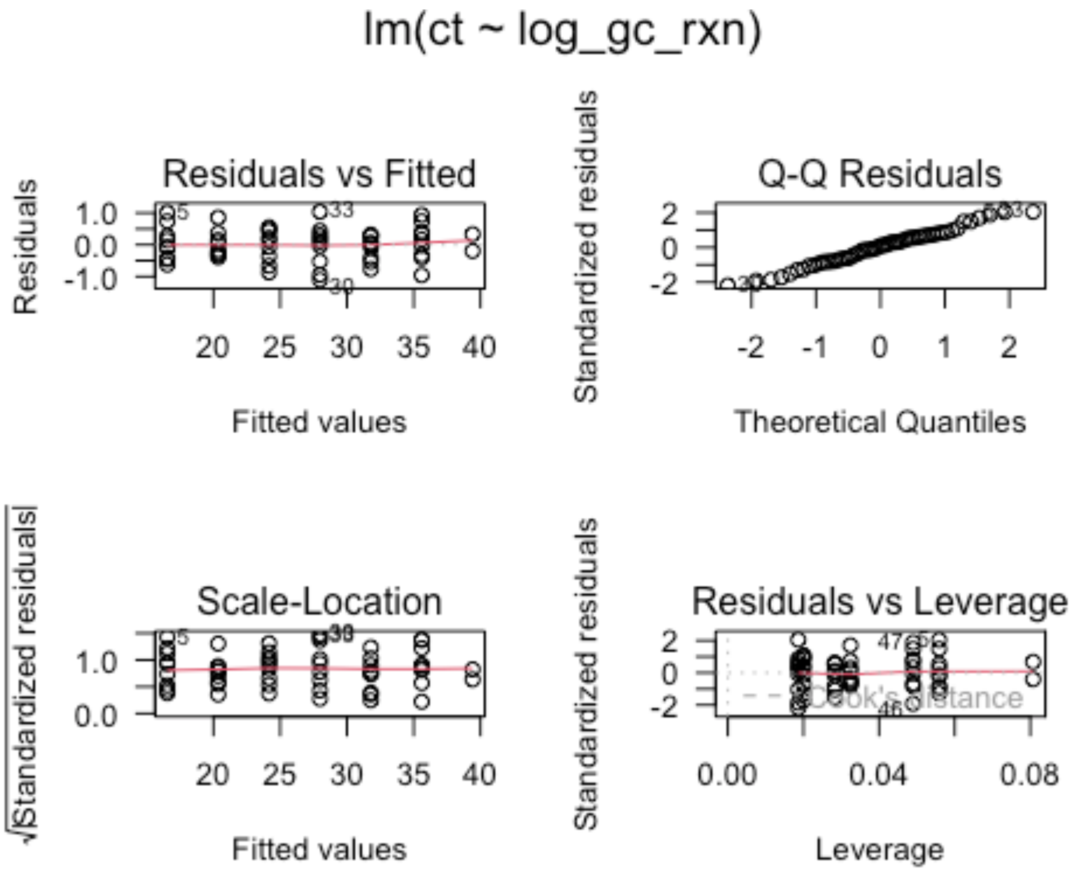


```
## RMSE
sqrt(mean((dat1$ct - predict.lm(fit_w40))^2))
```

```
[1] 0.566
```

```
sqrt(mean((dat2$ct  - predict.lm(fit_wo40))^2))
```

```
[1] 0.502
```

```
## AICc comparison
AICcmodavg::aictab (list (fit_w40=fit_w40, fit_wo40=fit_wo40),
                    second.ord = TRUE)
```

```
Model selection based on AICc:

         K   AICc Delta_AICc AICcWt Cum.Wt     LL
fit_wo40 3  88.2       0.0      1      1 -40.9
fit_w40  3 103.3      15.2      0      1 -48.4
```

```
# RMASE and model estimates of the best model fit
## selected fit (without including ct > 40)
preferred_fit = fit_wo40
sqrt(mean(preferred_fit$residuals^2))
```

```
[1] 0.502
```

```
coef(preferred_fit)
```

```
(Intercept)  log_gc_rxn
      48.36       -3.81
```

```
confint(preferred_fit)
```

```
            2.5 % 97.5 %
(Intercept) 47.90  48.82
log_gc_rxn  -3.89  -3.74
```

```
rm(dat1, dat2, fit_w40, fit_wo40, preferred_fit)
```

4.3 Ct 16-40 predictions of GC w/ and w/o removing Ct>40

4.3.1 Reverse models for predictions

```
#| eval: true
#| echo: true
#| output: true

dat1 <- readRDS ("tv_std_curve.RDS")
dat1 <- dat1[complete.cases(dat1), ]

# With Ct higher than 40 (row 59)
# Fit the linear model
fit_w40 <- lm(log_gc_rxn ~ ct,
           data = dat1, na.action=na.omit)

# Remove Ct higher than 40 (row 59)
dat2 <- readRDS ("tv_std_curve.RDS")
dat2$ct[dat2$ct>40] <- NA
dat2 <- dat2[complete.cases(dat2), ]

# Model without outlier
fit_wo40 <- lm (log_gc_rxn ~ ct,
            data=dat2, na.action=na.omit)
summary(fit_wo40)
```

```
Call:
lm(formula = log_gc_rxn ~ ct, data = dat2, na.action = na.omit)

Residuals:
     Min       1Q   Median       3Q      Max 
-0.29120 -0.09454  0.00495  0.07799  0.27799 

Coefficients:
            Estimate Std. Error t value Pr(>|t|)    
(Intercept)  12.6514     0.0713     177   <2e-16 ***
ct           -0.2610     0.0026    -100   <2e-16 ***
---
Signif. codes:  0 '***' 0.001 '**' 0.01 '*' 0.05 '.' 0.1 ' ' 1

Residual standard error: 0.134 on 54 degrees of freedom
Multiple R-squared:  0.995, Adjusted R-squared:  0.995 
F-statistic: 1.01e+04 on 1 and 54 DF,  p-value: <2e-16
```

```
# Save models to use them for predictions
list (fit_w40=fit_w40, fit_w40s=summary(fit_w40),
      fit_wo40=fit_wo40, fit_wo40s=summary(fit_wo40)) |>
 saveRDS("pcr_model_fits.RDS")
```

4.3.2 Prediction calculations

```
# Calling model fits and data
model_fits <- readRDS("pcr_model_fits.RDS")

dat1 <- readRDS ("tv_std_curve.RDS")
dat1 <- dat1[complete.cases(dat1), ]

# Create a seq of numbers roughly within the limit of cal curve
data_seq <- data.frame(ct=seq(16, 40, length.out = 50))

# Predictions of GC with ct > 40
## conditional means
ci_fit_w <- predict(model_fits$fit_w40,
                    newdata = data_seq,
                    interval = "confidence", level = 0.95) |>
 as.data.frame() |>
 rename(ci_lwr_w = lwr,
        ci_upr_w = upr,
        ci_fit_w = fit)

## Predicted values
pred_fit_w <- predict(model_fits$fit_w40,
                      newdata = data_seq,
                      interval = "prediction", level = 0.95) |>
 as.data.frame() |>
```

```
 rename(pred_lwr_w = lwr,
        pred_upr_w = upr,
        pred_fit_w = fit)

# Predictions of GC without ct > 40
## conditional means
ci_fit_wo <- predict(model_fits$fit_wo40,
                     newdata = data_seq,
                     interval = "confidence", level = 0.95) |>
 as.data.frame() |>
 rename(ci_lwr_wo = lwr,
        ci_upr_wo = upr,
        ci_fit_wo = fit)

## Predicted values
pred_fit_wo <- predict(model_fits$fit_wo40,
                       newdata = data_seq,
                       interval = "prediction", level = 0.95) |>
 as.data.frame() |>
 rename(pred_lwr_wo = lwr,
        pred_upr_wo = upr,
        pred_fit_wo = fit)

## Consolidate Predicted values and conditional means
merged_data <- cbind(data_seq, pred_fit_w, ci_fit_w, pred_fit_wo, ci_fit_wo)

# Difference between conditional mean and predicted values and between
redicted values
merged_data$dif_pred_fit_wo_w <- merged_data$pred_fit_wo -
merged_data$pred_fit_w

saveRDS(merged_data, "pred_gc_Ct16to40.RDS")

rm(merged_data, dat1, pred_fit_w, pred_fit_wo, ci_fit_w, ci_fit_wo)
```

4.3.3 Models agreement analyses and visualizations

```
# Read data
dat1 <- readRDS("pred_gc_Ct16to40.RDS")

# Bland-Altman analysis
blandr.statistics (dat1$pred_fit_wo, dat1$pred_fit_w, sig.level=0.95)

Bland-Altman Statistics
=======================
t = -5, df = 49, p-value = 3e-05
alternative hypothesis: true bias is not equal to 0

=======================
Number of comparisons:  50
```

```
Maximum value for average measures:  8.47
Minimum value for average measures:  2.24
Maximum value for difference in measures:  0.0203
Minimum value for difference in measures:  -0.0456

Bias:  -0.0127
Standard deviation of bias:  0.0196

Standard error of bias:  0.00278
Standard error for limits of agreement:  0.00477

Bias:  -0.0127
Bias- upper 95% CI:  -0.00707
Bias- lower 95% CI:  -0.0182

Upper limit of agreement:  0.0258
Upper LOA- upper 95% CI:  0.0354
Upper LOA- lower 95% CI:  0.0162

Lower limit of agreement:  -0.0511
Lower LOA- upper 95% CI:  -0.0415
Lower LOA- lower 95% CI:  -0.0607

=======================
Derived measures:
Mean of differences/means:  -0.432
Point estimate of bias as proportion of lowest average:  -0.566
Point estimate of bias as proportion of highest average -0.149
Spread of data between lower and upper LoAs:  0.0769
Bias as proportion of LoA spread:  -16.4

=======================
Bias:
 -0.0127  ( -0.0182  to  -0.00707 )
ULoA:
 0.0258  ( 0.0162  to  0.0354 )
LLoA:
 -0.0511  ( -0.0607  to  -0.0415 )
```

```
# Main difference and std dev of difference: Prediction
shapiro.test(dat1$dif_pred_fit_wo_w)
```

```
    Shapiro-Wilk normality test

data:  dat1$dif_pred_fit_wo_w
W = 1, p-value = 0.06
```

```
t.test(
 dat1$pred_fit_wo, dat1$pred_fit_w,
```

```
 alternative = c("two.sided"),
 paired = TRUE)


    Paired t-test

data:  dat1$pred_fit_wo and dat1$pred_fit_w
t = -5, df = 49, p-value = 3e-05
alternative hypothesis: true mean difference is not equal to 0
95 percent confidence interval:
 -0.01823 -0.00707
sample estimates:
mean difference
        -0.0127
```

```
mean(dat1$pred_fit_wo)
```

```
[1] 5.34
```

```
mean(dat1$pred_fit_w)
```

```
[1] 5.36
```

```
mean(dat1$dif_pred_fit_wo_w)
```

```
[1] -0.0127
```

```
sd(dat1$dif_pred_fit_wo_w)/sqrt(length(dat1))
```

```
[1] 0.00524
```

```
rm(dat1)
```

4.4 Ct 40-50 predictions of GC w/ and w/o removing Ct>40

4.4.1 Prediction calculations

```
# Calling model fits
model_fits <- readRDS("pcr_model_fits.RDS")

# calling model fits
dat1 <- readRDS ("tv_std_curve.RDS")
dat1 <- dat1[complete.cases(dat1), ]

# Create a seq of numbers roughly within the limit of cal curve
data_seq <- data.frame(ct=seq(40, 50, length.out = 50))

# Predictions of GC with ct > 40
## conditional means
ci_fit_w <- predict(model_fits$fit_w40,
                    newdata = data_seq,
                    interval = "confidence", level = 0.95) |>
 as.data.frame() |>
```

```
 rename(ci_lwr_w = lwr,
        ci_upr_w = upr,
        ci_fit_w = fit)

## Predicted values
pred_fit_w <- predict(model_fits$fit_w40,
                      newdata = data_seq,
                      interval = "prediction", level = 0.95) |>
 as.data.frame() |>
 rename(pred_lwr_w = lwr,
        pred_upr_w = upr,
        pred_fit_w = fit)

# Predictions of GC without ct > 40
## conditional means
ci_fit_wo <- predict(model_fits$fit_wo40,
                     newdata = data_seq,
                     interval = "confidence", level = 0.95) |>
 as.data.frame() |>
 rename(ci_lwr_wo = lwr,
        ci_upr_wo = upr,
        ci_fit_wo = fit)

## Predicted values
pred_fit_wo <- predict(model_fits$fit_wo40,
                       newdata = data_seq,
                       interval = "prediction", level = 0.95) |>
 as.data.frame() |>
 rename(pred_lwr_wo = lwr,
        pred_upr_wo = upr,
        pred_fit_wo = fit)

# Merge dataframes
merged_data <- cbind(data_seq, pred_fit_w, ci_fit_w, pred_fit_wo, ci_fit_wo)

# Difference between conditional mean and predicted values and between
redicted values
merged_data$dif_pred_fit_wo_w <- merged_data$pred_fit_wo -
merged_data$pred_fit_w

saveRDS(merged_data, "pred_gc_Ct40to50.RDS")

rm(merged_data, dat1, pred_fit_w, pred_fit_wo, ci_fit_w, ci_fit_wo)
```

4.4.2 Models agreement analyses and visualizations

```
# Read data
dat1 <- readRDS("pred_gc_Ct40to50.RDS")

# Bland-Altman analysis (Tukey mean-difference)
```

```
# Conditional means based on different cal curves
blandr.statistics (dat1$pred_fit_wo, dat1$pred_fit_w, sig.level=0.95)

Bland-Altman Statistics
=======================
t = -50, df = 49, p-value = <2e-16
alternative hypothesis: true bias is not equal to 0

=======================
Number of comparisons:  50
Maximum value for average measures:  2.24
Minimum value for average measures:  -0.361
Maximum value for difference in measures:  -0.0456
Minimum value for difference in measures:  -0.0731

Bias:  -0.0594
Standard deviation of bias:  0.00818

Standard error of bias:  0.00116
Standard error for limits of agreement:  0.00199

Bias:  -0.0594
Bias- upper 95% CI:  -0.057
Bias- lower 95% CI:  -0.0617

Upper limit of agreement:  -0.0433
Upper LOA- upper 95% CI:  -0.0393
Upper LOA- lower 95% CI:  -0.0473

Lower limit of agreement:  -0.0754
Lower LOA- upper 95% CI:  -0.0714
Lower LOA- lower 95% CI:  -0.0794

=======================
Derived measures:
Mean of differences/means:  -15.7
Point estimate of bias as proportion of lowest average:  16.5
Point estimate of bias as proportion of highest average -2.66
Spread of data between lower and upper LoAs:  0.0321
Bias as proportion of LoA spread:  -185

=======================
Bias:
 -0.0594  ( -0.0617  to  -0.057 )
ULoA:
 -0.0433  ( -0.0473  to  -0.0393 )
LLoA:
 -0.0754  ( -0.0794  to  -0.0714 )
```

```
# Main difference and std dev of difference: Prediction
shapiro.test(dat1$dif_pred_fit_wo_w)


    Shapiro-Wilk normality test

data:  dat1$dif_pred_fit_wo_w
W = 1, p-value = 0.06
```

```
t.test(
 dat1$pred_fit_wo, dat1$pred_fit_w,
 alternative = c("two.sided"),
 paired = TRUE)


    Paired t-test

data:  dat1$pred_fit_wo and dat1$pred_fit_w
t = -51, df = 49, p-value <2e-16
alternative hypothesis: true mean difference is not equal to 0
95 percent confidence interval:
 -0.0617 -0.0570
sample estimates:
mean difference
        -0.0594
```

```
mean(dat1$pred_fit_wo)

[1] 0.908

mean(dat1$pred_fit_w)

[1] 0.967

mean(dat1$dif_pred_fit_wo_w)

[1] -0.0594

sd(dat1$dif_pred_fit_wo_w)/sqrt(length(dat1))

[1] 0.00219

rm(dat1)
```

4.5 Plot of RT-qPCR calibration curve

4.5.1 Generate predictions and intervals

```
# calling data and model fits after emoving Ct>40
dat1 <- readRDS ("tv_std_curve.RDS")
dat1$ct[dat1$ct>40] <- NA
dat1 <- dat1[complete.cases(dat1), ]
```

```
fit0 <- lm(ct ~ log_gc_rxn, data = dat1)

# Create a sequence of values for logpfuml for prediction
data_seq <- data.frame(log_gc_rxn=seq(min(dat1$log_gc_rxn),
max(dat1$log_gc_rxn), length.out = 20))

pred_fit <- predict(fit0,
                     newdata = data_seq,
                    interval = "prediction", level = 0.95) |>
 as.data.frame() |>
 rename(pred_lwr = lwr,
        pred_upr = upr,
        pred_fit = fit)

ci_fit <- predict(fit0, newdata = data_seq,
                    interval = "confidence", level = 0.95) |>
 as.data.frame() |>
 rename(ci_lwr = lwr,
        ci_upr = upr,
        ci_fit = fit)

merged_data <- cbind(data_seq, pred_fit, ci_fit)

saveRDS(merged_data, "RT-qPCR_cal_curve_predicitons.RDS")

rm(dat1, fit0, pred_fit, ci_fit, merged_data)
```

4.5.2 Plot

```
# calling data and model fits after emoving Ct>40
dat1 <- readRDS ("tv_std_curve.RDS")
dat1$ct[dat1$ct>40] <- NA
dat1 <- dat1[complete.cases(dat1), ]
dat2 <- readRDS("RT-qPCR_cal_curve_predicitons.RDS")

p <- ggplot () +
geom_point (data=dat1,
            aes(x=log_gc_rxn, y=ct),
            color = "black", shape = 16, size=1.2) +
 stat_smooth (data=dat1,
              aes(x=log_gc_rxn, y=ct),
              color = c("#00529b"), linewidth=0.5, method="lm", se = FALSE,
              formula = y~x) +
 geom_ribbon(data = dat2,
             aes(x=log_gc_rxn, ymin = ci_lwr, ymax = ci_upr),
             alpha = 0.2, fill = "gray10") +
 geom_line(data = dat2,
           aes(x=log_gc_rxn, y = pred_lwr),
           linetype = "longdash", color = "gray15", linewidth=0.1) +
 geom_line(data = dat2,
```

```
          aes(x = log_gc_rxn, y = pred_upr),
          linetype = "longdash", color = "gray15", linewidth=0.1) +
 scale_x_continuous (breaks = c (2, 3, 4, 5, 6, 7, 8, 9),
                     limits = c(2, 8.5)) +
 scale_y_continuous (breaks = c (15, 20, 25, 30, 35, 40, 45),
                     limits = c(15, 45)) +
 labs (x = bquote (Log[10]~Genomic~Copies~Per~Reaction), size = 3,
       y= bquote (Cycle~Threshold~(Ct)), size = 3) +
 theme_bw (base_family="") +
 theme(
  text = element_text(family = "Times"),
  legend.position = "bottom",
  legend.direction="horizontal",
  legend.text=element_text(size = 6, face = "bold"),
  legend.title=element_text (size = 6, face = "bold"),
  plot.title = element_text(color="black", size=10, face = "bold", hjust =
0.5),
  axis.text.x=element_text(size = 10, color="black", face="bold", hjust =
0.5, vjust = 0.5),
  axis.text.y=element_text (size = 10, color="black", face="bold"),
  axis.title.x = element_text (size = 10, vjust = -1),
  axis.title.y = element_text (size = 10, vjust = 2),
  panel.grid.major = element_line (color="darkgray", linewidth=0.1,
linetype="solid"),
  panel.grid.minor = element_line (color="gray", linewidth=0.1,
linetype="dashed"),
  panel.background = element_rect (fill =  "transparent"),
  legend.background = element_rect (fill = "transparent", color =
"transparent"),
  plot.background = element_rect (fill = "transparent", color =
"transparent")) +
 guides(color = "none")

 p
```

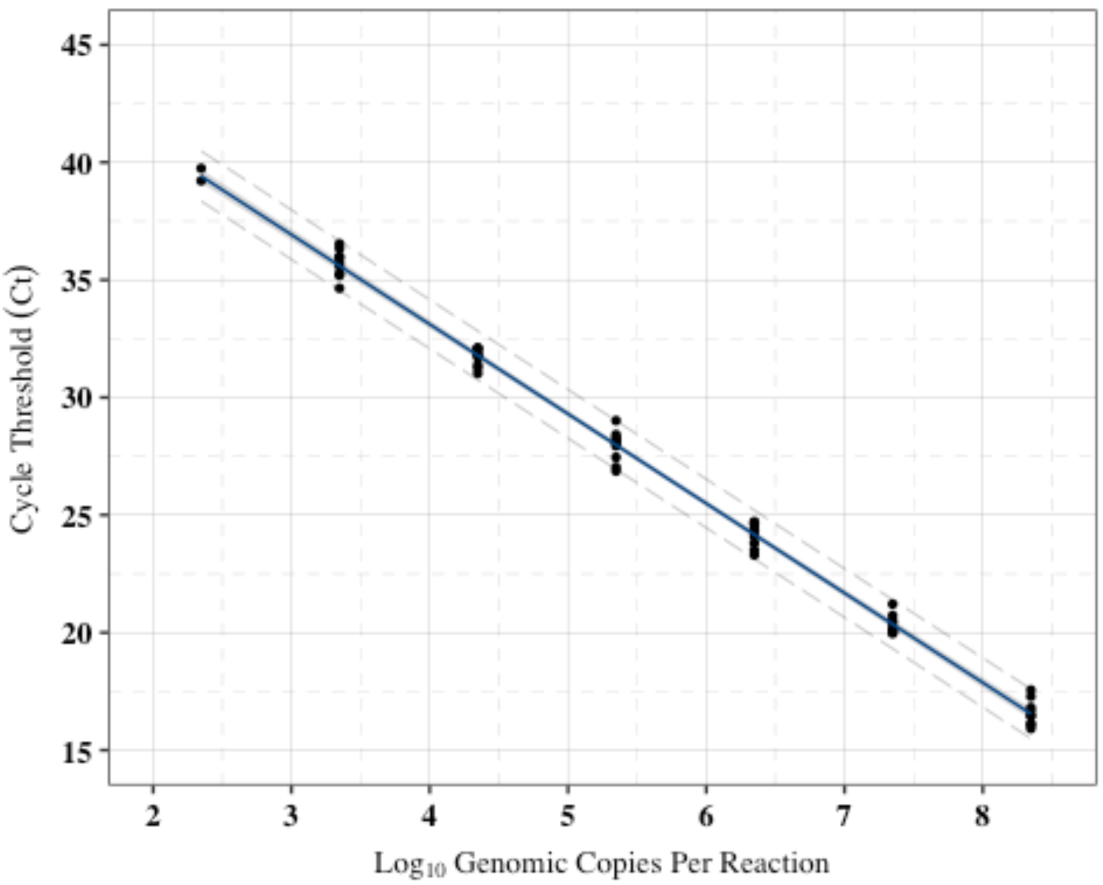


```
ggsave(plot=p, file="Fig4_tv_cal_curve_wo_outlier.tiff", width=8, height=5,
units = c("cm"))
```

5. GC over PFU

5.1 Data entry and initial cleanup

```
dat1 <- as.data.frame(read_excel("pfu_ct_022124.xlsx"))
dat1$dil <- as.factor(dat1$dil)
dat1$rnase <- as.factor(dat1$rnase)
dat1$pfu_rxn <- as.numeric(dat1$pfu_rxn)
dat1$ct <- as.numeric(dat1$ct)

Warning: NAs introduced by coercion

# Ct values less than 10 or higher than 40 (it  belogs to -1 log PFU/ml==>
let's keep)
dat1 |>
 filter(ct>40)

  dil rnase   ct pfu_rxn rep
1   6     w 41.3    -1.2   1
```

```
dat1$ct[dat1$ct>40] <- NA
# "1   6     w 41.26563    -1.2   1" was removed.

dat1 |>
 filter(ct<=10)

[1] dil     rnase   ct      pfu_rxn rep
<0 rows> (or 0-length row.names)

dat1$ct[dat1$ct<=10] <- NA # to replace with NA. No datapoint was removed.

saveRDS(dat1, "pfu_ct_022124_cleanedup.RDS")
rm(dat1)
```

5.2 Calculate GC and averaging tech reps

```
# Calling model fits and data
model_fits <- readRDS("pcr_model_fits.RDS")
dat1 <- readRDS ("pfu_ct_022124_cleanedup.RDS")

## Predicted values
pred_fit <- predict(model_fits$fit_wo40,
                    newdata = dat1,
                    interval = "prediction", level = 0.95) |>
 as.data.frame() |>
 rename(pred_lwr = lwr,
        pred_upr = upr,
        pred_fit = fit)

ci_fit <- predict(model_fits$fit_wo40,
                  newdata = dat1,
                  interval = "confidence", level = 0.95) |>
 as.data.frame() |>
 rename(ci_lwr = lwr,
        ci_upr = upr,
        ci_fit = fit)

merged_data <- cbind(dat1, pred_fit, ci_fit)

str(merged_data)

'data.frame':   84 obs. of  11 variables:
 $ dil     : Factor w/ 7 levels "0","1","2","3",..: 1 1 1 1 2 2 2 2 3 3 ...
 $ rnase   : Factor w/ 2 levels "w","wo": 1 1 2 2 1 1 2 2 1 1 ...
 $ ct      : num  NA 16.4 16.5 16.2 21.2 ...
 $ pfu_rxn : num  4.8 4.8 4.8 4.8 3.8 3.8 3.8 3.8 2.8 2.8 ...
 $ rep     : num  1 1 1 1 1 1 1 1 1 1 ...
 $ pred_fit: num  NA 8.37 8.36 8.41 7.12 ...
 $ pred_lwr: num  NA 8.09 8.08 8.14 6.85 ...
 $ pred_upr: num  NA 8.64 8.63 8.69 7.39 ...
 $ ci_fit  : num  NA 8.37 8.36 8.41 7.12 ...
```

```
 $ ci_lwr  : num  NA 8.3 8.29 8.35 7.08 ...
 $ ci_upr  : num  NA 8.43 8.42 8.48 7.17 ...
```

```
dat1 <- merged_data[complete.cases(merged_data), ] |>
 relocate(ct, .after=rep)

# saving data as Excel file
write_xlsx(dat1, "pred_GCofPFU_from_ct.xlsx")

# saving data as RDS file
saveRDS(dat1, "pred_GCofPFU_from_ct.RDS")

rm(dat1)

# Averaging tech reps
dat1 <- readRDS("pred_GCofPFU_from_ct.RDS") |>
 group_by(dil, pfu_rxn, rnase, rep) |>
 summarise(across(pred_fit:ci_upr, \(x) mean (x, na.rm= TRUE))) |>
 as.data.frame()
```

```
`summarise()` has grouped output by 'dil', 'pfu_rxn', 'rnase'. You can
override
using the `.groups` argument.
```

```
# Calculate GC:PFU ratio
dat1$loggc2pfu <- dat1$pred_fit-dat1$pfu_rxn
dat1$gc2pfu <- (10^dat1$pred_fit)/(10^dat1$pfu_rxn)

saveRDS (dat1, "pred_GCfromPFU_ave.RDS")
write_xlsx (dat1, "pred_GCfromPFU_ave.xlsx")

rm(dat1, merged_data, pred_fit, ci_fit)
```

5.2.1 GC over PFU Regression W/ and W/O RNase

```
# Read data
dat1_w <- readRDS("pred_GCfromPFU_ave.RDS") |>
 dplyr::filter(rnase=="w") |>
 as.data.frame()

dat1_wo <- readRDS("pred_GCfromPFU_ave.RDS") |>
 dplyr::filter(rnase=="wo") |>
 as.data.frame()

## model fit w/ RNase
fit_w <- lm (pred_fit ~ pfu_rxn,
            data=dat1_w, na.action=na.omit)
summary(fit_w)
```

```
Call:
```

```
lm(formula = pred_fit ~ pfu_rxn, data = dat1_w, na.action = na.omit)

Residuals:
    Min      1Q  Median      3Q     Max 
-0.3481 -0.0512  0.0591  0.1395  0.2460 

Coefficients:
            Estimate Std. Error t value Pr(>|t|)    
(Intercept)   3.8407     0.0535    71.7   <2e-16 ***
pfu_rxn       0.9307     0.0199    46.8   <2e-16 ***
---
Signif. codes:  0 '***' 0.001 '**' 0.01 '*' 0.05 '.' 0.1 ' ' 1

Residual standard error: 0.182 on 19 degrees of freedom
Multiple R-squared:  0.991, Adjusted R-squared:  0.991 
F-statistic: 2.19e+03 on 1 and 19 DF,  p-value: <2e-16
```

```r
## Model diagnosis w/ RNase
opar <- par(mfrow = c(2,2), oma = c(0, 0, 1.1, 0))
plot(fit_w, las = 1); par(opar)
```

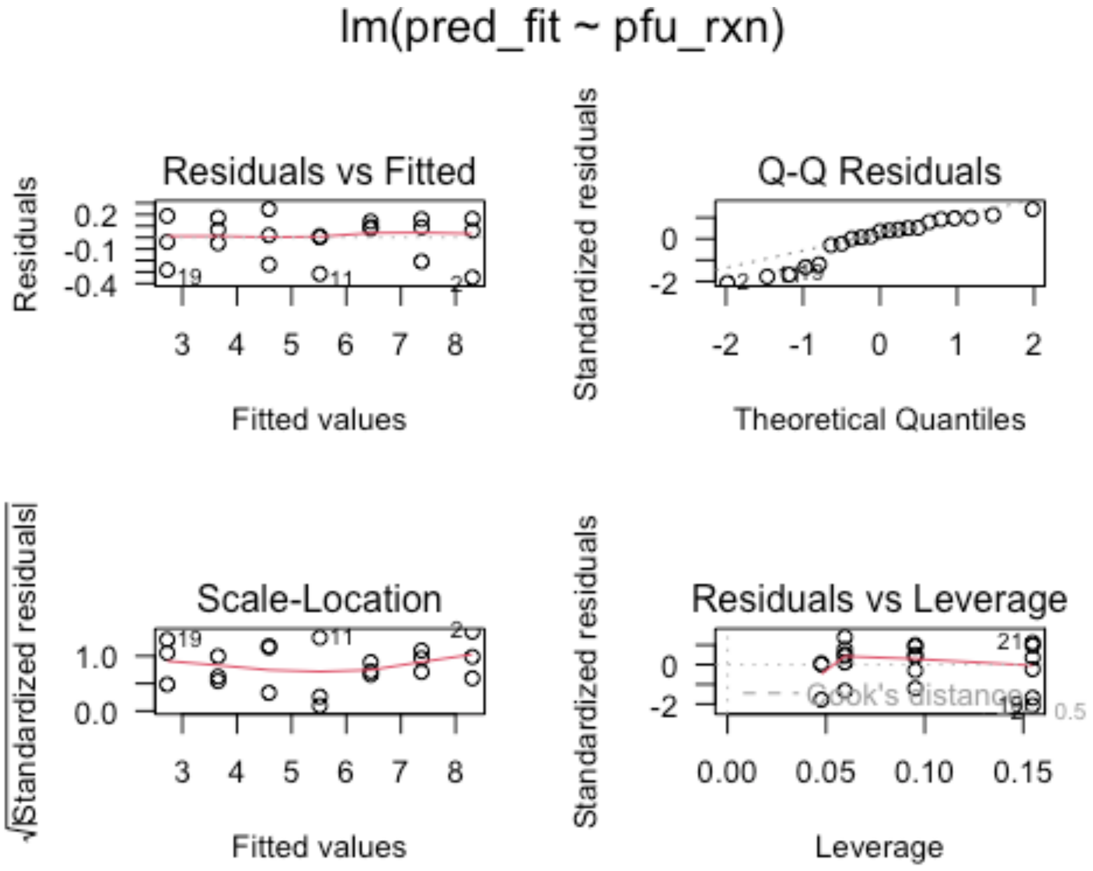


```r
## model fit w/o RNase
fit_wo <- lm (pred_fit ~ pfu_rxn,
```

```
              data=dat1_wo, na.action=na.omit)
summary(fit_wo)


Call:
lm(formula = pred_fit ~ pfu_rxn, data = dat1_wo, na.action = na.omit)

Residuals:
    Min      1Q  Median      3Q     Max 
-0.4018 -0.1054 -0.0347  0.1941  0.3978 

Coefficients:
            Estimate Std. Error t value Pr(>|t|)    
(Intercept)   4.0683     0.0638    63.8   <2e-16 ***
pfu_rxn       0.9284     0.0237    39.2   <2e-16 ***
---
Signif. codes:  0 '***' 0.001 '**' 0.01 '*' 0.05 '.' 0.1 ' ' 1

Residual standard error: 0.217 on 19 degrees of freedom
Multiple R-squared:  0.988, Adjusted R-squared:  0.987 
F-statistic: 1.53e+03 on 1 and 19 DF,  p-value: <2e-16

## Model diagnosis w/o RNase
opar <- par(mfrow = c(2,2), oma = c(0, 0, 1.1, 0))
plot(fit_wo, las = 1); par(opar)
```

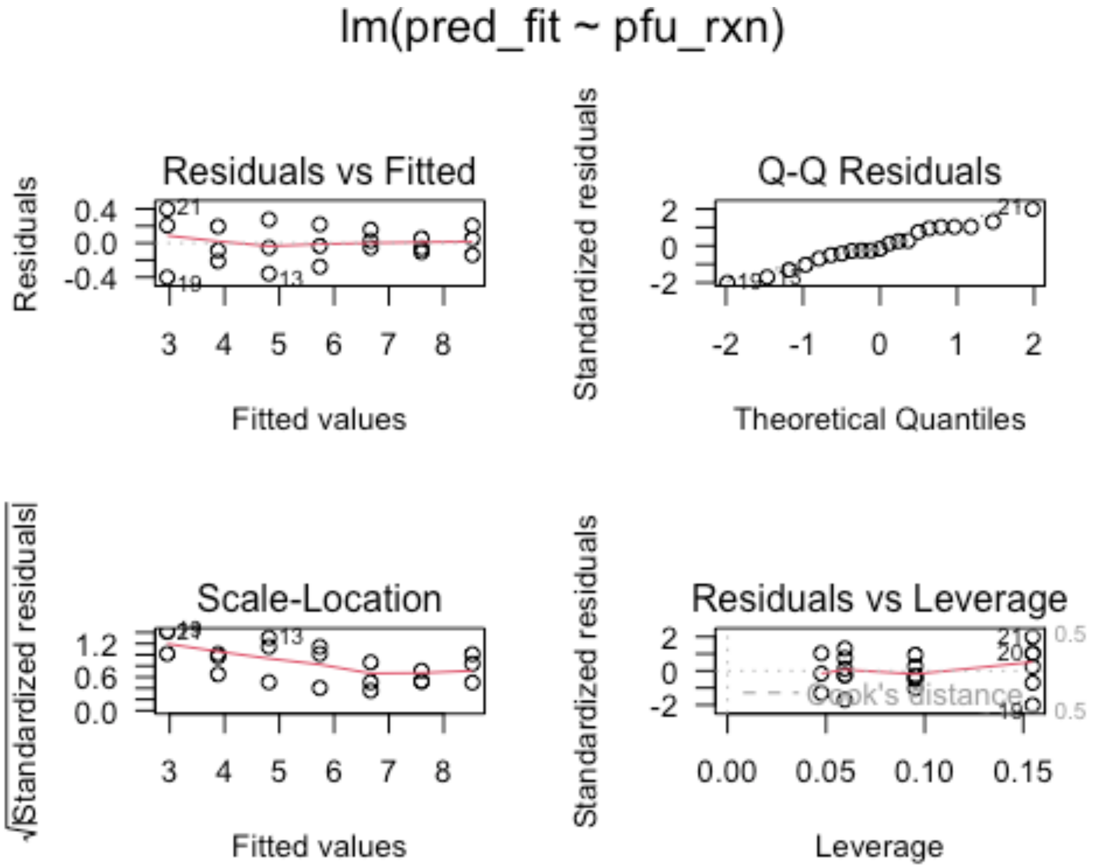


```
# Summary of coefficients and 95% CI
coef(fit_w)

(Intercept)     pfu_rxn
      3.841       0.931

confint(fit_w)

            2.5 % 97.5 %
(Intercept) 3.729  3.953
pfu_rxn     0.889  0.972

coef(fit_wo)

(Intercept)     pfu_rxn
      4.068       0.928

confint(fit_wo)

            2.5 % 97.5 %
(Intercept) 3.935  4.202
pfu_rxn     0.879  0.978

## Saving model fits
list(fit_w=fit_w, fit_ws=summary(fit_w),
```

```
    fit_wo=fit_wo, fit_wos=summary(fit_wo)) |>
 saveRDS("gc_pfu_model_fits.RDS")
```

5.3 Correlation b/w GC and PFU

```
dat1_w <- readRDS("pred_GCfromPFU_ave.RDS") |>
 dplyr::filter(rnase=="w") |>
 as.data.frame()

dat1_wo <- readRDS("pred_GCfromPFU_ave.RDS") |>
 dplyr::filter(rnase=="wo") |>
 as.data.frame()

# Correlations bw pfu_rxn and gc_rxn ####
cor_w <- cor.test (dat1_w$pfu_rxn, dat1_w$pred_fit,
                   method = c("pearson")); cor_w


    Pearson's product-moment correlation

data:  dat1_w$pfu_rxn and dat1_w$pred_fit
t = 47, df = 19, p-value <2e-16
alternative hypothesis: true correlation is not equal to 0
95 percent confidence interval:
 0.989 0.998
sample estimates:
  cor
0.996

cor_wo <- cor.test (dat1_wo$pfu_rxn, dat1_wo$pred_fit,
                   method = c("pearson")); cor_wo


    Pearson's product-moment correlation

data:  dat1_wo$pfu_rxn and dat1_wo$pred_fit
t = 39, df = 19, p-value <2e-16
alternative hypothesis: true correlation is not equal to 0
95 percent confidence interval:
 0.985 0.998
sample estimates:
  cor
0.994

# Fisher's z to compare the two correlations
r1.jk <- cor_w$estimate
n1 <- nrow(dat1_w)
r2.hm <- cor_wo$estimate
n2 <- nrow(dat1_wo)

cocor::cocor.indep.groups(
```

```
 r1.jk, r2.hm, n1, n2,
 alpha = 0.05,
 alternative = "two.sided",
 test = "all"
)
```

```
  Results of a comparison of two correlations based on independent groups

Comparison between r1.jk = 0.996 and r2.hm = 0.994
Difference: r1.jk - r2.hm = 0.0018
Group sizes: n1 = 21, n2 = 21
Null hypothesis: r1.jk is equal to r2.hm
Alternative hypothesis: r1.jk is not equal to r2.hm (two-sided)
Alpha: 0.05

fisher1925: Fisher's z (1925)
  z = 0.5291, p-value = 0.5968
  Null hypothesis retained

zou2007: Zou's (2007) confidence interval
  95% confidence interval for r1.jk - r2.hm: -0.0057 0.0114
  Null hypothesis retained (Interval includes 0)
```

```
rm(dat1_w, dat1_wo, cor_w, cor_wo, r1.jk, n1, r2.hm, n2)
```

5.4 GC over PFU Predictions

```
# Predictions of GC
## data, model fits, and generate seq
dat1 <- readRDS("pred_GCfromPFU_ave.RDS")
model_fits <- readRDS("gc_pfu_model_fits.RDS")

data_seq <- data.frame(pfu_rxn=seq(min(dat1$pfu_rxn), max(dat1$pfu_rxn),
length.out = 50))

## W/ RNase
pred_fit_w <- predict(model_fits$fit_w,
                      newdata = data_seq,
                      interval = "prediction", level = 0.95) |>
 as.data.frame() |>
 rename(pred_lwr_w = lwr,
        pred_upr_w = upr,
        pred_fit_w = fit)

ci_fit_w <- predict(model_fits$fit_w,
                      newdata = data_seq,
                      interval = "confidence", level = 0.95)  |>
 as.data.frame() |>
 rename(ci_lwr_w = lwr,
        ci_upr_w = upr,
```

```
        ci_fit_w = fit)

## W/o RNase
pred_fit_wo <- predict(model_fits$fit_wo,
                       newdata = data_seq,
                       interval = "prediction", level = 0.95) |>
 as.data.frame() |>
 rename(pred_lwr_wo = lwr,
        pred_upr_wo = upr,
        pred_fit_wo = fit)

ci_fit_wo <- predict(model_fits$fit_wo,
                       newdata = data_seq,
                       interval = "confidence", level = 0.95)  |>
 as.data.frame() |>
 rename(ci_lwr_wo = lwr,
        ci_upr_wo = upr,
        ci_fit_wo = fit)

merged_data <- cbind(data_seq, pred_fit_w, ci_fit_w, pred_fit_wo, ci_fit_wo)

merged_data$pred_dif_wo_w <- merged_data$pred_fit_wo - merged_data$pred_fit_w

saveRDS(merged_data, "GCvsPFU_prediciton.RDS")

rm(dat1, data_seq, pred_fit_w, pred_fit_wo, merged_data)
```

5.4.1 Models agreement analyses and visualizations

```
# Bland-Altman analysis (Tukey mean-difference)
dat1 <- readRDS("GCvsPFU_prediciton.RDS")

blandr.statistics (dat1$pred_fit_wo, dat1$pred_fit_w, sig.level=0.95)

Bland-Altman Statistics
=======================
t = 400, df = 49, p-value = <2e-16
alternative hypothesis: true bias is not equal to 0

=======================
Number of comparisons:  50
Maximum value for average measures:  8.42
Minimum value for average measures:  2.84
Maximum value for difference in measures:  0.23
Minimum value for difference in measures:  0.216

Bias:  0.223
Standard deviation of bias:  0.00419

Standard error of bias:  0.000592
```

```
Standard error for limits of agreement:  0.00102

Bias:  0.223
Bias- upper 95% CI:  0.225
Bias- lower 95% CI:  0.222

Upper limit of agreement:  0.232
Upper LOA- upper 95% CI:  0.234
Upper LOA- lower 95% CI:  0.23

Lower limit of agreement:  0.215
Lower LOA- upper 95% CI:  0.217
Lower LOA- lower 95% CI:  0.213

=======================
Derived measures:
Mean of differences/means:  4.4
Point estimate of bias as proportion of lowest average:  7.87
Point estimate of bias as proportion of highest average 2.65
Spread of data between lower and upper LoAs:  0.0164
Bias as proportion of LoA spread:  1362

=======================
Bias:
 0.223  ( 0.222  to  0.225 )
ULoA:
 0.232  ( 0.23  to  0.234 )
LLoA:
 0.215  ( 0.213  to  0.217 )

# Main difference and std dev of difference: Confidence
## check the assumption of normality for the differences
shapiro.test(dat1$pred_dif_wo_w)


    Shapiro-Wilk normality test

data:  dat1$pred_dif_wo_w
W = 1, p-value = 0.06

## t-test
t.test(
 dat1$pred_fit_wo, dat1$pred_fit_w,
 alternative = c("two.sided"),
 paired = TRUE)


    Paired t-test

data:  dat1$pred_fit_wo and dat1$pred_fit_w
```

```
t = 377, df = 49, p-value <2e-16
alternative hypothesis: true mean difference is not equal to 0
95 percent confidence interval:
 0.222 0.225
sample estimates:
mean difference
          0.223
```

```
# Difference between W/O over W/ RNase treatment
dat1 <- readRDS("GCvsPFU_prediciton.RDS")
mean(dat1$pred_dif_wo_w)
```

```
[1] 0.223
```

```
sd(dat1$pred_dif_wo_w)/sqrt(length(dat1))
```

```
[1] 0.00112
```

```
mean(10^dat1$pred_dif_wo_w)
```

```
[1] 1.67
```

```
sd(10^dat1$pred_dif_wo_w)/sqrt(length(dat1))
```

```
[1] 0.00431
```

```
# Whether the w/o - w/ differ among different concentrations
aov1 <- lm(pred_dif_wo_w ~ pfu_rxn, data = dat1); summary(aov1)
```

```

Call:
lm(formula = pred_dif_wo_w ~ pfu_rxn, data = dat1)

Residuals:
      Min        1Q    Median        3Q       Max 
-8.97e-16 -2.67e-16 -4.52e-17  2.06e-16  9.16e-16 

Coefficients:
             Estimate Std. Error   t value Pr(>|t|)    
(Intercept)  2.28e-01   8.10e-17  2.81e+15   <2e-16 ***
pfu_rxn     -2.34e-03   3.21e-17 -7.30e+13   <2e-16 ***
---
Signif. codes:  0 '***' 0.001 '**' 0.01 '*' 0.05 '.' 0.1 ' ' 1

Residual standard error: 4.01e-16 on 48 degrees of freedom
Multiple R-squared:     1,  Adjusted R-squared:     1 
F-statistic: 5.34e+27 on 1 and 48 DF,  p-value: <2e-16
```

```
rm(dat1, aov1)
```

5.5 Plot of GC over PFU

```
dat1 <- readRDS("pred_GCfromPFU_ave.RDS")
dat1 <- dat1[complete.cases(dat1), ]

dat2 <- readRDS("GCvsPFU_prediciton.RDS")

p_w <- ggplot () +
geom_point (data=dat1 |> filter(rnase == "w"),
            aes(x=pfu_rxn, y=pred_fit),
            color = "black", shape = 16, size=1.2) +
 stat_smooth (data=dat1 |> filter(rnase == "w"),
              aes(x=pfu_rxn, y=pred_fit),
              color = c("#00529b"), linewidth=0.5, method="lm", se = FALSE,
              formula = y~x) +
 geom_ribbon(data = dat2,
             aes(x=pfu_rxn, ymin = ci_lwr_w, ymax = ci_upr_w),
             alpha = 0.2, fill = "gray30") +
 geom_line(data = dat2,
           aes(x = pfu_rxn, y = pred_lwr_w),
           linetype = "longdash", color = "gray15", linewidth=0.1) +
 geom_line(data = dat2,
           aes(x = pfu_rxn, y = pred_upr_w),
           linetype = "longdash", color = "gray15", linewidth=0.1) +
  scale_x_continuous (breaks = c (-1, 0, 1, 2, 3, 4, 5),
                      limits = c(-1.2, 5)) +
 scale_y_continuous (breaks = c (2, 3, 4, 5, 6, 7, 8, 9),
                      limits = c(2, 9.1)) +
 labs (title = "With RNase",
       x = bquote (Log[10]~PFU~per~reaction), size = 3,
       y= bquote (Log[10]~GC~per~reaction), size = 3) +
 theme_bw (base_family="") +
 theme(
  text = element_text(family = "Times"),
  legend.position = "inside",
  legend.position.inside = c(0.3, 0.9),
  legend.direction="horizontal",
  legend.text=element_text(size = 6, face='bold'),
  legend.title=element_text (size = 6,face='bold'),
  plot.title = element_text(color="black", size=10, face="bold", hjust = 0.5,
vjust=0.5),
  axis.text.x=element_text(size = 10, color="black", face="bold", hjust =
0.5, vjust = 0.5),
  axis.text.y=element_text (size = 10, color="black", face="bold"),
  axis.title.x = element_text (size = 10, vjust = -1),
  axis.title.y = element_text (size = 10, vjust = 2),
  panel.grid.major = element_line (color="darkgray", linewidth=0.1,
linetype="solid"),
  panel.grid.minor = element_line (color="gray", linewidth=0.1,
linetype="dashed"),
```

```
  panel.background = element_rect (fill =  "transparent"),
  legend.box.background=element_rect(fill="transparent",
color="transparent"),
  legend.background = element_rect (fill = "transparent", color =
"transparent"),
  legend.key=element_rect(fill="transparent", color="transparent"),
  plot.background = element_rect (fill = "transparent", color =
"transparent")) +
 guides(color=guide_legend(override.aes=list(fill=NA)))

p_wo <- ggplot () +
geom_point (data=dat1 |> filter(rnase == "wo"),
            aes(x=pfu_rxn, y=pred_fit),
            color = "black", shape = 16, size=1.2) +
 stat_smooth (data=dat1 |> filter(rnase == "wo"),
              aes(x=pfu_rxn, y=pred_fit),
              color = c("#00529b"), linewidth=0.5, method="lm", se = FALSE,
              formula = y~x) +
 geom_ribbon(data = dat2,
             aes(x= pfu_rxn, ymin = ci_lwr_wo, ymax = ci_upr_wo),
             alpha = 0.2, fill = "gray30") +
 geom_line(data = dat2,
           aes(x = pfu_rxn, y = pred_lwr_wo),
           linetype = "longdash", color = "gray15", linewidth=0.1) +
 geom_line(data = dat2,
           aes(x = pfu_rxn, y = pred_upr_wo),
           linetype = "longdash", color = "gray15", linewidth=0.1) +
 scale_x_continuous (breaks = c (-1, 0, 1, 2, 3, 4, 5),
                     limits = c(-1.2, 5)) +
 scale_y_continuous (breaks = c (2, 3, 4, 5, 6, 7, 8, 9),
                     limits = c(2, 9.1)) +
 labs (title = "Without RNase",
       x = bquote (Log[10]~PFU~per~reaction), size = 3,
       y ="") +
 theme_bw (base_family="") +
 theme(
  text = element_text(family = "Times"),
  legend.position = "inside",
  legend.position.inside = c(0.3, 0.9),
  legend.direction="horizontal",
  legend.text=element_text(size = 6, face='bold'),
  legend.title=element_text (size = 6,face='bold'),
  plot.title = element_text(color="black", size=10, face="bold", hjust = 0.5,
vjust=0.5),
  axis.text.x=element_text(size = 10, color="black", face="bold", hjust =
0.5, vjust = 0.5),
  axis.text.y=element_text (size = 10, color="black", face="bold"),
  axis.title.x = element_text (size = 10, vjust = -1),
  axis.title.y = element_text (size = 10, vjust = 2),
  panel.grid.major = element_line (color="darkgray", linewidth=0.1,
```

```
linetype="solid"),
  panel.grid.minor = element_line (color="gray", linewidth=0.1,
linetype="dashed"),
  panel.background = element_rect (fill =  "transparent"),
  legend.box.background=element_rect(fill="transparent",
color="transparent"),
  legend.background = element_rect (fill = "transparent", color =
"transparent"),
  legend.key=element_rect(fill="transparent", color="transparent"),
  plot.background = element_rect (fill = "transparent", color =
"transparent")) +
 guides(color=guide_legend(override.aes=list(fill=NA)))

combined_plot <- ggpubr::ggarrange(p_w, p_wo,
                                   ncol = 2,
                                   labels = c("A", "B"),
                                   font.label = list(size=9, face="bold",
family="times"
                                   ))
combined_plot
```

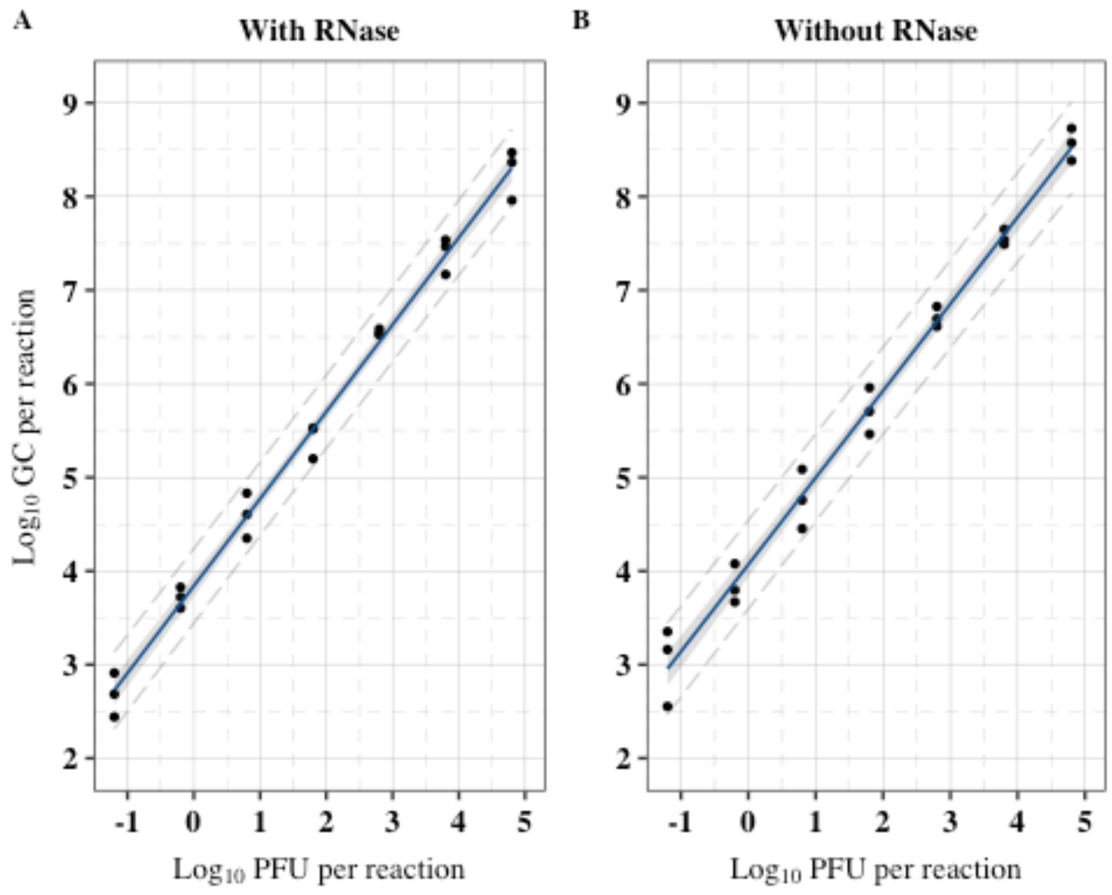


```
ggsave(plot=combined_plot, file="Fig5_TuV_gc_pfu.tiff", width=18, height=7,
units = c("cm"), dpi=600)
```

```r
rm(dat1, dat2, p_w, p_wo)
```

6. GC:PFU ratio

6.1 GC:PFU for w/ vs. w/o RNase

```r
# reading data for w/ and w/o RNase
dat1 <- readRDS("pred_GCfromPFU_ave.RDS")

# reading data for w/ and w/o RNase
dat1_w <- readRDS("pred_GCfromPFU_ave.RDS") |>
 dplyr::filter(rnase=="w") |>
 as.data.frame()

dat1_wo <- readRDS("pred_GCfromPFU_ave.RDS") |>
 dplyr::filter(rnase=="wo") |>
 as.data.frame()

# Average and std error
## W/ Rnase
mean(dat1_w$loggc2pfu)
```

```
[1] 3.72
```

```r
sd(dat1_w$loggc2pfu)/sqrt(length(dat1_w))
```

```
[1] 0.0657
```

```r
mean(dat1_w$gc2pfu)
```

```
[1] 5864
```

```r
sd(dat1_w$gc2pfu)/sqrt(length(dat1_w))
```

```
[1] 832
```

```r
## W/o Rnase
mean(dat1_wo$loggc2pfu)
```

```
[1] 3.94
```

```r
sd(dat1_wo$loggc2pfu)/sqrt(length(dat1_wo))
```

```
[1] 0.0744
```

```r
mean(dat1_wo$gc2pfu)
```

```
[1] 10525
```

```r
sd(dat1_wo$gc2pfu)/sqrt(length(dat1_wo))
```

```
[1] 2259

# Whether the GC:PFU different for w/o vs w/ RNase
dat1 <- readRDS("pred_GCfromPFU_ave.RDS")
dat1$pfu_rxn1 <- as.factor(dat1$pfu_rxn)
aov1 <- aov(loggc2pfu ~ rnase*pfu_rxn1, data = dat1);summary(aov1)

               Df Sum Sq Mean Sq F value Pr(>F)
rnase           1  0.524   0.524   10.47 0.0031 **
pfu_rxn1        6  0.897   0.150    2.99 0.0220 *
rnase:pfu_rxn1  6  0.064   0.011    0.21 0.9694
Residuals      28  1.401   0.050
---
Signif. codes:  0 '***' 0.001 '**' 0.01 '*' 0.05 '.' 0.1 ' ' 1

# Pairwise comparison by separating w/ and w/o rnase. pfu_rxn has to be a
factor
## W/ RNase
aov1 <- aov(loggc2pfu ~ pfu_rxn, data = dat1_w); summary(aov1)

            Df Sum Sq Mean Sq F value Pr(>F)
pfu_rxn      1  0.403   0.403    12.1 0.0025 **
Residuals   19  0.632   0.033
---
Signif. codes:  0 '***' 0.001 '**' 0.01 '*' 0.05 '.' 0.1 ' ' 1

pairwise.t.test(dat1_w$loggc2pfu, dat1_w$pfu_rxn,
                p.adjust.method = "bonf",
                alternative = c("two.sided"))


    Pairwise comparisons using t tests with pooled SD

data:  dat1_w$loggc2pfu and dat1_w$pfu_rxn

     -1.2 -0.2 0.8 1.8 2.8 3.8
-0.2 1.0  -    -   -   -   -
0.8  1.0  1.0  -   -   -   -
1.8  1.0  1.0  1.0 -   -   -
2.8  1.0  1.0  1.0 1.0 -   -
3.8  1.0  1.0  1.0 1.0 1.0 -
4.8  0.5  0.3  1.0 1.0 1.0 1.0

P value adjustment method: bonferroni

# W/O RNase
aov1 <- aov(loggc2pfu ~ pfu_rxn, data = dat1_wo); summary(aov1)

            Df Sum Sq Mean Sq F value Pr(>F)
pfu_rxn      1  0.431   0.431    9.14  0.007 **
Residuals   19  0.896   0.047
```

```
---
Signif. codes:  0 '***' 0.001 '**' 0.01 '*' 0.05 '.' 0.1 ' ' 1
```

```
pairwise.t.test(dat1_wo$loggc2pfu, dat1_wo$pfu_rxn,
                p.adjust.method = "bonf",
                alternative = c("two.sided"))
```

```

    Pairwise comparisons using t tests with pooled SD

data:  dat1_wo$loggc2pfu and dat1_wo$pfu_rxn

     -1.2 -0.2 0.8 1.8 2.8 3.8
-0.2 1.0  -    -   -   -   -
0.8  1.0  1.0  -   -   -   -
1.8  1.0  1.0  1.0 -   -   -
2.8  1.0  1.0  1.0 1.0 -   -
3.8  0.8  1.0  1.0 1.0 1.0 -
4.8  0.8  1.0  1.0 1.0 1.0 1.0

P value adjustment method: bonferroni
```

```
# Boxplot of GC:PFU across RNase
p <- ggplot(dat1, aes(x = factor(pfu_rxn), y = loggc2pfu)) +
  geom_boxplot(fill = "grey80", color = "black") +
  labs(x = "log10 PFU per Reaction",
       y = "logarithmic GC:PFU ratio",
       title = "GC:PFU ratio w/ and w/o RNase combined") +
  facet_wrap(~ rnase) +  # Separate the plots by the rnase variable
  theme_bw()

p
```

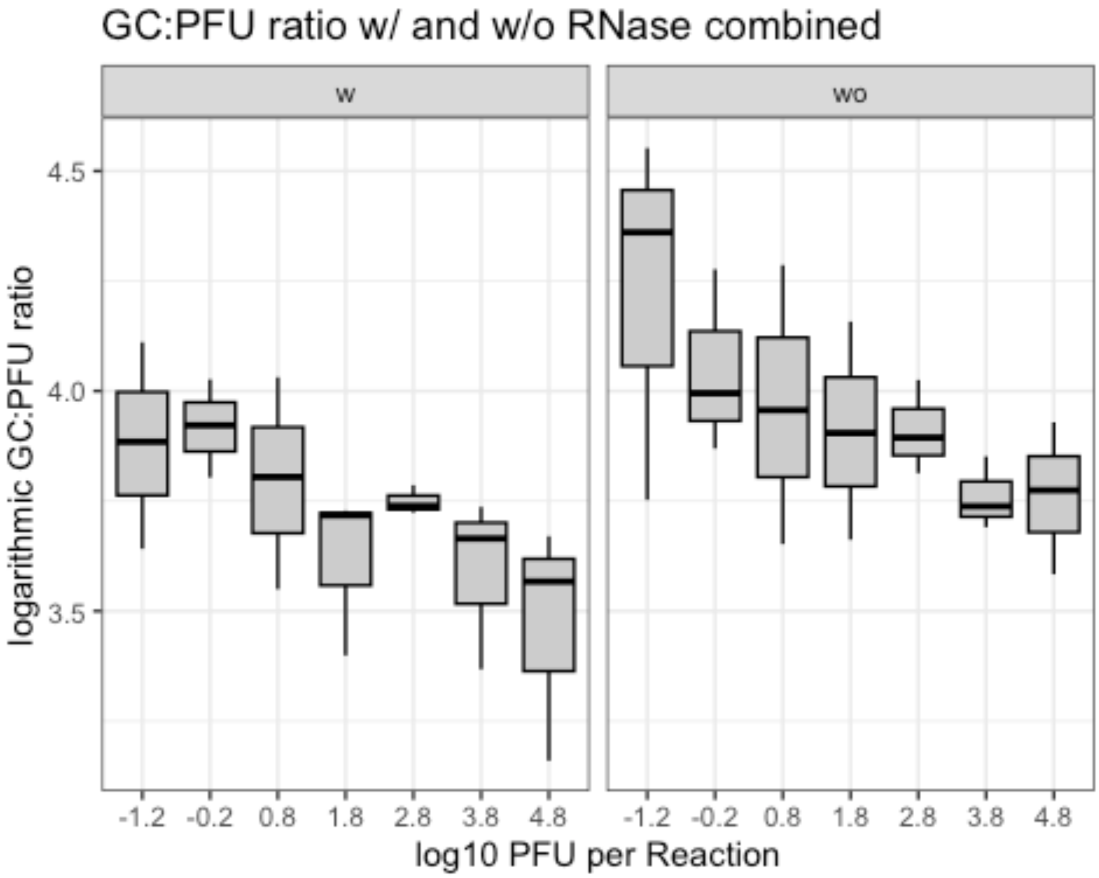


```
rm(dat1, dat1_w, dat1_wo, p, aov1)
```

6.2 Beta distributions

```
iters=1000
# W/ RNase
## reading data for w/ and w/o RNase
dat1_w <- readRDS("pred_GCfromPFU_ave.RDS") |>
 dplyr::filter(rnase=="w") |>
 as.data.frame()

# scaled value (min-max normalization)
min_loggc2pfu <- min(dat1_w$loggc2pfu)
max_loggc2pfu <- max(dat1_w$loggc2pfu)

dat1_w$loggc2pfu_s <- (dat1_w$loggc2pfu - min_loggc2pfu) / (max_loggc2pfu -
min_loggc2pfu)

# change absolute 0 and 1 to avoid error on modeling
dat1_w$loggc2pfu_s[dat1_w$loggc2pfu_s == 0] <- 0.00001
dat1_w$loggc2pfu_s[dat1_w$loggc2pfu_s == 1] <- 0.99999

# Beta dist fit: W/ RNase
fit_beta <- fitdist(dat1_w$loggc2pfu_s, "beta",
```

```
                    start = list(shape1 = 2, shape2 = 2),
                    method = 'mge'); summary (fit_beta)
```

```
Warning in fitdist(dat1_w$loggc2pfu_s, "beta", start = list(shape1 = 2, :
maximum GOF estimation has a default 'gof' argument set to 'CvM'

Fitting of the distribution ' beta ' by maximum goodness-of-fit
Parameters :
       estimate
shape1     3.72
shape2     2.58
Loglikelihood:  -36.9   AIC:  77.7   BIC:  79.8
```

```
# Bootstrap simulation of uncertainty
f_beta_boot <- bootdist(fit_beta, bootmethod="param", niter = iters)
summary(f_beta_boot)
```

```
Parametric bootstrap medians and 95% percentile CI
       Median 2.5% 97.5%
shape1   3.97 2.04  9.45
shape2   2.70 1.43  6.49

The estimation method converged only for 999 among 1000 iterations
```

```
# parameters of Beta distribution (scaled)
shape1_w <- quantile(f_beta_boot$estim[, 1], probs = c(0.5))
shape2_w <- quantile(f_beta_boot$estim[, 2], probs = c(0.5))

rbeta.est.boot_w <- rbeta (iters, shape1_w, shape2_w)

# Plotting density distributions
data_seq <- seq(0, 1, length = 100)
dbeta2 <- dbeta (data_seq, shape1_w, shape2_w)

# Create a data frame for the beta distribution to overlay
dat1_beta <- data.frame(x = data_seq, density = dbeta2)

scale_back <- function(x) {
  x * (max_loggc2pfu - min_loggc2pfu) + min_loggc2pfu
}

x_ticks_scaled <- seq(0, 1, length.out = 6)
x_ticks_actual <- scale_back (x_ticks_scaled)

# Summaries
beta_sum <- quantile(rbeta.est.boot_w, probs = c(0.025, 0.50, 0.975))
(beta_sum_actual <- scale_back (beta_sum))
```

```
 2.5%   50% 97.5%
 3.39  3.73  4.02
```

```
est_0.5 <- beta_sum[2]
est_0.5_actual <- beta_sum_actual[2]

p_w <- ggplot(data=dat1_w, aes(x = loggc2pfu_s)) +
 geom_histogram(aes(y = after_stat(density)), bins = 15,
               fill = "darkgray", color = "black") +
 geom_line(data=dat1_beta,
          aes(x = x, y = density),
          color = "#fb6502", linetype = "dashed", linewidth = 0.5) +
 geom_rug(aes(x = loggc2pfu_s), sides = "b", color = "black") +
 geom_vline(xintercept = est_0.5,
            color = "#00529b", linetype = "solid", linewidth = 0.5) +
 annotate("text", x = est_0.5,
         y = Inf, label = paste("Median =", round(est_0.5_actual, 2)),
         vjust = 5, hjust = -0.2,
         color = "#00529b", size = 2.5,
         family = "times") +
 scale_x_continuous(breaks = x_ticks_scaled,
                    labels = round(x_ticks_actual, 1)) +
 scale_y_continuous (breaks = c (0, 1, 2, 3, 4, 5),
                    limits = c(0, 5)) +
 labs(
  title = "With RNase",
  x = bquote (Log[10]~GC:PFU~ratio),
  y = "Density") +
 theme_bw (base_family="") +
 theme(
  text = element_text(family = "Times"),
  legend.position = "inside",
  legend.position.inside = c(0.3, 0.9),
  legend.direction="horizontal",
  legend.text=element_text(size = 6, face='bold'),
  legend.title=element_text (size = 6,face='bold'),
  plot.title = element_text(color="black", size=10, face="bold", hjust = 0.5,
vjust=0.5),
  axis.text.x=element_text(size = 10, color="black", face="bold", hjust =
0.5, vjust = 0.5),
  axis.text.y=element_text (size = 10, color="black", face="bold"),
  axis.title.x = element_text (size = 10, vjust = -1),
  axis.title.y = element_text (size = 10, vjust = 2),
  panel.grid.major = element_line (color="darkgray", linewidth=0.1,
linetype="solid"),
  panel.grid.minor = element_line (color="gray", linewidth=0.1,
linetype="dashed"),
  panel.background = element_rect (fill =  "transparent"),
  legend.box.background=element_rect(fill="transparent",
color="transparent"),
  legend.background = element_rect (fill = "transparent", color =
"transparent"),
  legend.key=element_rect(fill="transparent", color="transparent"),
```

```
  plot.background = element_rect (fill = "transparent", color =
"transparent")) +
 guides(color=guide_legend(override.aes=list(fill=NA)))

p_w
```

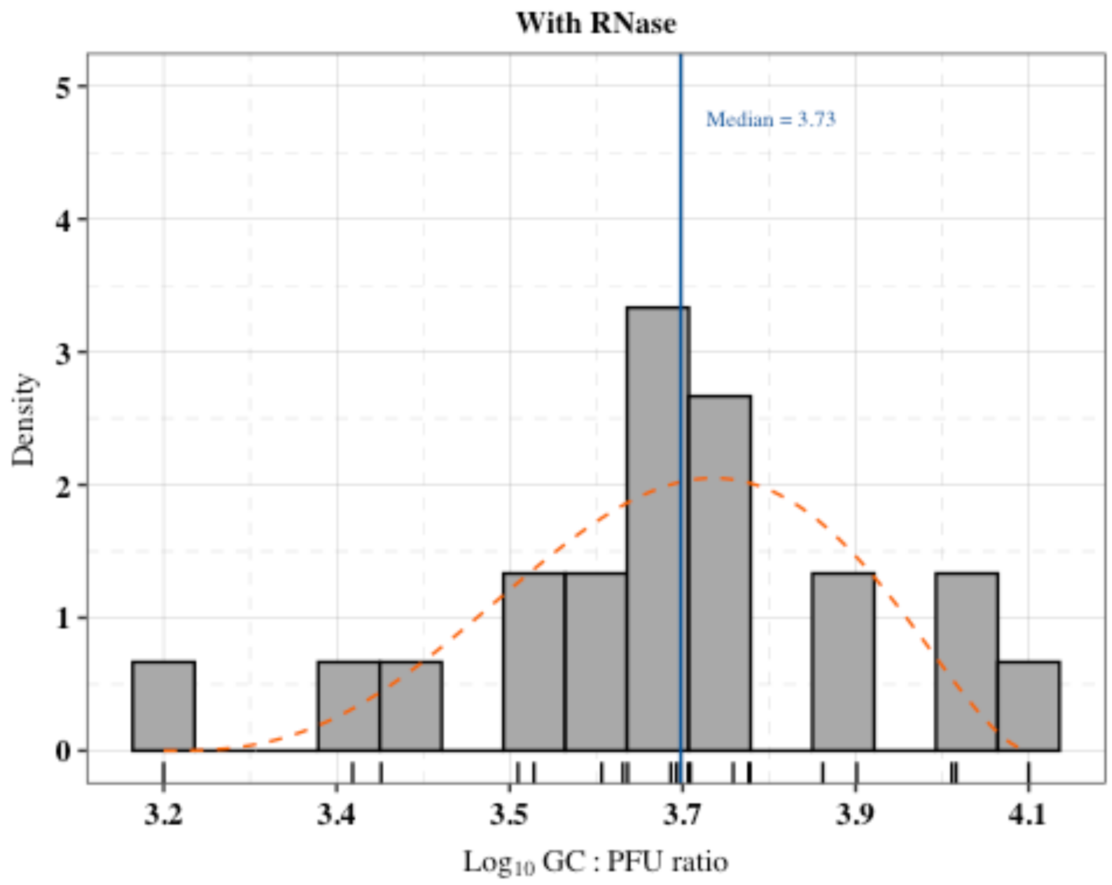


```
####
# W/o RNase
## reading data for w/ and w/o RNase
dat1_wo <- readRDS("pred_GCfromPFU_ave.RDS") |>
 dplyr::filter(rnase=="wo") |>
 as.data.frame()

# scaled value (min-max normalization)
min_loggc2pfu <- min(dat1_wo$loggc2pfu)
max_loggc2pfu <- max(dat1_wo$loggc2pfu)

dat1_wo$loggc2pfu_s <- (10^dat1_wo$loggc2pfu - 10^min_loggc2pfu) /
(10^max_loggc2pfu - 10^min_loggc2pfu)

# Convert absolute 0 and 1 values to avoid calculation error
dat1_wo$loggc2pfu_s[dat1_wo$loggc2pfu_s == 0] <- 0.00001
dat1_wo$loggc2pfu_s[dat1_wo$loggc2pfu_s == 1] <- 0.99999
```

```
# Beta dist fit: W/o RNase
fit_beta <- fitdist(dat1_wo$loggc2pfu_s, "beta",
                    start = list(shape1 = 2, shape2 = 2),
                    method = 'mge'); summary (fit_beta)

Warning in fitdist(dat1_wo$loggc2pfu_s, "beta", start = list(shape1 = 2, :
maximum GOF estimation has a default 'gof' argument set to 'CvM'

Fitting of the distribution ' beta ' by maximum goodness-of-fit
Parameters :
       estimate
shape1    0.879
shape2    4.286
Loglikelihood:  -20.7   AIC:  45.3   BIC:  47.4

# Bootstrap simulation of uncertainty
f_beta_boot <- bootdist(fit_beta, bootmethod="param", niter = iters)
summary(f_beta_boot)

Parametric bootstrap medians and 95% percentile CI
       Median  2.5% 97.5%
shape1  0.919 0.545  2.01
shape2  4.618 2.251 11.88

shape1_wo <- quantile(f_beta_boot$estim[, 1], probs = c(0.5))
shape2_wo <- quantile(f_beta_boot$estim[, 2], probs = c(0.5))

rbeta.est.boot_wo <- rbeta (iters, shape1_wo, shape2_wo)

# Plotting density distributions
data_seq <- seq(0, 1, length = 100)
dbeta2 <- dbeta (data_seq, shape1_wo, shape2_wo)

# Create a data frame for the beta distribution to overlay
dat1_beta <- data.frame(x = data_seq, density = dbeta2)

scale_back <- function(x) {
  x * (max_loggc2pfu - min_loggc2pfu) + min_loggc2pfu
}

x_ticks_scaled <- seq(0, 1, length.out = 6)
x_ticks_actual <- scale_back(x_ticks_scaled)

# Summaries
beta_sum <- quantile(rbeta.est.boot_wo, prob=c(0.025, 0.50, 0.975))
(beta_sum_actual <- scale_back (beta_sum))

 2.5%   50% 97.5%
 3.59  3.72  4.10
```

```
est_0.5 <- beta_sum[2]
est_0.5_actual <- beta_sum_actual[2]

p_wo <- ggplot(data=dat1_wo, aes(x = loggc2pfu_s)) +
 geom_histogram(aes(y = after_stat(density)), bins = 15,
                fill = "darkgray", color = "black") +
 geom_line(data=dat1_beta,
          aes(x = x, y = density),
          color = "#fb6502", linetype = "dashed", linewidth = 0.5) +
 geom_rug(aes(x = loggc2pfu_s), sides = "b", color = "black") +
 geom_vline(xintercept = est_0.5,
            color = "#00529b", linetype = "solid", linewidth = 0.5) +
 annotate("text", x = est_0.5,
         y = Inf, label = paste("Median =", round(est_0.5_actual, 2)),
         vjust = 5, hjust = -0.2,
         color = "#00529b", size = 2.5,
         family = "times") +
 scale_x_continuous(breaks = x_ticks_scaled,
                    labels = round(x_ticks_actual, 1)) +
 scale_y_continuous (breaks = c (0, 1, 2, 3, 4, 5),
                    limits = c(0, 5)) +
 labs(
   title = "Without RNase",
       x = bquote (Log[10]~GC:PFU~ratio),
       y = "") +
 theme_bw (base_family="") +
 theme(
  text = element_text(family = "Times"),
  legend.position = "inside",
  legend.position.inside = c(0.3, 0.9),
  legend.direction="horizontal",
  legend.text=element_text(size = 6, face='bold'),
  legend.title=element_text (size = 6,face='bold'),
  plot.title = element_text(color="black", size=10, face="bold", hjust = 0.5,
vjust=0.5),
  axis.text.x=element_text(size = 10, color="black", face="bold", hjust =
0.5, vjust = 0.5),
  axis.text.y=element_text (size = 10, color="black", face="bold"),
  axis.title.x = element_text (size = 10, vjust = -1),
  axis.title.y = element_text (size = 10, vjust = 2),
  panel.grid.major = element_line (color="darkgray", linewidth=0.1,
linetype="solid"),
  panel.grid.minor = element_line (color="gray", linewidth=0.1,
linetype="dashed"),
  panel.background = element_rect (fill =  "transparent"),
  legend.box.background=element_rect(fill="transparent",
color="transparent"),
  legend.background = element_rect (fill = "transparent", color =
"transparent"),
  legend.key=element_rect(fill="transparent", color="transparent"),
```

```
  plot.background = element_rect (fill = "transparent", color =
"transparent")) +
 guides(color=guide_legend(override.aes=list(fill=NA)))

combined_plot <- ggpubr::ggarrange(p_w, p_wo,
                                   ncol = 2,
                                   labels = c("A", "B"),
                                   font.label = list(size=9, face="bold",
family="times"
                                   ))
combined_plot
```

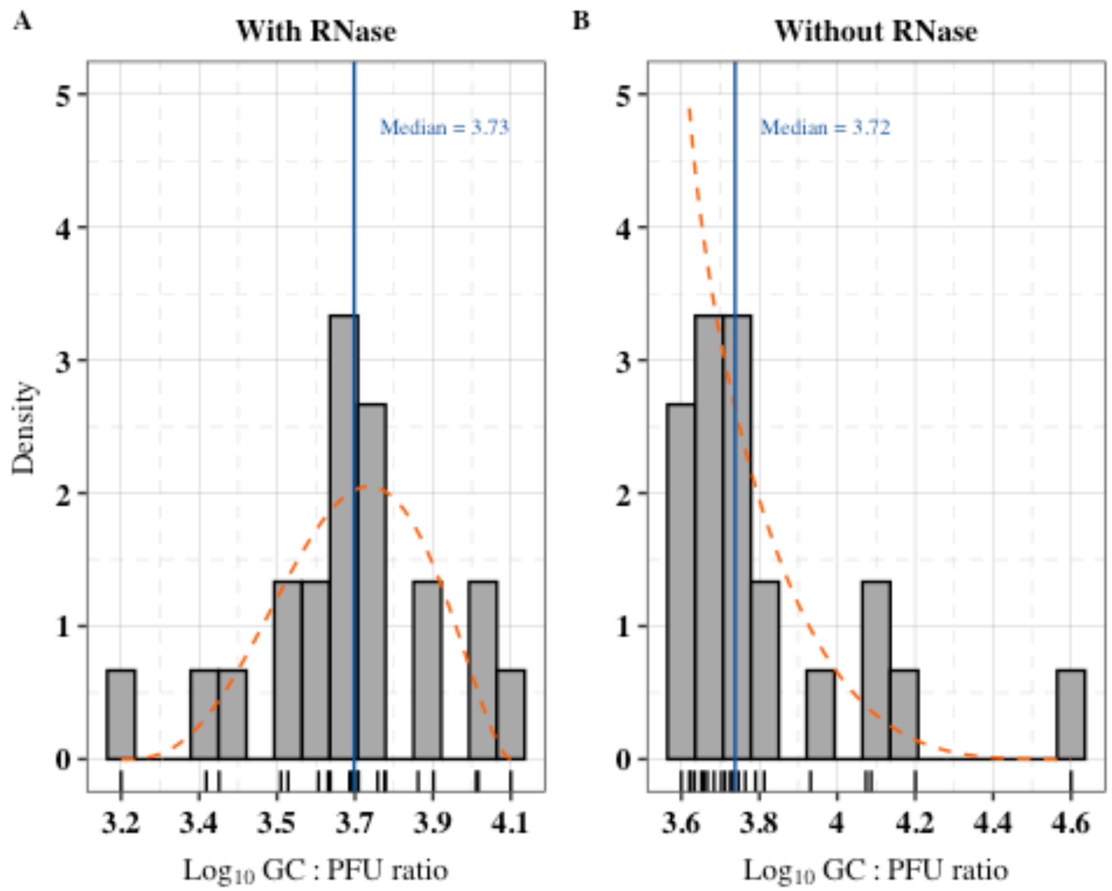


```
ggsave(plot=combined_plot, file="Fig6_GC2PFU_beta_dist.tiff", width=18,
height=7, units = c("cm"), dpi=600)

# Compare distributions
## Asymptotic two-sample Kolmogorov-Smirnov test
ks_out <- ks.test(rbeta.est.boot_w, rbeta.est.boot_wo, alternative =
"two.sided")
ks_out


    Asymptotic two-sample Kolmogorov-Smirnov test
```

```
data:  rbeta.est.boot_w and rbeta.est.boot_wo
D = 0.8, p-value <2e-16
alternative hypothesis: two-sided
```

```
## Wilcoxon rank sum test: comparing locations
wx_out <- wilcox.test(rbeta.est.boot_w, rbeta.est.boot_wo, alternative =
"two.sided",
                      conf.int = TRUE, conf.level = 0.95)
wx_out
```

```

    Wilcoxon rank sum test with continuity correction

data:  rbeta.est.boot_w and rbeta.est.boot_wo
W = 1e+06, p-value <2e-16
alternative hypothesis: true location shift is not equal to 0
95 percent confidence interval:
 0.415 0.445
sample estimates:
difference in location
                  0.43
```

```
rm(dat1_wo, iters, fit_beta, shape1_wo, shape2_wo, f_beta_boot, dbeta2,
data_seq, dat1_beta, rbeta.est.boot_w, rbeta.est.boot_wo, combined_plot,
ks_out, wx_out, est_0.5, est_0.5_actual, x_ticks_actual, x_ticks_scaled,
scale_back, dat1_w, shape1_w, shape2_w)
```

6.3 PFU over GC:PFU ratio predictive power

6.3.1 PFU ~ GC:PFU

```
# Read data
dat1 <- readRDS("pred_GCfromPFU_ave.RDS")
dat1 <- dat1[complete.cases(dat1), ]

# Subset w/ and wo RNase
dat1_w <- dat1 |>
 dplyr::filter(rnase=="w") |>
 as.data.frame()

dat1_wo <- dat1 |>
 dplyr::filter(rnase=="wo") |>
 as.data.frame()

# Fitting the model and predict using global models
# W/ Rnase
fit_w <- lm(pfu_rxn ~ loggc2pfu, data = dat1_w); summary(fit_w)
```

```

Call:
```

```
lm(formula = pfu_rxn ~ loggc2pfu, data = dat1_w)

Residuals:
   Min     1Q Median     3Q    Max 
-3.418 -0.841  0.009  1.130  2.743 

Coefficients:
            Estimate Std. Error t value Pr(>|t|)   
(Intercept)    22.69       6.01    3.78   0.0013 **
loggc2pfu      -5.62       1.61   -3.48   0.0025 **
---
Signif. codes:  0 '***' 0.001 '**' 0.01 '*' 0.05 '.' 0.1 ' ' 1

Residual standard error: 1.64 on 19 degrees of freedom
Multiple R-squared:  0.39,  Adjusted R-squared:  0.358 
F-statistic: 12.1 on 1 and 19 DF,  p-value: 0.00249
```

```
sqrt(mean(fit_w$residuals^2))
```

```
[1] 1.56
```

```
coef(fit_w)
```

```
(Intercept)   loggc2pfu 
      22.69       -5.62 
```

```
confint(fit_w)
```

```
            2.5 % 97.5 %
(Intercept)  10.1  35.27
loggc2pfu    -9.0  -2.24
```

```
# W/o Rnase
fit_wo <- lm(pfu_rxn ~ loggc2pfu, data = dat1_wo); summary(fit_wo)
```

```
Call:
lm(formula = pfu_rxn ~ loggc2pfu, data = dat1_wo)

Residuals:
   Min     1Q Median     3Q    Max 
-3.847 -1.090  0.429  1.088  2.953 

Coefficients:
            Estimate Std. Error t value Pr(>|t|)   
(Intercept)    19.66       5.92    3.32   0.0036 **
loggc2pfu      -4.53       1.50   -3.02   0.0070 **
---
Signif. codes:  0 '***' 0.001 '**' 0.01 '*' 0.05 '.' 0.1 ' ' 1

Residual standard error: 1.73 on 19 degrees of freedom
```

```
Multiple R-squared:  0.325, Adjusted R-squared:  0.289
F-statistic: 9.14 on 1 and 19 DF,  p-value: 0.00699
```

```
sqrt(mean(fit_wo$residuals^2))
```

```
[1] 1.64
```

```
coef(fit_wo)
```

```
(Intercept)   loggc2pfu
      19.66       -4.53
```

```
confint(fit_wo)
```

```
            2.5 % 97.5 %
(Intercept)  7.27   32.0
loggc2pfu   -7.67   -1.4
```

```
list(fit_w=fit_w, fit_ws=summary(fit_w),
     fit_wo=fit_wo, fit_wos=summary(fit_wo))|>
 saveRDS("logGC2PFU_to_PFU_model_fits.RDS")

rm(dat1, dat1_w, dat1_wo, fit_w, fit_wo)
```

6.3.2 PFU ~ log GC:PFU predictions

```
# read data and model fit
dat1 <- readRDS("pred_GCfromPFU_ave.RDS")
dat1 <- dat1[complete.cases(dat1), ]

model_fits <- readRDS("logGC2PFU_to_PFU_model_fits.RDS")

# Create data sequence
data_seq <- data.frame(loggc2pfu=seq(min(dat1$loggc2pfu),
max(dat1$loggc2pfu), length.out = 50))

## W/ RNase
pred_fit_w <- predict(model_fits$fit_w,
                      newdata = data_seq,
                      interval = "prediction", level = 0.95) |>
 as.data.frame() |>
 rename(pred_lwr_w = lwr,
        pred_upr_w = upr,
        pred_fit_w = fit)

ci_fit_w <- predict(model_fits$fit_w,
                      newdata = data_seq,
                      interval = "confidence", level = 0.95)  |>
 as.data.frame() |>
 rename(ci_lwr_w = lwr,
        ci_upr_w = upr,
        ci_fit_w = fit)
```

```
## W/o RNase
pred_fit_wo <- predict(model_fits$fit_wo,
                        newdata = data_seq,
                        interval = "prediction", level = 0.95) |>
 as.data.frame() |>
 rename(pred_lwr_wo = lwr,
        pred_upr_wo = upr,
        pred_fit_wo = fit)

ci_fit_wo <- predict(model_fits$fit_wo,
                        newdata = data_seq,
                        interval = "confidence", level = 0.95)  |>
 as.data.frame() |>
 rename(ci_lwr_wo = lwr,
        ci_upr_wo = upr,
        ci_fit_wo = fit)

merged_data <- cbind(data_seq, pred_fit_w, ci_fit_w, pred_fit_wo, ci_fit_w)

merged_data$pred_dif_wo_w <- merged_data$pred_fit_wo - merged_data$pred_fit_w

saveRDS(merged_data, "logGC2PFU_to_PFU_model_prediciton.RDS")

rm(dat1, data_seq, model_fits, ci_fit_w, ci_fit_wo, pred_fit_wo, pred_fit_w,
merged_data)
```

6.3.3 Models agreement analyses and visualizations

```
# Bland-Altman analysis (Tukey mean-difference)
dat1 <- readRDS("logGC2PFU_to_PFU_model_prediciton.RDS")

# W/ RNase
blandr.statistics (dat1$pred_fit_wo, dat1$pred_fit_w, sig.level=0.95)

Bland-Altman Statistics
=======================
t = 20, df = 49, p-value = <2e-16
alternative hypothesis: true bias is not equal to 0

=======================
Number of comparisons:  50
Maximum value for average measures:  5.13
Minimum value for average measures:  -1.94
Maximum value for difference in measures:  1.92
Minimum value for difference in measures:  0.408

Bias:  1.17
Standard deviation of bias:  0.451
```

```
Standard error of bias:  0.0638
Standard error for limits of agreement:  0.11

Bias:  1.17
Bias- upper 95% CI:  1.29
Bias- lower 95% CI:  1.04

Upper limit of agreement:  2.05
Upper LOA- upper 95% CI:  2.27
Upper LOA- lower 95% CI:  1.83

Lower limit of agreement:  0.282
Lower LOA- upper 95% CI:  0.502
Lower LOA- lower 95% CI:  0.061

=======================
Derived measures:
Mean of differences/means:  -13
Point estimate of bias as proportion of lowest average:  -60.1
Point estimate of bias as proportion of highest average 22.7
Spread of data between lower and upper LoAs:  1.77
Bias as proportion of LoA spread:  65.9

=======================
Bias:
 1.17  ( 1.04  to  1.29 )
ULoA:
 2.05  ( 1.83  to  2.27 )
LLoA:
 0.282  ( 0.061  to  0.502 )
```

```
# Difference between W/O over W/ RNase treatment
mean(dat1$pred_dif_wo_w)
```

```
[1] 1.17
```

```
sd(dat1$pred_dif_wo_w)/sqrt(length(dat1))
```

```
[1] 0.121
```

```
mean(10^dat1$pred_dif_wo_w)
```

```
[1] 23.7
```

```
sd(10^dat1$pred_dif_wo_w)/sqrt(length(dat1))
```

```
[1] 6.02
```

```
## check the assumption of normality for the differences
shapiro.test(dat1$pred_dif_wo_w)
```

```
    Shapiro-Wilk normality test

data:  dat1$pred_dif_wo_w
W = 1, p-value = 0.06
```

```
## t-test
t.test(
 dat1$pred_fit_wo, dat1$pred_fit_w,
 alternative = c("two.sided"),
 paired = TRUE)
```

```
    Paired t-test

data:  dat1$pred_fit_wo and dat1$pred_fit_w
t = 18, df = 49, p-value <2e-16
alternative hypothesis: true mean difference is not equal to 0
95 percent confidence interval:
 1.04 1.29
sample estimates:
mean difference
           1.17
```

6.3.4 Model validation through train data (using original data)

```
dat1 <- readRDS("pred_GCfromPFU_ave.RDS")
dat1 <- dat1[complete.cases(dat1), ]

# Initialize a list to store the RMSE results
rmse_results <- list()
r2_results <- list()
train_data_list <- list()
test_data_list <- list()

# Train data w/ RNase
dat1_w <- dat1 |>
  filter(rnase=="w")

index <- dat1_w$loggc2pfu |>
  createDataPartition(p = 0.70, times = 5, list = TRUE)

# Loop over each partition and check for data size
for (i in 1:length(index)) {
  train_data <- dat1_w[index[[i]], ]
  test_data <- dat1_w[-index[[i]], ]

  # Check if both train and test sets have more than one row
  if (nrow(train_data) > 1 & nrow(test_data) > 1) {
    # Train the model on train_data
    model <- train(pfu_rxn ~ loggc2pfu,
```

```r
                   data = train_data,
                   method = "lm",
                   trControl = trainControl(method = "boot", number = 500))

    # Predict on test data
    pred <- predict(model, newdata = test_data)

    # Calculate RMSE
    rmse <- caret::RMSE(pred = pred, obs = test_data$pfu_rxn)
    r2 <- caret::R2(pred = pred, obs = test_data$pfu_rxn)

    # Store the result
    rmse_results[[i]] <- rmse
    r2_results[[i]] <- r2
    train_data_list[[i]] <- train_data
    test_data_list[[i]] <- test_data

  } else {
    # If either train or test set is too small, skip this iteration
    cat("Skipping iteration", i, "due to small data size\n")
  }
}
```

```
Warning in nominalTrainWorkflow(x = x, y = y, wts = weights, info =
trainInfo,
: There were missing values in resampled performance measures.
Warning in nominalTrainWorkflow(x = x, y = y, wts = weights, info =
trainInfo,
: There were missing values in resampled performance measures.
```

```r
# Check if any valid RMSE results were obtained
rmse_results; summary(unlist(rmse_results))
```

```
[[1]]
[1] 1.35

[[2]]
[1] 1.24

[[3]]
[1] 1.06

[[4]]
[1] 1.83

[[5]]
[1] 1.17

   Min. 1st Qu.  Median    Mean 3rd Qu.    Max.
   1.06    1.17    1.24    1.33    1.35    1.83
```

```
r2_results; summary(unlist(r2_results))
```

```
[[1]]
[1] 0.507

[[2]]
[1] 0.283

[[3]]
[1] 0.834

[[4]]
[1] 0.622

[[5]]
[1] 0.933

   Min. 1st Qu.  Median    Mean 3rd Qu.    Max. 
  0.283   0.507   0.622   0.636   0.834   0.933 
```

```
train_data_list[1]
```

```
[[1]]
   dil pfu_rxn rnase rep pred_fit pred_lwr pred_upr ci_fit ci_lwr ci_upr
1    0     4.8     w   1     8.37     8.09     8.64   8.37   8.30   8.43
2    0     4.8     w   2     7.96     7.69     8.23   7.96   7.90   8.02
3    0     4.8     w   3     8.47     8.19     8.75   8.47   8.40   8.54
4    1     3.8     w   1     7.17     6.90     7.44   7.17   7.12   7.21
5    1     3.8     w   2     7.54     7.26     7.81   7.54   7.49   7.59
7    2     2.8     w   1     6.52     6.25     6.79   6.52   6.48   6.56
8    2     2.8     w   2     6.59     6.32     6.86   6.59   6.55   6.63
9    2     2.8     w   3     6.54     6.27     6.81   6.54   6.50   6.58
10   3     1.8     w   1     5.52     5.25     5.79   5.52   5.48   5.55
12   3     1.8     w   3     5.53     5.26     5.80   5.53   5.49   5.56
13   4     0.8     w   1     4.35     4.08     4.62   4.35   4.30   4.39
15   4     0.8     w   3     4.83     4.56     5.10   4.83   4.79   4.87
16   5    -0.2     w   1     3.72     3.45     4.00   3.72   3.67   3.78
17   5    -0.2     w   2     3.60     3.33     3.88   3.60   3.55   3.66
18   5    -0.2     w   3     3.83     3.55     4.10   3.83   3.77   3.88
19   6    -1.2     w   1     2.44     2.16     2.72   2.44   2.37   2.52
20   6    -1.2     w   2     2.68     2.41     2.96   2.68   2.61   2.76
   loggc2pfu gc2pfu
1       3.57   3691
2       3.16   1445
3       3.67   4680
4       3.37   2332
5       3.74   5453
7       3.72   5293
8       3.79   6112
9       3.74   5482
```

```
10       3.72   5219
12       3.73   5346
13       3.55   3545
15       4.03  10746
16       3.92   8357
17       3.80   6358
18       4.03  10630
19       3.64   4381
20       3.88   7668
```

test_data_list

```
[[1]]
   dil pfu_rxn rnase rep pred_fit pred_lwr pred_upr ci_fit ci_lwr ci_upr
6    1     3.8     w   3     7.46     7.19     7.74   7.46   7.42   7.51
11   3     1.8     w   2     5.20     4.93     5.47   5.20   5.16   5.24
14   4     0.8     w   2     4.60     4.33     4.88   4.60   4.56   4.65
21   6    -1.2     w   3     2.91     2.63     3.19   2.91   2.84   2.98
   loggc2pfu gc2pfu
6       3.66   4623
11      3.40   2506
14      3.80   6376
21      4.11  12911

[[2]]
   dil pfu_rxn rnase rep pred_fit pred_lwr pred_upr ci_fit ci_lwr ci_upr
10   3     1.8     w   1     5.52     5.25     5.79   5.52   5.48   5.55
13   4     0.8     w   1     4.35     4.08     4.62   4.35   4.30   4.39
14   4     0.8     w   2     4.60     4.33     4.88   4.60   4.56   4.65
16   5    -0.2     w   1     3.72     3.45     4.00   3.72   3.67   3.78
   loggc2pfu gc2pfu
10      3.72   5219
13      3.55   3545
14      3.80   6376
16      3.92   8357

[[3]]
   dil pfu_rxn rnase rep pred_fit pred_lwr pred_upr ci_fit ci_lwr ci_upr
4    1     3.8     w   1     7.17     6.90     7.44   7.17   7.12   7.21
6    1     3.8     w   3     7.46     7.19     7.74   7.46   7.42   7.51
14   4     0.8     w   2     4.60     4.33     4.88   4.60   4.56   4.65
21   6    -1.2     w   3     2.91     2.63     3.19   2.91   2.84   2.98
   loggc2pfu gc2pfu
4       3.37   2332
6       3.66   4623
14      3.80   6376
21      4.11  12911

[[4]]
   dil pfu_rxn rnase rep pred_fit pred_lwr pred_upr ci_fit ci_lwr ci_upr
```

```
10   3     1.8     w   1     5.52     5.25     5.79   5.52   5.48   5.55
11   3     1.8     w   2     5.20     4.93     5.47   5.20   5.16   5.24
17   5    -0.2     w   2     3.60     3.33     3.88   3.60   3.55   3.66
20   6    -1.2     w   2     2.68     2.41     2.96   2.68   2.61   2.76
   loggc2pfu gc2pfu
10      3.72   5219
11      3.40   2506
17      3.80   6358
20      3.88   7668

[[5]]
   dil pfu_rxn rnase rep pred_fit pred_lwr pred_upr ci_fit ci_lwr ci_upr
2    0     4.8     w   2     7.96     7.69     8.23   7.96   7.90   8.02
7    2     2.8     w   1     6.52     6.25     6.79   6.52   6.48   6.56
8    2     2.8     w   2     6.59     6.32     6.86   6.59   6.55   6.63
15   4     0.8     w   3     4.83     4.56     5.10   4.83   4.79   4.87
   loggc2pfu gc2pfu
2       3.16   1445
7       3.72   5293
8       3.79   6112
15      4.03  10746
```

```
rm(dat1_w, r2_results, rmse_results, test_data, train_data)

######
# W/o RNase
# Train data w/ RNase
dat1_wo <- dat1 |>
  filter(rnase=="wo")

rmse_results <- list()
r2_results <- list()
train_data_list <- list()
test_data_list <- list()

index <- dat1_wo$loggc2pfu |>
  createDataPartition(p = 0.70, times = 5, list = TRUE)

# Loop over each partition and check for data size
for (i in 1:length(index)) {
  train_data <- dat1_wo[index[[i]], ]
  test_data <- dat1_wo[-index[[i]], ]

  # Check if both train and test sets have more than one row
  if (nrow(train_data) > 1 & nrow(test_data) > 1) {
    # Train the model on train_data
    model <- train(pfu_rxn ~ loggc2pfu,
                   data = train_data,
                   method = "lm",
```

```
                   trControl = trainControl(method = "boot", number = 500))

    # Predict on test data
    pred <- predict(model, newdata = test_data)

    # Calculate RMSE
    rmse <- caret::RMSE(pred = pred, obs = test_data$pfu_rxn)
    r2 <- caret::R2(pred = pred, obs = test_data$pfu_rxn)

    # Store the result
    rmse_results[[i]] <- rmse
    r2_results[[i]] <- r2
    train_data_list[[i]] <- train_data
    test_data_list[[i]] <- test_data

  } else {
    # If either train or test set is too small, skip this iteration
    cat("Skipping iteration", i, "due to small data size\n")
  }
}

# Check if any valid RMSE results were obtained
rmse_results; summary(unlist(rmse_results))

[[1]]
[1] 1.63

[[2]]
[1] 2.1

[[3]]
[1] 1.71

[[4]]
[1] 1.76

[[5]]
[1] 0.956

   Min. 1st Qu.  Median    Mean 3rd Qu.    Max.
  0.956   1.632   1.705   1.631   1.764   2.100

r2_results; summary(unlist(r2_results))

[[1]]
[1] 0.626

[[2]]
[1] 0.32
```

```
[[3]]
[1] 0.86

[[4]]
[1] 0.0578

[[5]]
[1] 0.98

   Min. 1st Qu.  Median    Mean 3rd Qu.    Max. 
  0.058   0.320   0.626   0.569   0.860   0.980 

train_data_list[1]

[[1]]
   dil pfu_rxn rnase rep pred_fit pred_lwr pred_upr ci_fit ci_lwr ci_upr
1    0     4.8    wo   1     8.38     8.11     8.66   8.38   8.32   8.45
2    0     4.8    wo   2     8.57     8.30     8.85   8.57   8.51   8.64
3    0     4.8    wo   3     8.73     8.45     9.01   8.73   8.66   8.80
5    1     3.8    wo   2     7.65     7.38     7.92   7.65   7.60   7.70
6    1     3.8    wo   3     7.49     7.22     7.76   7.49   7.44   7.54
7    2     2.8    wo   1     6.61     6.34     6.88   6.61   6.57   6.65
8    2     2.8    wo   2     6.69     6.42     6.97   6.69   6.65   6.74
9    2     2.8    wo   3     6.83     6.55     7.10   6.83   6.78   6.87
10   3     1.8    wo   1     5.46     5.19     5.73   5.46   5.43   5.50
11   3     1.8    wo   2     5.70     5.43     5.98   5.70   5.67   5.74
12   3     1.8    wo   3     5.96     5.69     6.23   5.96   5.92   5.99
13   4     0.8    wo   1     4.45     4.18     4.72   4.45   4.41   4.50
14   4     0.8    wo   2     5.09     4.82     5.36   5.09   5.05   5.12
17   5    -0.2    wo   2     3.80     3.52     4.07   3.80   3.74   3.85
18   5    -0.2    wo   3     4.08     3.80     4.35   4.08   4.03   4.13
19   6    -1.2    wo   1     2.55     2.27     2.83   2.55   2.48   2.63
21   6    -1.2    wo   3     3.35     3.08     3.63   3.35   3.29   3.41
   loggc2pfu gc2pfu
1       3.58   3835
2       3.77   5950
3       3.93   8490
5       3.85   7105
6       3.69   4905
7       3.81   6507
8       3.89   7839
9       4.03  10597
10      3.66   4594
11      3.90   8029
12      4.16  14379
13      3.65   4491
14      4.29  19324
17      4.00   9890
18      4.28  18913
```

```
19       3.75   5656
21       4.55  35655
```

test_data_list

```
[[1]]
   dil pfu_rxn rnase rep pred_fit pred_lwr pred_upr ci_fit ci_lwr ci_upr
4    1     3.8    wo   1     7.54     7.27     7.81   7.54   7.49   7.59
15   4     0.8    wo   3     4.76     4.49     5.03   4.76   4.72   4.80
16   5    -0.2    wo   1     3.67     3.40     3.94   3.67   3.61   3.72
20   6    -1.2    wo   2     3.16     2.89     3.44   3.16   3.10   3.22
   loggc2pfu gc2pfu
4       3.74   5473
15      3.96   9049
16      3.87   7402
20      4.36  22942

[[2]]
   dil pfu_rxn rnase rep pred_fit pred_lwr pred_upr ci_fit ci_lwr ci_upr
3    0     4.8    wo   3     8.73     8.45     9.01   8.73   8.66   8.80
5    1     3.8    wo   2     7.65     7.38     7.92   7.65   7.60   7.70
13   4     0.8    wo   1     4.45     4.18     4.72   4.45   4.41   4.50
21   6    -1.2    wo   3     3.35     3.08     3.63   3.35   3.29   3.41
   loggc2pfu gc2pfu
3       3.93   8490
5       3.85   7105
13      3.65   4491
21      4.55  35655

[[3]]
   dil pfu_rxn rnase rep pred_fit pred_lwr pred_upr ci_fit ci_lwr ci_upr
2    0     4.8    wo   2     8.57     8.30     8.85   8.57   8.51   8.64
6    1     3.8    wo   3     7.49     7.22     7.76   7.49   7.44   7.54
9    2     2.8    wo   3     6.83     6.55     7.10   6.83   6.78   6.87
14   4     0.8    wo   2     5.09     4.82     5.36   5.09   5.05   5.12
   loggc2pfu gc2pfu
2       3.77   5950
6       3.69   4905
9       4.03  10597
14      4.29  19324

[[4]]
   dil pfu_rxn rnase rep pred_fit pred_lwr pred_upr ci_fit ci_lwr ci_upr
5    1     3.8    wo   2     7.65     7.38     7.92   7.65   7.60   7.70
13   4     0.8    wo   1     4.45     4.18     4.72   4.45   4.41   4.50
14   4     0.8    wo   2     5.09     4.82     5.36   5.09   5.05   5.12
17   5    -0.2    wo   2     3.80     3.52     4.07   3.80   3.74   3.85
   loggc2pfu gc2pfu
5       3.85   7105
13      3.65   4491
```

```
14       4.29  19324
17       4.00   9890

[[5]]
   dil pfu_rxn rnase rep pred_fit pred_lwr pred_upr ci_fit ci_lwr ci_upr
4    1     3.8    wo   1     7.54     7.27     7.81   7.54   7.49   7.59
7    2     2.8    wo   1     6.61     6.34     6.88   6.61   6.57   6.65
11   3     1.8    wo   2     5.70     5.43     5.98   5.70   5.67   5.74
20   6    -1.2    wo   2     3.16     2.89     3.44   3.16   3.10   3.22
   loggc2pfu gc2pfu
4       3.74   5473
7       3.81   6507
11      3.90   8029
20      4.36  22942
```
